# Genome-Wide Identification and Expression Pattern of Sugar Transporter Genes in the Brown Planthopper, *Nilaparvata lugens* (Stål)

**DOI:** 10.3390/insects15070509

**Published:** 2024-07-07

**Authors:** Xinxin Shangguan, Xiaoyu Yang, Siyin Wang, Lijie Geng, Lina Wang, Mengfan Zhao, Haohao Cao, Yi Zhang, Xiaoli Li, Mingsheng Yang, Kedong Xu, Xiaohong Zheng

**Affiliations:** 1 Key Laboratory of Plant Genetics and Molecular Breeding, Zhoukou Normal University, Zhoukou 466001, China; 2 Henan Key Laboratory of Crop Molecular Breeding & Bioreactor, Zhoukou 466001, China; 3 College of Life Science and Agronomy, Zhoukou Normal University, Zhoukou 466001, China

**Keywords:** *Nilaparvata lugens*, sugar transporters, phylogenetic analysis, expression profiling

## Abstract

**Simple Summary:**

The brown planthopper, *Nilaparvata lugens*, is a destructive insect pest that poses a serious threat to rice production. Because the brown planthopper is a phloem-sap-feeding insect and relies on sugars as their main source of carbon, sugar transporters are particularly important. We identified 34 sugar transporters in *N. lugens* (*NlSTs*) and inferred their possible function based on their expression patterns. We identified a number of *NlST* genes that are expressed at higher levels in the gut than in other tissues, such as *NlST2*, *3*, *4*, *7*, *20*, *27*, *28*, and *31*. This study provides a basis for further exploring the function of *NlST* genes in brown planthopper.

**Abstract:**

Sugar transporters play important roles in controlling carbohydrate transport and are responsible for mediating the movement of sugars into cells in numerous organisms. In insects, sugar transporters not only play a role in sugar transport but may also act as receptors for virus entry and the accumulation of plant defense compounds. The brown planthopper, *Nilaparvata lugens*, inflicts damage on rice plants by feeding on their phloem sap, which is rich in sugars. In the present study, we identified 34 sugar transporters in *N. lugens*, which were classified into three subfamilies based on phylogenetic analysis. The motif numbers varied from seven to eleven, and motifs 2, 3, and 4 were identified in the functional domains of all 34 NlST proteins. Chromosome 1 was found to possess the highest number of *NlST* genes, harboring 15. The gut, salivary glands, fat body, and ovary were the different tissues enriched with *NlST* gene expression. The expression levels of *NlST2*, *3*, *4*, *7*, *20*, *27*, *28*, and *31* were higher in the gut than in the other tissues. When expressed in a *Saccharomyces cerevisiae* hexose transporter deletion mutant (strain EBY.VW4000), only ApST4 (previously characterized) and NlST4, 28, and 31 were found to transport glucose and fructose, resulting in functional rescue of the yeast mutant. These results provide valuable data for further studies on sugar transporters in *N. lugens* and lay a foundation for finding potential targets to control *N. lugens*.

## 1. Introduction

Sugar primarily provides energy and carbon skeletons for multicellular organisms [1,2,3]. Phloem-feeding insects mainly feed on phloem sap that contains high concentrations of sucrose (a disaccharide sugar comprising glucose and fructose) [4,5]. However, the ingested sucrose cannot be directly absorbed through the gut epithelium and must first be hydrolyzed into glucose and fructose [6]. The transportation of glucose, fructose, and trehalose (the primary circulating sugar in insect blood or hemolymph) across the gut epithelium is facilitated by sugar transporters (STs) [7,8]. For example, in *Polypedilum vanderplanki*, TRET1 (a trehalose transporter gene) is responsible for the release of trehalose from the fat body and the incorporation of trehalose into other tissues that require a carbon source, thereby regulating trehalose levels in the hemolymph [9,10]. The enhanced gut expression of ApST4 and the transport specificity of its product are consistent with ApST4 functioning as a gut glucose/fructose transporter in *Acyrthosiphon pisum* [11]. In addition, STs can remove monosaccharides (mainly glucose) from the gut lumen to cells, thereby regulating the high osmotic pressure caused by phloem sap entering the gut [12].

In addition to their basic transport functions, sugar transporters take the form of receptors on the surface of the cell membrane and may act as receptors for virus entry. These transporters are used for the transport of sugar molecules, which in turn makes them potential targets for viruses to enter host cells [13]. In the susceptible *Bombyx mori* race, there is constitutive expression of sugar transporter genes, causing the host to succumb to viral infection [14]. In *Laodelphax striatellus*, LsST6 can mediate viral entry into midgut epithelial cells and lead to successful transmission [15]. Additionally, sugar transporters have been demonstrated to be involved in plant–insect interactions. PaGTRs enable leaf beetles to accumulate plant defense compounds [16]. In a recent report, a cluster of Bombycidae-specific sugar transporter duplicates with complementary temporal expression synergistically facilitated the uptake of flavonoids, thus determining the development of the green cocoon [17]. There are eight families of sugar transporters present in plants [18], but the classification of sugar transporters in insects remain poorly understood.

Sequencing is a dedicated technology for genome analysis that enriches the information of genome sequences and provides insights into genome organization, genetic variation, and gene expression [19,20]. With the advent of whole-genome sequencing, more gene families have been identified in insects [21], including sugar transporters. A total of 19 sugar transporters (STs) were identified in the *Acyrthosiphon pisum* genome [11]. A genome-wide annotation of the silkworm *B. mori* revealed the existence of 100 putative sugar transporter (BmST) genes [14]. Moreover, 137 sugar transporters were identified based on an analysis of the genome and transcriptome of *Bemisia tabaci* MEAM1 [22].

The brown planthopper, *Nilaparvata lugens* Stål, is a monophagous rice herbivore and the most notorious pest of rice (*Oryza sativa*) [23,24]. It sucks the sap from the rice phloem using its stylet, causing direct damage. It is also a vector of viral diseases that cause secondary damage to rice [25,26]. The sugar in phloem sap is regarded as a major energy source in the brown planthopper [27]. Elucidating the mechanisms of sugar uptake in the gut and hemolymph is important for understanding the energy acquisition of plant-feeding insects and identifying new targets for controlling these pests. Several previous studies have reported on STs in the brown planthopper. In this species, NlHT1 (*N. lugens* hexose transporter 1) is likely to play an important role in glucose transport from the gut and in carbon nutrition in vivo [28]. In another study, 18 putative sugar transporter genes were identified from a brown planthopper EST (expressed sequence tags) database [29]. NlST6 is a facilitative glucose/fructose transporter that mediates sugar uptake from rice phloem sap in the *N. lugens* midgut [29]. *Nlst6* knockdown significantly affected oviposition development and decreased the fat body and ovarian protein content in the brown planthoppers. *Nlst6* plays an important role in *N*. *lugens* growth and fecundity, and it has potential as a novel target gene for the control of phloem-feeding pest insects [30].

Although several sugar transporters have been studied in *N. lugens*, genome-wide identification and systematic study of the STs family are still limited. In the current study, genome-wide identification and expression analyses of sugar transporters were conducted based on the genome and transcriptome of the brown planthopper. The transcriptomic data were used to mine for highly expressed *NlST* genes in the gut and to screen for candidate *NlST* genes responsible for transporting glucose and fructose. The overall goal was to increase our understanding of these STs and to identify those that might serve as targets for controlling the brown planthopper.

## 2. Materials and Methods

### 2.1. Brown Planthopper Rearing and Growth Conditions

The brown planthopper (*Nilaparvata lugens* Stål) biotype 1, which does not contain any *N. lugens* resistance gene, was originally obtained from the Center for Excellence in Molecular Plant Sciences in Shanghai, China. The *N. lugens* were reared on 1-month-old plants of the susceptible rice varieties Taichung Native 1 under controlled environmental conditions (26 °C ± 2 °C, 16 h light/8 h dark cycle) in Zhoukou, China. These insects were transferred to fresh rice seedlings every 14 days to ensure adequate nutrition.

### 2.2. De novo Identification of Sugar Transporters in Brown Planthopper

The current version of the genome of *N. lugens* (https://www.ncbi.nlm.nih.gov/datasets/taxonomy/108931/ (accessed on 9 September 2020)) was annotated through a homolog search and de novo predicting. The current version contains 18,160 protein models. Putative *N. lugens* sugar transporters matching the TIGRFAM sugar porter (SP) family motif (TIGR00879 [31]) were identified using the hmmsearch program, which is part of the HMMER package (version 3.0) [32]. All identified sugar porter family transporters had a hmmsearch sequence score of >237.80 (trusted cutoff) [11]. Subsequently, domain sequences with e-values greater than 1.2 × 10^−22^ were screened to construct species-specific sugar transport protein gene family domains of the brown planthopper. The family members retrieved were submitted to the SMART, CDD, and Pfam databases for further domain confirmation, and 34 sugar transporter gene family members were finally retained. Bioperl [33] was used to calculate the physicochemical property of NlSTs, including theoretical isoelectric point, instability index, aliphatic index, and grand average of hydropathicity.

### 2.3. Conservation Motif and Gene Structure Analysis of NlST Genes in N. lugens

The protein sequences of brown planthopper were downloaded from NCBI. For comparison, the protein sequences of sugar porter family transporters in whitefly (*Bemisia tabaci*) and pea aphid (*Acyrthosiphon pisum*) were used [11,22]. A phylogenetic tree was then constructed using the maximum likelihood (ML) method with 1000 guided replications in MEGA-CC 7.0 [34].

To search for conservative motifs, the online tool MEME (http://meme-suite.org/ (accessed on 24 August 2023)) was used with optimization parameters set to a maximum of 10 conserved domains in each gene, with a minimum and maximum width of each motif of 15–38 amino acid sequences. The gene structure of the *NlSTs* was predicted using the online tool GSDS 2.0 (http://gsds.gao-lab.org/ (accessed on 20 April 2021)) [35]. Finally, TBtools v. 2.019 was used to visualize the results of the conserved motif analysis, genome annotation, motif information, and the constructed evolutionary tree.

### 2.4. Chromosomal Localization Analysis of NlSTs in N. lugens

The chromosomal distribution information of the *NlSTs* was obtained from the reference *N. lugens* database, and the distribution of *NlSTs* on the chromosomes was visualized using TBtools and plotted on the chromosome location images using MapChart [36].

### 2.5. Expression Analysis of NlSTs in N. lugens

The RNA-seq raw data from different developmental stages (eggs, first, second, third, fourth, fifth, and adult; SRP310488) and various tissues (ovary, salivary glands, antenna, gut, head, and integument; SRP375431) were derived from NCBI [37]. The expression level of *NlST* genes was calculated as reads per kilobase exon model per million mapped reads (RPKM). The expression data were hierarchically clustered with average linkage and displayed in the Tutools platform (http://www.cloudtutu.com) [38].

The primers for all genes involved in the experiment were designed for RT-qPCR using Primer3 software [39] (Table S1). The spatial gene expression of the *NlSTs* were investigated using RT-qPCR as follows: ovary, salivary glands, antenna, gut, head, and integument were dissected from 3 d females of *N. lugens* using a stereomicroscope. Total RNA was isolated from each type of tissue using RNAiso Plus (Takara, Dalian, Liaoning, China, Cat no. 9108). First-strand cDNA was obtained from all samples through reverse transcription using the PrimeScript RT Reagent Kit with gDNA Eraser (Takara, Dalian, Liaoning, China, Cat no. RR047A) according to the manufacturer’s instructions, followed by amplification with qPCR using the Bio-Rad CFX-96 Real-Time PCR system with the iTaq Universal SYBR Green Supermix Kit (Bio-Rad, CA, USA, Cat no. 1725121). As an endogenous control to normalize expression levels with average threshold cycle numbers, a partial fragment of the *N. lugens* actin gene was amplified with primers qNlActin-F and qNlActin-R (Table S1). A relative quantitative method (2^−ΔΔCt^) was applied to evaluate the variation in expression among samples [40].

### 2.6. NlST Expression Constructs and Yeast Transformation

Full-length coding sequences for the gut-expressed sugar transporters *NlST2*, *3*, *4*, *7*, *20*, *27*, *28*, and *NlST31* were amplified from *N. lugens* cDNA using KOD Plus Neo polymerase (TOYOBO, Osaka, Japan, Cat no. KOD-201). The PCR primers used contained a 5′ BamHI site and a 3′ EcoRI site (primer sequences are shown in Table S1). The DNA was cloned into the pDRTXa vector using a ClonExpress II One Step Cloning Kit (Vazyme, Nanjing, China, Cat no. C112-01). *NlST* expression constructs were fully sequenced and used to transform *Saccharomyces cerevisiae* hexose transporter deletion mutant EBY.VW4000 [41] using the lithium acetate/PEG method [42]. Due to the deletion of at least 20 hexose transporters, EBY.VW4000 has very low hexose transport activity and is unable to grow on minimal medium plates containing hexose sugars as the sole carbon source [41]. Transformants were selected on synthetic complete (SC) media, pH 5.6 (0.67% yeast nitrogen base, 2% maltose, 1% agar supplemented with uracil drop-out mix), at 30 °C for 3–4 days. Positive transformants were replica-plated on SC media lacking maltose but containing 60 mM glucose, fructose, galactose, or mannose. The recovery of EBY.W4000 growth was assessed after 3 days at 30 °C.

## 3. Results

### 3.1. Identification and Related Information of the Sugar Transporter Gene Family in Nilaparvata lugens

A total of 34 genes encoding STs were identified through the sugar porter family motif analysis and homology search from the *N. lugens* genome and were named *NlST1-34* based on their location on the chromosome. Table 1 provides details of the gene ID number, genomic length, cDNA length, protein length, molecular weight (MW), isoelectric point (pI), and other properties of the identified NlST family proteins. The proteins encoded by the 34 genes showed different physicochemical properties, with protein length varying from 450 (NlST25) to 824 (NlST22) amino acids, a molecular size between 49.317 kDa and 91.969 kDa, and theoretical isoelectric points between 4.97 and 9.41. Sequence analyses revealed that the number of transmembrane domains ranged from seven to twelve, with most containing twelve.

### 3.2. Conserved Motifs and Exon–Intron Organization of Sugar Transporter Genes

**Figure 1:**
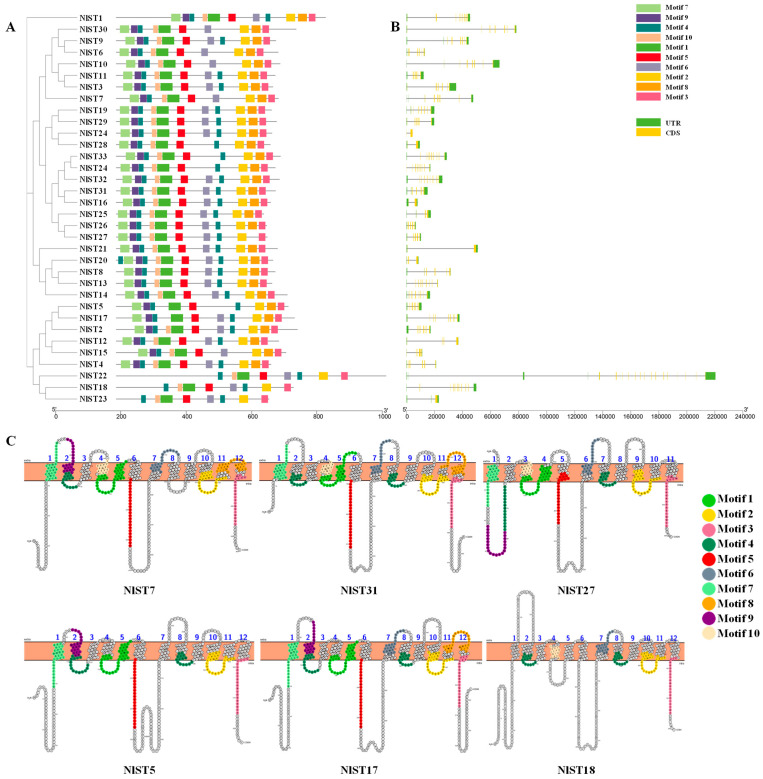
Analysis of motifs, exon–intron structure, and transmembrane helices (TMs) of *NlST* genes in *Nilaparvata lugens*. (**A**) Phylogenetic tree and motifs of *NlSTs*. Different colors represent different motifs. (**B**) Gene structures of *NlSTs*. Yellow boxes, green boxes, and lines represent exons, untranslated region (UTR), and introns, respectively. The lengths of the boxes and lines were scaled according to the gene length. (**C**) Lineup of conserved motifs on the TMs. The transmembrane structure is marked with blue numbers. The conserved motif is indicated in different colors.

To gain further insight into the structural characteristics of the *NlSTs* in *N. lugens*, we employed the MEME server to identify ten conserved motifs (Figure S1). We observed variations in the number of motifs, ranging from seven to eleven. Notably, motifs 2, 3, and 4 were identified within the functional domains of all 34 NlST proteins, suggesting their significance for these proteins in *N. lugens* (Figure 1A). Interestingly, motif 4 was present twice in most NlST proteins, except NlST6, NlST7, NlST10, NlST15, and NlST30, indicating that it plays a vital role (Figure 1A). Most members had all 11 motifs, such as NlST31. Some members lacked motifs, for example, NlST27 lacked motif 8, and NlST5 lacked motifs 6 and 1. NlST22, NlST18, and NlST23 only had eight motifs, lacking motifs 7, 8, and 9. We also analyzed the distribution of conserved motifs on the transmembrane helices in NlST7, NlST31, NlST27, NlST5, NlST17, and NlST22 (Figure 1C).

To better comprehend the structure of *NlSTs*, the exon and intron boundaries were analyzed. Exon–intron borders are very often conserved over long evolutionary distances [43], and structure determines function [44], so they play significant roles in the evolution of gene families. The results revealed that different *NlSTs* contained different exon numbers, ranging from 2 to 20 (Figure 1B). The gene *NlST22* contained the greatest number of exons (20), whereas *NlST16* and *NlST21* only contained two.

### 3.3. Phylogenic Analysis and Classification

**Figure 2:**
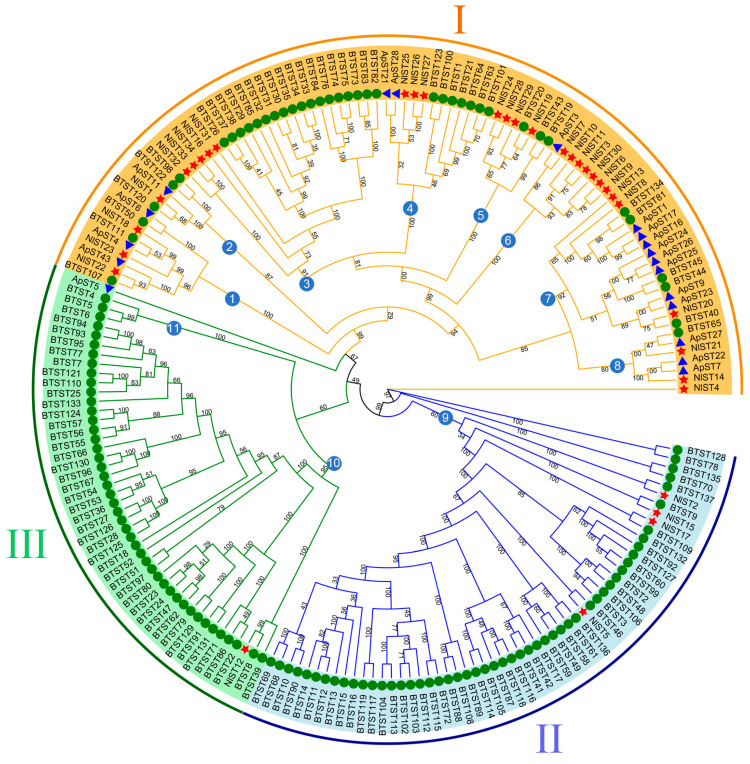
Phylogenetic tree of brown planthopper (*N. lugens*), whitefly (*Bemisia tabaci*), and pea aphid (*Acyrthosiphon pisum*) sugar transporter proteins. The picture represents the phylogenetic tree as constructed via the maximum likelihood method, where the numbers on the branch represent the bootstrap values. The red star, green circle, and blue triangle represent brown planthopper, whitefly, and pea aphid ST proteins, respectively. Major groups are marked in different colors, with each group marked outside the circle as I, II, and III. Subgroups are indicated by the numbers in the blue circles.

To characterize the evolutionary relationships of the ST gene family, a maximum likelihood tree was created (Figure 2). A phylogenetic tree was constructed by iqtree2 for the 34 NlST proteins, 137 *B. tabaci* sugar transporter proteins (BtSTs), and 18 *A. pisum* sugar transporter proteins (ApSTs) after performing multiple sequence alignments. Their protein sequence data are shown as Table S2. According to the topology of the ML phylogenetic tree, the sugar transporter genes of *N. lugens*, whitefly (*B. tabaci*), and pea aphid (*A. pisum*) could be divided into three major groups and eleven subgroups. Three major groups, namely, I, II, and III, contain 29, 4, and 1 NlST proteins, respectively. Group I marked in yellow is the largest subfamily, which contains 29 NlST proteins and all 18 of the ApST proteins. Most homologous genes from the same species are clustered in different subgroups.

### 3.4. Chromosomal Localization

**Figure 3:**
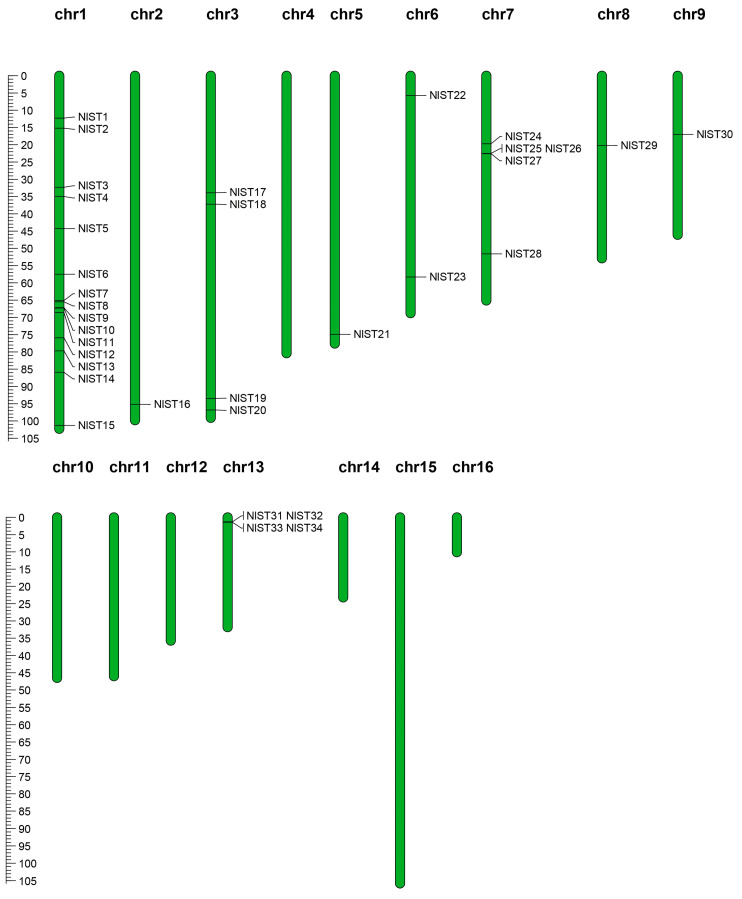
Chromosomal distribution of *NlST* genes in the *N. lugens* genome. The chromosomal position of each *NlST* gene was mapped according to the *N. lugens* genome. The chromosome number is indicated at the top of each chromosome. The scale bar on the left represents the length (Mb) of the *N. lugens* chromosomes.

The *NlST* genes are not distributed on all sixteen chromosomes and were found only on nine *N. lugens* chromosomes, presenting an unbalanced distribution (Figure 3). The number of *NlST* genes on the nine chromosomes ranged from one to eight, with the largest number of FtTH genes, which was eight on the first chromosome. Fifteen *NlST* genes were concentrated on chromosome 1, while a range from one to five genes was found on eight chromosomes (Table 1). Subsequently, gene cluster expansion events of *NlSTs* in the *N. lugens* genome were analyzed. In terms of the sequence similarity analysis and phylogenetic relationship, we identified three groups (e.g., *NlST9* and *NlST10* on chromosome 1; *NlST25*, *NlST26*, and *NlST27* on chromosome 7; *NlST31*, *NlST32*, *NlST33*, and *NlST34* on chromosome 13) of *NlST* genes. For example, the NlST31, NlST32, NlST33, and NlST34 protein sequences shared 63.25% similarity, whereas those of NlST25, NlST26, and NlST27 showed 59.08% similarity. 

### 3.5. Expression Profiles of N. lugens Sugar Transporter Genes

**Figure 4:**
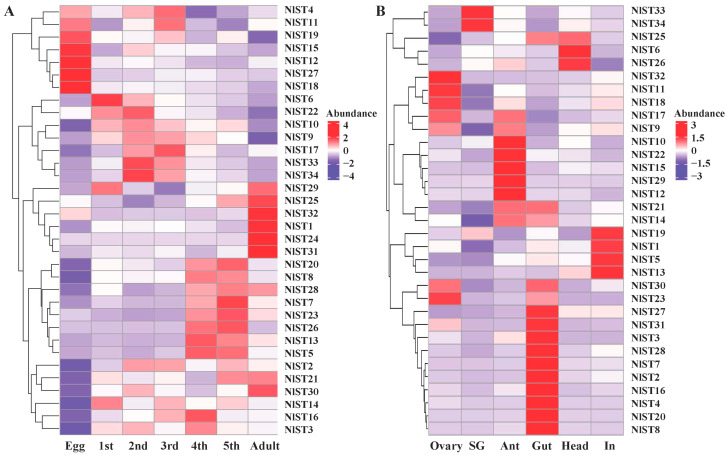
Gene expression of *NlSTs* across different developmental stages (**A**) and various tissues (**B**). Heat map and hierarchical cluster display differential expression profile of *NlST* genes across different developmental stages and tissues. Different tissues including ovary, salivary glands (SG), antenna (Ant), gut, head, and integument (In) were dissected from 3 d females for RNA-seq analysis. The expression levels are presented in the heatmap using fold-change values transformed to log2. Bright red, dark purple, and white indicate higher, lower, and equal expression values, respectively.

The expression patterns of *NlSTs* were investigated using RNAseq datasets obtained from different developmental stages of *N. lugens*. Expression analysis revealed that the *NlSTs* have a wide expression profile during all life stages, suggesting that they play important roles in development. The transcript expression of most *NlSTs* was the lowest in eggs, while the expression of *NlST4*, *NlST11*, *NlST19*, *NlST15*, *NlST12*, *NlST27*, and *NlST18* was high in eggs but low in other stages (Figure 4A). The expression levels of *NlST29*, *25*, *32*, *1*, *24*, and *31* were higher in the adult than other stages (Figure 4A).

The expression levels of the *NlST* genes were examined in the ovary, salivary glands, antenna, gut, head, and integument using transcriptome data obtained in a previous study [37]. The different *NlSTs* showed variable levels of expression in the tested tissues. The results revealed that the expression level of *NlST2*, *3*, *4*, *7*, *8*, *16*, *20*, *27*, *28*, and *31* was higher in the gut than other selected tissues (Figure 4B). *NlST33* and *NlST34* were highly expressed in the salivary glands; *NlST1*, *3*, and *19* were highly expressed in the fat body; and *NlST23*, *18*, *11*, and *32* were highly expressed in the ovary. *NlST24* showed a low level of expression in all tissues, and a distinctive expression pattern could not be determined (Figure 4B). 

**Figure 5:**
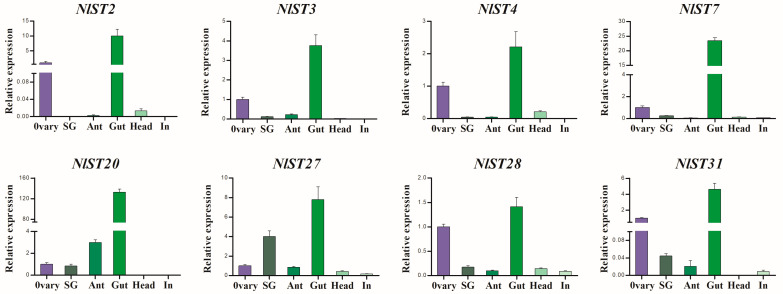
The expression of several *NlSTs* in different tissues of *N. lugens* by RT-qPCR. Different tissues including ovary, salivary glands (SG), antenna (Ant), gut, head, and integument (In) were dissected from 3 d females.

RT-qPCR was used to further determine the expression pattern of *NlSTs* in *N. lugens*. As shown in Figure 5, *NlST2*, *3*, *4*, *7*, *20*, *27*, *28*, and *31* had significantly higher expression levels in the gut than in other tissues. Interestingly, the expression of *NlST2*, *3*, *4*, *7*, *28*, and *31* genes in the ovary was second only to that in the gut, which indicates these genes may have a dual function.

### 3.6. N. lugens Gut-Expressed Sugar Transporters Transport Hexose Sugars

**Figure 6:**
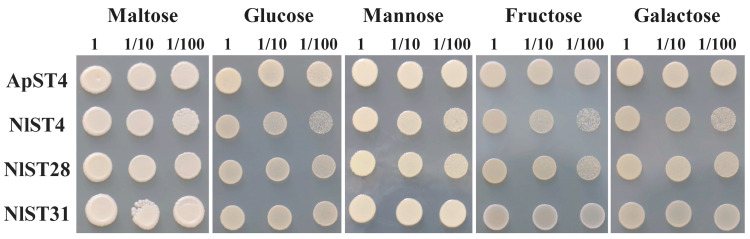
Functional identification of *Saccharomyces cerevisiae* hexose transport mutant EBY.VW4000 expressing ApST4 and NlSTs. Positive control cells (+) were transformed with ApST4. Yeast cell suspensions containing 10 μL of 1, 1/10, and 1/100 OD600 units were plated on minimal media containing 60 mM of the indicated sugar as the sole carbon source.

The utilization of ingested sugar as a nutrient resource and the maintenance of osmotic balance depend on the transit of sugar transporters through the gut epithelium and into cells throughout the insect [45]. All eight *N. lugens* gut-expressed sugar transporters (NlST2, 3, 4, 7, 20, 27, 28, and 31) were screened for hexose transport activity through functional complementation of the *S. cerevisiae* hexose-uptake-deficient strain EBY.VW4000 [41]. Yeast cells transformed with either *ApST4* (positive control) or *NlST* expression constructs produced recombinant transporter proteins. As shown in Figure 6, ApST4, NlST4, NlST28, and NlST31 were able to restore EBY.VW4000 growth on minimal media containing glucose, fructose, galactose, or mannose as the sole carbon source. These results showed that the transporters NlST4, NlST28, and NlST31 are functional hexose transporters that mediate the efficient transport of hexose sugars across the yeast plasma membrane.

## 4. Discussion

The whole-genome sequencing of *N. lugens* combined with a substantial amount of transcriptome sequencing data provided a wealth of invaluable information to explore the evolutionary mechanism, structure, and function of the *NlST* genes. In the current study, 34 sugar transporter genes were identified from the genome of *N. lugens*. The 34 *NlSTs* were located on nine chromosomes, with fifteen concentrated on chromosome 1, showing an unbalanced distribution. In *B. tabaci*, some *BTSTs* formed four different gene clusters that were located on four different scaffolds [22]. A similar phenomenon was observed in sugar transporter genes in *B. mori***,** and chromosome 27 had a maximum of 22 *BmST* genes distributed [14].

According to the evolutionary relationship inferred by phylogenetic analysis, these genes were classified into three main clades, consistent among brown planthopper (*N. lugens*), whitefly (*B. tabaci*), and pea aphid (*A. pisum*) [11,22]. According to the phylogenetic tree analysis, although the majority of NlST, BtST, and ApST orthologs can be divided into 11 distinct subgroups, most homologous genes from the same species are clustered in different subgroups. In addition, previous studies have found that homologous genes in the same branch can exhibit the same or similar biological functions [46]. Up to now, the biological functions of most NlSTs have remained unclear. However, the characterized function of STs in other species, such as *A. pisum*, can help to predict the gene functions in *N. lugens* via ortholog analysis in the same subfamily. For example, *ApST3*, the most abundantly expressed sugar transporter gene in *A. pisum*, is mainly involved in gut sugar transport [45]. The *N. lugens* ST genes *NlST7*, *NlST3*, *NlST30*, and *NlST6*, which are orthologous to *ApST3* and grouped in subgroup 6, are highly expressed in the gut and might be essential sugar transporters. Silencing *BTST40* and *BTST44* can significantly increase the mortality rate of *B. tabaci* at days 2 and 4. NlST20, BTST40, and BTST44 are grouped in subgroup 7, which indicates NlST20 may be used as a potential RNAi target for the bio-control of *N. lugens*.

We found that the complex expression of the *NlST* gene at different developmental stages may be due to differences in nutrient requirements and physiological behavior. Seven *NlST* genes were highly expressed in eggs. This indicates that they may play vital roles in egg development, because *N. lugens* does not feed while in the egg. Similar results have been found in *Bemisia tabaci* [22]. In *B. mori*, the expression levels of *St* genes remained at low levels in diapause eggs, whereas high gene expressions of trehalose transporter 1 (*Tret1*), *St4*, and *St3* were detected in developing eggs [47]. In *Harmonia axyridisa*, a lack of HaGlut4 can impair ovarian development and oocyte maturation and result in decreased fecundity [48]. Our results also found that some *NlST* genes were highly expressed in the gut, which is consistent with previous findings that *NlST* genes may be involved in carbohydrate incorporation from the gut cavity into the hemolymph. For instance, NlST16 was shown to facilitate glucose transport along gradients in *N. lugens* [49]. Simultaneous knockdown of the five sugar homeostasis genes in the potato psyllid gut yielded high mortality [50].

In addition, the insect gut is the first major barrier limiting virus acquisition; *L. striatellus* sugar transporters 6 (LsST6) mediates viral entry into midgut epithelial cells and leads to successful transmission by the insect vector [15]. Most of the *NlST* genes are expressed at lower levels in the salivary glands, except *NlST33* and *NlST34*, suggesting that these two may have a specific role in the pathway of sugar metabolism. In *Anopheles stephensi*, AsST in the salivary glands is strongly downregulated in response to blood feeding compared to sugar feeding [51]. In addition, we found that the *NlSTs* were differentially or highly expressed in antennae and ovary, suggesting that these genes may be involved in olfactory detection and breeding. However, we cannot exclude the possibility that these genes are involved in other physiological functions.

The hexose-deficient yeast EBY.VW4000 has been used in many studies to verify whether sugar transporters have hexose transport activity [52,53]. In this study, we used this method to determine whether some *N. lugens* sugar transporters have glucose and fructose transport activities, such as NlST4, 28, and 31. They are highly expressed in gut tissue and may be involved in moving glucose and fructose from high concentrations in the gut lumen to low concentrations in the hemolymph, such as ApST3 [45] and ApST4 [11]. Sugar transporters belong to the major facilitator superfamily (MFS) [45]. MFS transporters function as either facilitative transporters or secondary active transporters. Facilitator transporters facilitate passive solute transport across membranes by moving the solute along its concentration gradient without expending energy [54]. ApST4 is a facilitative hexose transporter, which is concentration dependent [11]. However, it is not clear whether NlST4, 28, and 31 in *N. lugens* depend on concentration. Therefore, further studies will include functional complementation assays and uptake experiments in EBY.VW4000 yeast cells.

RNAi played a major role in advances in insect biology [55]. Injection of dsRNA [56], feeding dsRNA [57,58], or spraying dsRNA [59] induces efficient RNAi in *N. lugens*. So, more precise targets in *N. lugens* need to be identified and characterized. In our study, *NlST4*, *28*, and *31* were expressed highly in the gut and may be the potential targets of RNAi. Our findings will help to improve the design of effective resistance management strategies to control *N. lugens* in agriculture.

## Figures and Tables

**Table 1:** Structural and biochemical information of the sugar transporter gene family in *Nilaparvata lugens*. TMD: transmembrane domains; MW: molecular weight; PI: theoretical isoelectric point; II: instability index; AI: aliphatic index; GRAVY: grand average of hydropathicity.

Gene ID	Locus	Chromosome Location	Strand	Genomic (bp)	cDNA (bp)	Amino Acids (aa)	TMD _num	MW (kDa)	PI	II	AI	GRAVY	Exon _num	Intron _num
*NlST1*	gene-LOC111043366	Ch1:12249555..12294808	reverse	45,254	1920	639	12	69.249	8.17	43.8	105.16	0.326	9	8
*NlST2*	gene-LOC111046157	Ch1:15214124..15231209	forward	17,086	1662	553	12	60.815	9.41	36.3	106.78	0.385	8	7
*NlST3*	gene-LOC111050771	Ch1:32315414..32350745	reverse	35,332	1437	478	11	52.160	5.27	37.29	111.57	0.63	6	5
*NlST4*	gene-LOC111051608	Ch1:34998301..35019321	reverse	21,021	1419	472	12	51.650	7.54	40.56	114.72	0.571	6	5
*NlST5*	gene-LOC111053007	Ch1:44305767..44316419	forward	10,653	1578	525	10	58.709	8.32	54.8	100.63	0.354	8	7
*NlST6*	gene-LOC111061715	Ch1:57476786..57489654	reverse	12,869	1485	494	11	54.629	5.77	46.31	113.7	0.653	7	6
*NlST7*	gene-LOC111047192	Ch1:65084966..65132472	reverse	47,507	1488	495	12	53.059	7.46	34.38	114.48	0.713	9	8
*NlST8*	gene-LOC111052473	Ch1:65520290..65551629	reverse	31,340	1458	485	12	52.959	8.02	40.24	104.56	0.672	7	6
*NlST9*	gene-LOC111043437	Ch1:67225255..67269476	reverse	44,222	1464	487	9	53.449	8.64	39.12	113.53	0.635	8	7
*NlST10*	gene-LOC111043448	Ch1:67312272..67378579	forward	66,308	1503	500	11	54.908	8.79	40.65	104.18	0.522	8	7
*NlST11*	gene-LOC111053755	Ch1:68579188..68591308	forward	12,121	1458	485	10	53.528	7.44	50.21	102.58	0.465	7	6
*NlST12*	gene-LOC111050667	Ch1:75849430..75886317	reverse	36,888	1491	496	9	54.976	8.7	42.61	104.56	0.495	4	3
*NlST13*	gene-LOC111053657	Ch1:79703392..79725619	forward	22,228	1428	475	12	51.968	6.04	29.17	113.49	0.544	9	8
*NlST14*	gene-LOC111044627	Ch1:85868992..85885680	reverse	16,689	1569	522	12	58.012	8.35	45.2	109.06	0.547	8	7
*NlST15*	gene-LOC111063213	Ch1:101274814..101285912	forward	11,099	1557	518	12	57.221	8.86	36.86	107.99	0.429	5	4
*NlST16*	gene-LOC111062486	Ch2:95229692..95237449	forward	7758	1416	471	10	52.011	8.9	37.92	107.62	0.53	2	1
*NlST17*	gene-LOC111047911	Ch3:33899719..33937443	forward	37,725	1635	544	12	60.404	8.5	39.21	99.17	0.276	8	7
*NlST18*	gene-LOC111044703	Ch3:37196798..37246547	forward	49,750	1626	541	10	59.221	8.7	37.7	120.37	0.567	11	10
*NlST19*	gene-LOC111058285	Ch3:93542486..93562265	reverse	19,780	1425	474	11	51.321	5.46	42.91	115	0.706	8	7
*NlST20*	gene-LOC111046366	Ch3:96787652..96796095	forward	8444	1437	478	12	51.654	8.76	30.02	112.41	0.581	3	2
*NlST21*	gene-LOC111058459	Ch5:74931747..74982525	reverse	50,779	1479	492	12	53.608	8.91	36.79	106.77	0.554	2	1
*NlST22*	gene-LOC111053621	Ch6:5695771..5916229	forward	220,459	2475	824	12	91.969	4.97	61.1	96.2	-0.071	20	19
*NlST23*	gene-LOC111063233	Ch6:58256698..58279641	forward	22,944	1401	466	12	50.999	8.34	38.51	113.03	0.622	3	2
*NlST24*	gene-LOC111052279	Ch7:19697049..19701246	reverse	4198	1428	475	11	53.039	6.53	41.19	105.89	0.598	2	1
*NlST25*	gene-LOC111057046	Ch7:22514752..22531997	forward	17,246	1353	450	10	49.317	8.7	34.39	105.31	0.599	4	3
*NlST26*	gene-LOC111057045	Ch7:22532940..22539385	forward	6446	1380	459	10	50.721	8.52	39.16	106.03	0.547	5	4
*NlST27*	gene-LOC111057042	Ch7:22548355..22558556	forward	10,202	1386	461	11	50.750	9.12	41.57	103.43	0.521	5	4
*NlST28*	gene-LOC111046465	Ch7:51608988..51618389	reverse	9402	1413	470	11	52.143	6.93	34.16	114.3	0.575	2	1
*NlST29*	gene-LOC111044534	Ch8:20188704..20208377	forward	19,674	1470	489	9	53.811	7.92	39.42	105.07	0.469	5	4
*NlST30*	gene-LOC120353060	Ch9:16961042..17039359	forward	78,318	1650	549	12	60.588	7.06	39.48	103.75	0.402	7	6
*NlST31*	gene-LOC111059089	Ch13:1252257..1267222	reverse	14,966	1461	486	12	54.105	9.2	37.94	106.48	0.423	7	6
*NlST32*	gene-LOC111059174	Ch13:1282389..1307879	reverse	25,491	1497	498	10	54.737	8.1	46.37	104.96	0.408	9	8
*NlST33*	gene-LOC120353968	Ch13:1402647..1431301	forward	28,655	1506	501	7	54.798	9.06	42.42	109.42	0.372	9	8
*NlST34*	gene-LOC111054748	Ch13:1437905..1454822	forward	16,918	1458	485	7	52.960	9.03	42.87	108.41	0.361	8	7

## Data Availability

The genome and transcriptome data of *Nilaparvata lugens* are available from NCBI under the BioProject numbers PRJNA913551 and PRJNA901050, respectively. The data presented in this study are available on request from the corresponding author.

## Supplementary Materials

The following supporting information can be downloaded at https://www.mdpi.com/article/10.3390/insects15070509/s1. Figure S1: Sequence logos for 10 motifs of sugar transporter domains using the MEME program. Table S1: List of primers used in this study. Table S2: Amino acid sequences of the NlSTs, BTSTs, and ApSTs.
