# Supplementary material for: Genome-Wide Identification and Expression Pattern of Sugar Transporter Genes in the Brown Planthopper, Nilaparvata lugens (Stål)
Source: Insects. 2024 Jul 7;15(7):509. doi: 10.3390/insects15070509 (PMC11277001; doi:10.3390/insects15070509)
Supplement: Supplementary file 1 [file insects-15-00509-s001.zip › Supplementary Table S1.pdf]

Table S1. List of primers used in this study

qNlActin-F	GATCACTGCCTTGGCTCCTA
qNlActin-R	GTACTCAGCCTTGGCAATCC
qNlST2-F	TGGCATCGTGAGATTGGTGT
qNlST2-R	ATCAAAGCTACCGGCAACCA
qNlST3-F	TGTATCGGAACTGGGTTGGC
qNlST3-R	AAGCAGGTTGACGTTGAGGT
qNlST4-F	CAAGGCATTGCACACTCGTC
qNlST4-R	TAAATCCGCTTGCTACCCCC
qNlST7-F	CTTTACCTCAGTTGCGGCCT
qNlST7-R	GACTGGAGGAGCGATTCCAC
qNlST20-F	CTGATGGCGTTCAATGCGG
qNlST20-R	CTTCCTGCCGAAAGTCTCAG
qNlST27-F	GATTCCCTACGCTGGTCTGG
qNlST27-R	AATGAGGCGATCGCGAAGAA
qNlST28-F	ACATTAAGTGGCTGCCTGCT
qNlST28-R	CCTAGGAAGGAGAGGACGGT
qNlST31-F	CGCCTCGTTTCTCAGCGATA
qNlST31-R	AGGCCACAAAAGGGATCCAG
NlST2-BamHI-F	TACGCGGACACTAGTGGATCCATGATGGGATCAAAGAATGGG
NlST2-EcoRI-R	GATGCTAGCCACGTGGAATTCGTCTTCAATGAACCTTCACT
NlST3-BamHI-F	TACGCGGACACTAGTGGATCCATGCTGGAAAAATCCATCCCC
NlST3-EcoRI-R	GATGCTAGCCACGTGGAATTTGATGCGTTCGAATTTGAATTTGG
NlST4-BamHI-F	TACGCGGACACTAGTGGATCCATGGCTGATGAACTTCAATCCAC
NlST4-EcoRI-R	GATGCTAGCCACGTGGAATTACCATTTGTTTTGGCGAAATCAT
NlST7-BamHI-F	TACGCGGACACTAGTGGATCCATGGCTTCGAAGGGCGATCACAAC
NlST7-EcoRI-R	GATGCTAGCCACGTGGAATTCTAGTTTCCTGTTGACAGGTCTCC
NlST20-BamHI-F	TACGCGGACACTAGTGGATCCATGGATAAGCCCAATAGTGCTATG
NlST20-EcoRI-R	GATGCTAGCCACGTGGAATTCTAGTTTATCATTATCATTGGGTAAG
NlST27-BamHI-F	TACGCGGACACTAGTGGATCCATGGCGGGCCTGTTTAGGCAGGTG
NlST27-EcoRI-R	GATGCTAGCCACGTGGAATTCTTCTGCTTGTTCAAAAGCTGTTG
NlST28-BamHI-F	TACGCGGACACTAGTGGATCCATGTTTTCAAGCTCAATCATTAG
NlST28-EcoRI-R	GATGCTAGCCACGTGGAATTACATTTGGGTGTAATCTCTATATC
NlST31-BamHI-F	TACGCGGACACTAGTGGATCCATGATTCAGAAAGGATTATTTAA
NlST31-EcoRI-R	GATGCTAGCCACGTGGAATTTCTCCTCTCATTTACAACACTAC
