# Supplementary material for: Genome-Wide Identification and Expression Pattern of Sugar Transporter Genes in the Brown Planthopper, Nilaparvata lugens (Stål)
Source: Insects. 2024 Jul 7;15(7):509. doi: 10.3390/insects15070509 (PMC11277001; doi:10.3390/insects15070509)
Supplement: Supplementary file 1 [file insects-15-00509-s001.zip › Supplementary Table S2.pdf]

Table S2. Amino acid sequences of the NlSTs, BTSTs, and ApSTs.

Genes	**Amino acids sequences**	**Amino acids numbers**
NlST1	MWYESEELKCNSKCLSSSQESLLEEQLEEASGGVAKICYAHHRLPPSALSSVTSSAASVASTATLLTDKH<br>TAAATLLTNKLLVNVHKHKLYNKATAKPNMKELNLLIASPTVGLDECRDKVPGQLTTTVVIPYSEME<br>RDDKHNIEYEKQPPQPHKQPPTALSLAAIMPQVLAAVSVSLGSMVVGFSSAYTSPAIASMNSNASSLH<br>VTPQEESWIGSLMPLCALFGGIAGGPLIETIGRRTTILSTAIPFILSFLLIASATNVATILAGRSISGFCVGIA<br>SLALPVYLGETVQPEVRGTLGLLPTTFGNSGILICFIAGKYLDWSLLAMLGAAIPVPFLLCMFLIPETPR<br>WFVEKGKQQRARKALQWLRGNNTDVSYEFSEIEKSNKDAEKCENESAFKELFSAKYSRPLIISIGLMFF<br>QQLSGINAVIFYTVSIFKDAGSTIDENLSTIIVGIVNMGSTFVATMLIDRLGRKILLYVSSTLMTITLLILGT<br>FFYVKNVMQIDTTEYGWVPLGSFVVFVIGFSIGFGPIPWLMLGEILPAKIRGTAAALATGFNWSCTFLV<br>TKSFSDLKAILGQHGAFWMFGVICLFGLVFVILLVPETQGKSLEDIERNLTGSGKDKVPVRTVRRMSSI<br>ANLKPLPSSI*	639
NlST2	MMGSKNGIHKNVDVESLKALVQPNNIKIKPIEDKSVLEGSVKLIQPAKRRKGSSFRQVISSFAANIGTIN<br>TGMTFGFSAVAIPQLEDLSSEIKIDKFQASWIASLSAVTTPIGCILSGYLMDLMGRKRTLLITQIPMIIGWL<br>IIAQATRVEEIYIGRLLVGLGCGMVGAPARVYTGEVTQPHLRGMLAAMASVGVSLGVTLEYMFGALY<br>SWKLVALLSSTVPTVAFICCFFLPETPSWLLSHGQVDKCRKSLVKLRGPTCDVEQELQDMVAYSNKNN<br>LAHSLTWKETIQALIHPSALKPFVILALYFVIYQFSGVNPVTFYAVEVFKDSGANMNKYLATVLLGIVRL<br>VFTVVACIVMRKCGRRPLTFVSSVLCGASMVGLGVYMYHFKSSAPWLPVALIFIFIAASTIGYLVVPWV<br>MIGEVYPTKVRGIIGGLTTCTAHFSIFLVVKTFPLIQDAISKPGTFCLYGVISLLGTIYFYIYLPETKGRTLQ<br>EIEDYFSGRTDTLKKPKITTVNNNKPVVLQTHKGDLLPEKKRHATFWYPASGYLFCTSEGSLKT*	553
NlST3	MLEKSIPISIPEKVVKPGLVNLYFAAFAVTLPFFSVGCCLSWTSPTLPALSDADWIKVDDEQGSWIGSLL<br>MLGATLGAFLSGQLLDSVGRKRTLLVDVLLLVLSWACLALARPLRSLEIIYLGRFISGIGTGVAFAAIPLY<br>VSEISDLNLRSALASMSEVFLAGGYLVEYCSGPFLGYSGLILVSSLMLFVSLVLFTRTPESPHFLVAKGRLE<br>EAVTELCWLRGNVPPQLVEDELKEIEMSMIVKRENNSGSLSDLVMDKANLRALLVCCGLSFFQQFSGI<br>NVMLAYAEPIFMKTSSSLSPAGSAVIIGTVQFLTACCTPIVVNRFGFKRLLMGSAIVMTLAQGALGLYFY<br>RDEHHLDVSELGWLPVSSATLYIVSYCLGFGPLVWAVMGEMYSPSIKEIGTSTSTCFNWFLAFLITKFFT<br>NISSFLGSYAAFWLFSCCCIFAFLFTFFVLPDTQGLSLKEIQDLLNGHKPNSNSNAS*	478
NlST4	MADELQSTISSNLKQVLVSLISSLGMLQTGMVLGYPAVMLPAIIDRGGNNSTFSVNSNQASWIASLSSIS<br>TPIGCLICGPMIDGWGRATTIKIINVPFILGWILIALYQELILIYIGRIICGIAIGLSSIPSAVYITEITSKSMRG<br>ALTIIGSLEISAGIIIIYLLGMFFNDDYQYLAWLSAGVSISSTLLTLCFLPESPIWLLNRKKKLQAKKTLME<br>DQHASDPSIARSEIETLAPSTSQENTSHTKWSRTIVQLRRPQSYKPLIIMNLFFLFQQFSGIMAVIVYAPN<br>FAKDAGITGHIQLVALSIAVVRMFSILIAAWASRKFGRRKPAIFSGIGMTIFLSMLTLNIYIQGIAHSSDTG<br>EIAWLPVTCLLLYIFTSCVGFATLPWSMIGEVFPTEIRGVASGFTACMAYVYSFLSIKFYPDMEEQLGEGG<br>IFLFYFIAATLGTVFVILFLPETQGKTLDEIGNDFAKTNG*	472
NlST5	MDLISGASINRAEDDDIESNKESEKSLTDGTTESSRGSSVSSFRRIIPQVIATTVQSLLLLALGMMVACPT<br>VIIAALHRPKSELPINDSQASWFAGILLICQPVGSILSGILQEWCGRKKCMLFVNIPQIVGWYLIYRAES<br>VDSLLVAASLLGMSVGFMEAPTLSYVGEIAQPHLRGTLASFTSTYVSAGFFVMYLLGTVATWRMTAL<br>MSCTVPVISFLCIMMIPDSPIWLLNKGRKLEAEKSLCWLRGWVTPQEIEREFSSMIRYCEDSKKAYNLQ<br>KATNNTPYTLVSSSPDVITSESRLNRFFRDLIRPEIMRPLRLVVSYFFFYHCSSLSGFRPYMVNTFTELNM<br>PINPHYMTIISAICQLTGGFCCMALVHKTGKRVLSLVSMSACVLACFSIGAYILLHKYQYVHQPWIPTIL<br>FICLFFCTNLGVSPIPWTLLAEVFPTRGRGIGGGLSAACYYIEFFLVSKTFLQLQSVLALYGVCFFYGGLG<br>VVGIIYLYFCLPETEGKRLDEVEKFFKSNNERNSTY*	525
NlST6	MAPIIGRKTNLYIAAFTANIAFSSCGCCLAWTSPTLPPLMAPESWLLVSVEQSSWVGSLIAVGGCVGPL<br>MAGRLLDLIGRKSSLLCNMLLLLVAWAVLMAAQHVWMLYLGRLLTGVAVGLIFMAVPLYIAEITEDE<br>DREALCALNELFLAAGFLTAYAAGSYLSYHNLIFVCIVMPVVFLLIFLWMPESPHYLLAKGKRQETIRIL<br>QWLRGGLPEDCIEKELIEIQALLDSSANQLTLRGICESRGGLRALYLTCALIFIQQFSGINAVQFYTQQIF<br>ARATEVLSPSLSCVLLGVVQAVSAVFTPPIVKYLGLKVPLIVSGAGVSVSHFMLGLYYYLDNCGYNVDS<br>IQWLPVLSLLSFTFFFCFGFGPLPWATMGEMFPPNMKAMSSAFVTSFCFMLMFVITKFFSNFSSMLGSH<br>SSFWLFSLLCALGTVFTYFYLPNTKGMSLQDIQDLLNDRYKTTSDPVDEKGKLYNIAPVCIEQNLENA<br>DHLHNTIHVLV*	494
NlST7	MASKGDHNSEAALPVHPLLGGDRPRGGARPTMESGKTSNRNLYIACCICNLASFAAGNALTWSSPTI<br>SKMKENNEIHISQESWLGSLIALGASLGPFVSGFLIDRIGRKKTLYLNAVLIILSWILIGIAISSFDSISFELIY<br>VGRVLAGVSAGSCYASIPMYIGEIAEDSVRGAVGSLLAFFLCGGFLLEYVVGPYVSYLVLILVSCIAPIAF<br>LVLFFFMPESPYYLIAQGRNAEAIRALQWLRGADDASIVQKEVTDMQNSVNESATQKSGAIELVKSKG<br>NFKALYLSCGLVAFQQFSGINVILFYSEQIFHLTGAALSPAICSIIIGAVLVISGGIAPPVTSIFGIKMMLIVS<br>GVGMFLSEALLGVYFFFKDKGVDVSSLSTAPIIFMVVYIVTYCLGFGPLPWAVMGEMFPPNMKAKASA<br>ITASFCWILGFIITLGFNSVAASLGMAFAFWIFSGFCVVAILFTVVLLPDTRGLSLQEIQDVLNGRPVNRK<br>L*	495

NlST8	MSTKATTVSAQTLVSTAVPAAKLPQYVAALIATIGGFCLGTVLGWTSPVLTSLSDYYGFEVNVDSQAWI<br>GSIMAIGAMVGCLPMSWMLDTFGRKSTIIILTVPTVAAWMMIIFAPSVTVICIARFILGFTTGAYAVAVPL<br>YTSEISENEIRGTLGTYFQLQLTIGITSAYILGSLLPIFWMTMVCGCIPVVLALAMLIIPETPTYYLKKFRV<br>DEARKALQWFRGSHYDVEPELMLLKANLDQMEAERVPFTQAFVTTPAKRGLVVGLGVMFFQQFSG<br>VNAVIFYAESIFKAAGSSMSPSLQTIIVGLIMVVMTWVATLAIDRAGRRPLLLISASIMAICTAILGVYFLL<br>LEKTPDFAKTIGSVPIVSLSIFIIVFSLGFGPIPWMFMSEIFPPQIKGPACSIACFFNWFSVFMVTKFFGDLQ<br>SKFGSYGTFWIFSGISIAGTFFVLNLVPETKGKSMEEIQKELGATPQMTPEDRMENGQKPAKF*	485
NlST9	MELQTPDHFATHKDAVEDQKKIRASRKNLYLAAISANLALSACGCCFAWTSPVIPKLKQPGSLIPLDE<br>FLGSWVGSLLMLGSAVGPFIAGIMIDAVGRKWTLLVDSVVLLVAWAILASAQSVWMLFVGRFMCGIA<br>VGIIFMGVPLYIAEIAEDKLRGALGSVIELFLSAGFMIEYCAGPFLSYNNLILVSVILPILFIITFIWMPESPH<br>YLLASGRRTDAAKSLRWLRGNISHDAVEKEITQIEAFLEESSEKKVSLRDLITNRGNLKALYVSVGLLSL<br>QQLSGINVIQFYVQPIFVKTGSSLEPKYSAMIVGGVQLISACFTAPLTRKLGFKIPLLISAAGTCVAQVLL<br>GIYFYMEEEKMDAVVYFGWVPIFSLVLYIFVFCSGLGPLPWAVMGEMFAPNMKALASAVITSFTFLLSF<br>FVTKFFANICIRLGTHFAFGIFGASCGVAFVFVYYCVPNTKGMSLQDIQDKLNKVKTPPEPTKYVTKL*	487
NlST10	MPGVGKANFSTFEVENNANIVEREKTKRTRLYYAALTANLAFFSCGASLSWTSPTLPRLTKGHEPWLP<br>PITSEIGSLIASFLMLGAATGPVVAGLLLNNVGRKKTLITSAALHLLSWIGLGFAPSLEVIYLCRFIQGVA<br>VAIAFTAVPMYVGEIAERKLRAVLASSSEVFLALGYMMEYAVGPYLSYVWLIVVSSIMPILTFVLFFFVPE<br>SPHFLLQAGNKNKAMKDLRWLRGNVSVTAAKREIAMIQASIEQGLHTGQRSTFHDILSSPGSVKAM<br>WLSCGLMFLQQFSGINVVLFYAQQIFDKAGNLPSEYCAMTVGAVQLISASFTPALVKRFGFKTPLAVS<br>ALGMTIAHAILGIFFFFEETGNDVSAVYWLPVTCLMLYIHVYCIGFGPLPWAFLGEIFPPNVKETASGC<br>VTSFSFFLAFIITKFYPNVDEKLGTFTAFWIFSGFCILAFNFVIFLMPNTRGLSLPEIQDLLNGRPITRLDKI<br>PEEPTVKNAGTNLI*	500
NlST11	MGSPNDGKDYDGFLRAKSNINLYLSALAANMAFFSSGCSLSWTSPSLPKLMAEDSWLPMTKEEGSW<br>AGSLLMLGGSLGPLISTNFQSRFGCKKALQGNSLILLLGWIVLGAANSKLEIYIGRFLQGIAVAVFFNLI<br>PMYMGEISEKNLRPAMATLSEVFVSGGYMLEYVIGPYVSYRTLALISGLLPLISFVLLLWIPDSPHYLLM<br>KGSSEEAARSFCWLRGFEKETRTEEFDELRNSLQEHENKQGSLIEIVKVPVNRRAFFIANGLLFFQQFS<br>GINVVLFYAQPIFMLTGAEFSSSVSAMIVGAVQTISACFTPLLANKFGFKRPLLASAIGMAVAQGLLGLY<br>FYCGLKGYDLSFYSFVPVSCLVLYILVYCLGFGPLPWAVMGEMFSPQVKSLASSISTSFCFFLAFLITKFFS<br>NLSDSIGTFAAFWLFSGFCLLALIFVICFVPNTRGMSLEEIQDLLNEKISLTRQRNSSTKGYLPLPQE*	485
NlST12	MFDSTLKRFWKIWEWGIGRTVAATIAAHTTSISVGMCQGYSAVLLPQLKEDANVNITTEEESWIASLG<br>VISTPGGALIAGLASELLGRKATVQLTSVPFLVGWATIALSTNKFALCVGRFVSGLAIGMASACYIYVAE<br>ISLPNHRGFLSAFGPIFVSLGVLVVYSLGYLLYWQEVAVLCAAVAVLSFVVMHFVPESPSWLASKELVSE<br>AKSSLTWLRRSPAMCDSELAGILQSLKVAKKQSEQGSSVCSLLKQCSSPSVWKPFFILTFFFIFQEGSGIYI<br>VLYYAVHFFQKIGSSLDSNVASILVATVRLFMSITGAICIQHFKRKTLAMISGMGMGLSMAICGLYSYLY<br>GHLPVEDRMYRWMPLLCVVVNVCFSMLGMLQLPWLMTGELFPLAMRGLLGGLVSSLAYLFIFGTIKI<br>YPGMEARMDQDKVMWIFSAFSFCVVIYVKLFLPETRGKSLHEIEMKFKREDYSVKNKNFDQEAQNK<br>TPNEVYIISRNDY*	496
NlST13	MVTEKNLETAAYGETIITEGRKFPQFLAAFFATIAALVVGISLAWTSPTFPIYKRENLLTTEQRGWISSLLS<br>IGALFGALTAGMIVDRFGRKLSLLLLGFPTLAAWALLSFSTSVDALYAARAIIGYCSGATSVAVNLYTSEI<br>AENSVRGKLGTFYQLQITVGILYTYIAGIADNVQIISIICGVTPIVFMVCFVWMPESPAYLVSKGRDEEAR<br>RVLRWLRGPDYQHEVELSLMKHSMEQQKKNQAGFMDVISDKVILKAFVLSLGMMVFQQLSGVNAV<br>IFYSGQIFESAGSSLSSQAASIVIGVVQVLATYCSTLLVERTGRRFLLLLSDSVMAICLIVLGGYFHYKEQ<br>NVDLSTWGWVPLVSLSLFIVVFSLGFGPIPWIIMGEIVPSNLKGISSSLGAGTSWILAFVVTKYFENLELA<br>FGSAGTFWLFAGICVVGTLFVYTLLPETKGKDIETILDELGGKKPELQLYDTKN*	475
NlST14	MGKAGYSAEEILEKVPLLRKTLTPTWPQHLAGISAGLMTMSMGFVAGWSAFATPYLRGKVAQLPFAP<br>VDTAQVSWIASIVTLGALLGCLPSGYVAGRLGRKVYLLSLTVPLVAGWMLIMLSEGRVSWIYLGRFMC<br>GLAFGGVTVTVPLYNNEIVEDRLRGKIGSYFDLMLSLGLFLVYVIGANTSYYLFSVICCTFPLAFVCLFV<br>WMPESPYFLISKGRIDEAKEAIRWLRRGITEEALEVEINLINCILQQSSSSHTNSVKSLDYAKQSLNKLK<br>NWNITSKAIVMAIGMMTFQRMSGVSAIMYYTVDIFHDANTSISPENATILVGLLGVIGCLISALLVDRI<br>GRRVLLIYSGFIMALGYTFMAAYFMAVHYGYQMDYQMRCIPLLAVCLVVLFFTIGLGPVPWFMIPELL<br>PEDVKGMALSATVAYKWVITFLVIKMFPVMVVEVGYAITYALMAAVCASGLLFFEHCIPETKNMSPFEI<br>HDEIAYYLNFGRHNVRSKYTEILNSPESEAKYTRNVTTVYG*	522
NlST15	MEGKSCVQIKSYADSIGSQNMSEKSELNGAVENGRNESKIATLPKQIAEGEPLLEETRKEKARLRLKQA<br>LAIGGPFIITLGVGMTSGFSAVLLPQLMSNETTIQITQDQSSWIASLAVPPMAVGCILSGYLMEEFGRRLI<br>QMFTSMLFVIAWVIVSVSTSVEVLYVGRIVSGLSAGLLSPLCLVYIAEVSDPVIRGTFLASIPLAISTGVLV<br>SHSLGTFLNWKLSAAICAVFPLVSYIFYYFAPESPLWLANKGYTKKAEETFRYLRGYSKSSSDELHILLN<br>RTTKAKDKSSEINKETVKRILSPTFLKPLITMLVLFFIQQFSGINAVIYYSVDILKSTTSNVNEYLATIIVDIL<br>RVIMSVTMCFVMKKYNRRSLALFSTIATGISLLLFSLAFRFLPQNYSWVNFVIISLYICCVYSGLVQIPWIL<br>TGEIFPTTMREIGSGGCTCFAFLMFFTVVKTGPVMFSEIGTGNGFLVYAVIALAGSVFIYFFLPETKNMT<br>LQKIGDGYKDDNDNKTVTKV*	518
NlST16	MASKGGGGVGGRGGGDAVLRQYIAVGTASLSFFIQGTSLGWPSPTIEKLKATTPITDAQISWMVSVLYF<br>GNFLSPIPAGYLMDRMGRKRMLVTLSILPILSWVLVIFSTDPLILYVSRFLGGLWAGISATVIPMYQAEVS	471

	QPQVRGALSTFIQIMTYIGVCYEYILGPFVSYTTLGLLNVAIPVMFVSAFMWMPESPYWYTINNRKEEA<br>LEALTWLRSGLPRSTIEDELQLIEHSVREEMKNKKTFSDLISTRGNRRGLLIVEMLAIFQRMSGISGVMA<br>FTSITIKPFTIFGILMTPDICVIIMGVVWIISTFISTALVDRSGRRPLLMTSSLGSGVAMTLIAIWFFLDRQTT<br>LNVSHTQFLPFLGLLIYGLFFSIGMGPIASTIQGETFPANTKAKASAITSISLAVTSFIMNKIYLDVENTIG<br>MYFNFILFASACFMCLGFVVFFVIETKGKTLHDIQEELNTKKSDLV*	
NlST17	MAADNHQSKPDKRLKDKMNEEIGKCNGNAELSPSNTSSKLRHPFRQALPQILATTAKNILLLGYGM<br>TLGFPTIVIPNLAQASNSTTDPYNLTLTRDQISWFSSINLICVPLGCFLSGVLTQPFGRKPSMMVLNVPFI<br>IAWLIYHYASSVNMLYAALVITGFSGGVLEAPVLTYVAEITTPQLRGMLSATASMIVILGVFIQFIFGTFLP<br>WRTIALVNVTFPILAIIALYGVPESPHWLMGKGRVEDAEKSLQWLRGWVKPHEVQVELSHLAKAIKSS<br>NFEESQRKRSWHAFKEKTFLRPYLLVSMTFLFGHFCGMTTLQTFAVSIFAEMGTPIDKYLATLILGLVQL<br>LGALTCVVLVHWTGKRPLAMVSLVGNSICWLLVAMYASWFRTHPQPHPHPEHSAAFSWLPMALIILS<br>AFLTHMCVRLLPWILIGEVYTPEVRATASGASGSAGYIFGFLANKSYFMIMDRIEASGTFTMYTIFSIGG<br>ALFLYYFLPETEGRTLVEIQEHFAGNRCLISNKETNSTGAQSKEDYAADNPAMDHVESHL*	544
NlST18	MERRDSPDSDVGYIEEDHPLRPQLAAQSIALLQVPPGQRSRHASVSSNLTDQDVPIYVRQKPNYTQPK<br>GLNPRLAFAIAAAAVGSAFQHGYNLGVVNAPQKLIEEWILGVIRNQTDASPPSDADQTKVTMIFSIAV<br>SIYCVGGMLGGAITGLVAEKYGRKGGLLFNNIFIVIAAALLGFSKAMNSYYMIIVGRFLLGINSGLNAG<br>LTPMYLSEIAPVQLRGAVGTVYQLVLTISILISQILGLNFILGTAELWPILLSLTIVPTIFQLITLPMCPESPK<br>YLLITKGQEIESQRAVTWFRGTIEVHDEMDEMRREYESMKLVPKVTLREMLVNSALRIPLFISLVVMIA<br>QQLSGINAVIFFSTSIFQLASLGDSAQLATLAMGAMNVLMTVISLVLVERVGRKVLLLVGFSGMFVITC<br>LLAVALAYVKSNKWLPYVCILLVIAFVVMFAVGPGSIPWFLVSELFNQSALPLATSLAVGTNWTANFFV<br>GLGFLPLQQLLGGHVFFIFAILQALFIVFIYKKVPETKNKTLEEISTMFKQISYT*	541
NlST19	MLAILTPSGRRQYAAAVCSSISVLMAGSTIGWITPILVGLLGPDSEVPMTADESSWVASVIEVGNFVTPL<br>PFGLLVDRWGRKPCLLSIAPMYIVSWLLVLATRSVLVLYFVRFVQGLAMGVVFTVLPMYLAEIAGADIR<br>GALSAFFQGMWYMGILTEYCVGPYVSYQTLAYVSLAFPLIFLLTFVWIPESPYYLLMKGEEEKAGVALA<br>WLRGESSPARVSEELQSMKMSVEKEMLRKGSWNDLVSTAANRKALLIVLIAALAEIMSGITAILTYASQ<br>TFGAADAKEGLTPDECTILMGLLVLMATVFAGGVVDVMGRKPLMLASALGCAVCELIAGVYYFLQE<br>KTSIDVTSYRWVAFSSITGYCVILSAGLGPVVATFQGELFPSNTRGLASAVVAISVTISSFFWMKLYQVIA<br>DHIGIYLNYFLFSLCCLVSSVFILFLVPETKGKTLAEIQSDLNSAHPPKPKKMAANEAVL*	474
NlST20	MDKPNSAMIIKQPTTNQEGRKITQYFVSIVATLMAFNAGTILAWTAPALPVLESPNSPLNRTISDSEAS<br>WIGSLAAVGALCGALPSGYVSETFGRKLPLLALGIPSVISWAIKLQGTSLEMLYAARLIGGFTAGAASGI<br>TPMYIGEIAESSVRGTLGTFFQLMLTVGILYVYVVGTLFSYSSLQVLCGIVPVVFMLLFVKAPDSPTYLLK<br>KGRRHDAEKALRLLRGPSYDIHAELNNIQYELDKASRQKVSFAKAIMKKASLKSLFIALGLMLFQQFS<br>GVNAVIFYSVSIFQAAGSTLDPSICTVIVGIIQVIVTYFSAVLVDKAGRRILLLISSSVMALCLGCLGYYFH<br>LQQKGEDVSNIGMIPLVSVCVFIVVFSLGFGPIPWLMTGELFSGDIKGFASSLAVTLNWTSTFILTKTFQS<br>FLTTIGADWTFWALASICSVGTVFVFLFVIETKGKSLEEIQCELAGKPYLPNDNDKL*	478
NlST21	MDTKAAKSAARRQYLATVIGNLSSLCIGMMLGWTSPVQPLLMSETPPVGNYSLTRTEVSWIGSMNFI<br>GGAAGTLFWGKVSDALGRKMAGCLIAFPFTLGWILMLVGTDFVWIVVARFVTGVGCSGAVINTPLYV<br>AEVSEDKRRGALGSCFMMFLNLGVVVSYTIGSFTSYHTFTSICLSVPIIYLSLFFWLPESPVSAFVRGKKD<br>VAETSLLWYRGNDSVAVAKEISRLQARADSIKNNRASFLSLFSSRGTIKALIIGIGFSFGQQFCGILAILTY<br>SVMLFKEAGTELSPFTGAIIVGLLQLCSSFVSSQLVDKAGRKVLLVTSYTVMSLTLLALGLYFDVKTYDR<br>FDLSEWSTIPIWAMSIHVIFYSIGAGPVPFVVISETFRPEIRGIAVAIVMFFSTTLSFASVRLFPIVVELVGLH<br>GCFWSYAASCILLTFFTVVLVPETKGRSLQAILKELEGEKQESDAEGADAELCTVKNIRIVKSNKTNSN<br>VR*	492
NlST22	MRVWRTPDDTLPPPPSHQEEDEEEEEDEEEEEEEDDDDDDEEEEERERKERVLDSGLDDYDEELTAPA<br>PPPVHPRPPRVAVPPPPHTPRLNHHLHRGDATQAEEDEATLRELLVSRVMLGGSLQRQVSVMSMNLS<br>AKLDELQRSDRSTGVQLETTVALCEIRSQLQQLTRSVESCQSEVSEVKRDMVAIRSELDTVQQVKEEIEE<br>LREYVDRLEEQTHRRKLRLLEQIIIKCCSIQLCEGLTFFLSYTILASMLGMLQFGYNTGVINAPEVNIENF<br>MKDVYKDRYGEDIHEESVKLLYSLAVSIFAIGGMLGGFSGGMIANKFGRKGGLLLNNVLGITGACLM<br>GFTKVSHSYEMLFVGRFIIGVNCGLNTSLVPMYISEIAPLNLRGGLGTVNQLAVTIGLLISQILGIEQILG<br>TDDGWPLLLGLAICPAILQLILLPVCPESPRYLLITKQWEEEARKALRRLRASNQVEEDIEEMRAEQRA<br>QQTEATISMTELICSPTLRSPLIIGIVMQLSQQLSGINAVLYYSTNLFMSSGLSEETAKFMTIGIGAIMVAM<br>TLVVMPLMDRMGRRTLHLYGLGGMFIFSIFITISFLIKEFFGYVQEMIDWMSYLSVVSTLSFVVFFALGP<br>GSIPWMITAELFSQGPRPAAMSIAVLVNWLANFLVGIGFPTMRNSLENYTFLPFSVFLAIFWIFTYKKVP<br>ETKNKTFEEILALFRHSNGRGSLRDSRLYGSMLNCPNALEPRITPSELAALMVAEEKPIPADSFTRSQER<br>RALLSSKHNPRRHSAYASIPTAAVGGAVAGACQRGSGSEPIRPPPPLPPPRAFPPNSAV*	824
NlST23	MNPDFSINLVFACMVAAIGAGFQHGYNTGVVNAPQNVIEKWMSDVSQERHGMPPDKNDITFLFSLA<br>VSIYCAGGIVGGLLTSTFAIHIGRRGGLFVNNLFALIAAAMMGLSKMAGSFELLIAGRCFSGLNSGLNS<br>GLAGMYLVEVSPRSMRGALGSMYQLIITISILVSQILGSQSIFGTDDLWPVLFGLTGIMALAQMLFLPCC<br>PETPKHIFNKGNKERAQKSLKWLRKREDVSAEMSEIQTEAEQEKSIGKASFQQFIQNPSLRKPLIIAIVI<br>MIAQQLSGINAVIYYSTQIFQKAGMSQQEAQLATMIMGTVNIIMTVISVFLVEIAGRKTLLLIGFGLMFI<br>VTALLAVLLEFIQYDFASYMCVALVVLFIVCFATGPGSIPWFLVAELFGQDARPLAASISIGCNWTANFL<br>VGLFFLPLQELIGPKVFIIFAVLQLIFTIFIFFKVPETKNKSLDEVLKYFK*	466

NlST24	MKVLKAFEQGNYALATFCDISSISVMVGATCIAWVAPILHILQGPDSPVPMTSDQTSWMVSFIEIGPFFT<br>PTISGILSDRIGRKPCIYFSVFLYLASWVLMLSVYTVPALYAARTLQGMALGITFTIVPMYLGEIAENHIR<br>GALTTLFQTLFHVGLLFEYCIGPYFSYETVLYVNAALSVLFFGLFYFQVESPYYYLIKNNEYKALLALTT<br>LRGNQPAVKIEQELREMKAVIEKEMSGPSKVSFSDVFKTRADRKALFLVVVITVTKAYCGLLAILSYATE<br>AFRVNDEGSVLGPNEYTILIGLQMVIVVGISAKFVDSLGRRPLILASSFGCFVCNVLVCVYFYMNQYSS<br>YDLTKYHWLIFSCFSLFAFFFSFGLGPVALTLQSELFPTNTRGIASSFCALSFTVASFLCLKLYQVFTDHY<br>GLYLNYLLFSIFCMIGFILTFIFLPETKGKSFSEIRLQLSGGEPTDVESSAAKS*	475
NlST25	MSVFRETAAAVSACICMFVTGCWLAWPSPALRKLEVQGGANVFIEGDEISWVVASLDVGNMVSPIPA<br>SYLVDRIGRKPVLLATGPLYLICWMLTFVPGTPYFLFLSRFLSGIGKGIAFSICPMYLGEIASVEVRGAISTI<br>STGALWGGSLLMFIVGPLVSYQWLNTIGAIFPIIFFMTFLWIPESPYGCLMRNKVEEARKSLQWLREGA<br>DQLAIEKELEQMKENVEEEMKTKGTFVDLVAIPSNRKATTIVMVSSAFQRLCGISAVLAFSSTTLPNVG<br>FQFFHVSQVIVVFGIVLTIGNFLATPLVDHLGRKPLLFASSIGLAISTATSGFYYLLRGDPEQAAWLPYM<br>ALVCFGIFHSIGLGVIPSTLLSELFPANVKSRAAAVSSIVFAAASFVTNKMYHPVQHSIGTHAMFFFFSM<br>NAVIFTIFNALFIFETKGKSFPDIQKRLKSLK*	450
NlST26	MRGVIRQAFCGSVGGLSLFITGCWLGWPSAFLRKLQRGECGFSLTLWQIGWSVALMDLANTVSPIPTG<br>YLMDYYGRKPAFMATAIVFQLSWLMVIFAEGPVLLYFSRIFAGIGKGMGFTILPMYIAEVAEKQIRGAL<br>STLFTLLLFGGTLFEFILGPSVSYMTLNIASTVFPLVFFILCFFLPESPYYLLSVGKKEQAWKSFCFIRKRNK<br>DEPVAGSDPVKEEMDAAEALVKKEMESKTDWMELLRTKGNRRSSLAVFTLAALQRLGGISSLLAYSST<br>TLPEEGGGFIGGPEQCVIYFGIFLTVGNFLSMSLLDILGRKPLLLVSNVIMAFVLYASGIYYILEATEDFDE<br>RPLKWIPYVSILVFAICYSLGIGVVPNTLLGEYFPANVKGKAAAAATIFFALASFSSNKVYPLISGKFGYQ<br>AMFLFFGTINAITVLVSILIVYETKNKTFAEIQEMLNS*	459
NlST27	MAGLFRQVFAGMAGGLCLLIVGCWLGWPSPWLRKLQQGEANFNLTKEERSWVVALMDFGNVLSPI<br>PTGYMMDACGRKPTFFATAVVFQVSWLLAVYANGPTLLYVARLLAGIGKGMGFTVVPMYLAEIAEVK<br>VRGALSAIFTFLLFSGTLLELVIGPYVSYNTLNVISSTIPVLFCVLVVLIPESPYFLLMKNRRQAAYRSFCW<br>LRNHYKGKQQAQSVGHDAVIETELEKMDAQVQKEMASRGDWSDLVGSRGTRRATLAVMSLCAMQ<br>RFGGISCMLAYTSTTLPETGGGPGLGPEAYMMIFGLVLVLANFICMPLIDWLGRKPLLIISTIVSTVVQA<br>ASAYFYYVRQVPDYDYSGLTWIPYAGLVLFAVAYSLGIGVVPNTLLGELFPANVKSKAAAVATIFFAIAS<br>FSVNKVYPSVPNYTMFAFFALTNFIATIFTWFFVIETKGKSFSEIQQLLNKQK*	461
NlST28	MFSSSIIRNQYVAAFSAALTMTICGSCFVWSTPLLPRLSGPEAIVQMTPTEISWMVSIVEVGSLASPFPTG<br>RIADRWGRKLALITAAPPVLISWCIILSVKSAYTFYIARLLQGYAVSVVFTVCPMYLSEIASVEARGRITA<br>LVQVMWYLGILLQFCTGSYLSYEVNAYTNLALTVLVFLFFLTQPESPHFLMLHNLEEKTIKALQPLRCN<br>ASEESIKKEVAQIKEALDAEKDNNACWADLIATEADRRVLLITQVLTAVRLLSGTITISAYLSELLILAD<br>WSTIDPKTCMVFFSTVTIVTIMIATCTVDKIGRIPLLIISSLGTCLSNGAIALFFWLKDNSDVDVTNIKWL<br>PAVVLTLFTFFFSLGLGPVTQTIQAELFPSHMRGYGSIVSVFNMTVLSFLGLRSYQIITDFFGVSANFIIFSL<br>VCGLGLIFTILYVPETKGKTFYEIRTHFQKHNKKEGNDIEITPKC*	470
NlST29	MFVFFKKSVFRQCLAAFGGSLTAMMAGTSYGWITPILIGLLGPKSEVPMTNDQSSWIISFIELGNLFSPIP<br>AGLLVDRWGRKPCLLITGPIYIASWVLVFTTRSVNVLYLVRILQGMGMGIVYTVLPMYLGEIASPEIRG<br>ALSTFLDAMCNTGILFEYCIGPFVSYPSLALISAGVPVVFLLIFPFMPESPYFLLMHNRREAAADSLKWL<br>RGKTDAADVEKELVAMKGSVDEEMKCKSRWRDLVATRVERRALLIAQVVTVSKSLSGIGAVVSYASQ<br>TFAKTADGDPTLLLSPDLHTIIMGLTIWLVTFVAAVAVDRCGRRPLLLISSTGCSLFMLATAAYYFVDEK<br>TDFDVDNYSILPFSTISAYCIIFSLGLGPLVPTLQAELFPSNTRGLASGLTSVTDTIASLICMKMYEVIADN<br>VGTFLNFWCFGIFCAIGTVAMYFIVPETKGKTFHEIQMDLSRKELPKEKLVMKNIGNTNKQPTNNIQM<br>YS*	489
NlST30	MSQQRQRSRLRLYLAAFSANLSFTSCGCAMAWTSPVLDNLDKSLGMEVSTSSKSWIGSLIAVGAICGP<br>LMANKLLDRVGRRWTLILDVMLLILSWVIVGLPGLRLTFVDPLSLLYFGRFLSGVAVGIIFMSLPVYIAEI<br>SDVESRGPLGSLNELFIAFGFFFEYVFGSVTTYLQLAMVSTAIPLLFLATFWWMPESPHFLVMRGRKAE<br>ALKAIKWIRNYADDEDAAAEKEAQEIQALLDDTKDQSSPCRDLVMVKGNRRALLISCGLIFAQQFTGI<br>NVVQFYTQSILEDSSKPQQASGLPTGVAPMLVGGTQFFSSILTPIATRLFGIKIPLLLSALGAAIGQGALG<br>IYFVLSDMEEGHGSVAFELIPVISMVFFMASFCIGLGPLPWAVMGEMFPPNVKALSSSVVSSFCFLLTFIL<br>TKFFKIVSDEYGRHSPIFFFAFCCFFGLIFIALTIPDTRGMTLQEIQDVLNGRTHRCQSPAVRRVTQTGSM<br>IQIIMRSGLLVEQPDHSKNLLASEEEQHPMIPMITDNKHELPAKHTAIYNSKAYKEATKV*	549
NlST31	MIQKGLFNNRVLRQYTAGFICSLSVMAIGCSIGWTSPAIPKLRSGSTRIQLSDVEITWVVAVTYIANIISP<br>VPSGWLMDRIGRKHTLIASNVLTIGSWFVLLYATCPLHLYIGRFMVGLMFGVGYTVVPVYLAEISEARV<br>RGSVSSLMSVMIYVGTNLEYCVGPYVSYDTLCMVSVTVPILFACTFAWIPESPYYFVIKGNTDAAKKSL<br>SWLRCDMGQRDMDAEFEKIKSTTELQMKDGGGKFKDLIATKGNRRALLIAEILAIIQRFSGIGPLIAYS<br>SITIPEKSIPGITRNEQMIVLGMTWLFTSIFASFLSDKLGRKVLLAISCTGCGIACLSASTWFYLREKTSTD<br>VTEVSWIPFVAFIFHALFYSLGLGPIALSIKGEMFPANVKAKASAVTTMVLAVNSFWLNKTYLIIADTFG<br>FYVNFLIYGVTMLLALIFIWFFVVETRRKTLQEIQERLEGGGKRKKVEEERVIEKVGESSVVNERR*	486
NlST32	MGCSTNKTEKADHLASILKQYLAASAALLAVLINGTFSGWATPCLPLLLSPDHSPLPVTLTKVDASW<br>MMSQLFIGQTLSPLPSGYLMDRLGRKPTLLYCAVLPLAAWVLIFFAQDVYALSAAMFLAGLFVGTVYT<br>VTPMYLGEIAEPSVRGRIITLTSVTTRLGIIFQVTIGVLLPFRLQAIVSLSVPLLFMLVFPLVPESPYYHLMR<br>RDRNKAESALKWLRGSGRSKANLEAELCELESAVDKQMRNKGDFRDLISTRGSRKALMTVETLAVL	498

	QRLSSMGPVMGYISTTLPLADFLQRLGPAACVVVIDLVRLCSGLVSSMLVDCLGRRALLITSGAFCGVI<br>MMCAGGWFYCDATGFLDVSAPHLRWVPFICLLLHGIGFSLGLGPVCSAVRSEFFPMNIKAKSSAVTST<br>ILAFASFLINKFYLTIGDSLGMHYNYWMYGVSCFAVCLFASCFMIETKGKSLQQIQDELNDDYTPHKEE<br>KETEYEEKSENNNVEMKV*	
NlST33	MVPLMKKLTCDLLDPLKKLKKKNVSFSLPADVGAATVLAQSVTLGMIAGWPTVSVPLLLAGHGPVS<br>TTPDQVSWLTTLHFLGNLLSPVPAGFLMDHWGRRTTLTVASILPVVAWPVVALCDTTTQLVVAWFVV<br>GMWSGVVYTVVPVYLGEIAKPQQRGLLGTLFSAARRLGAATEFVLGQVLTYRQLALISALPAILSALL<br>LLRAPESPYYLLRRRRDRQAAGEALKQLRPMHESHDLERELDDIERFVNERTKGGYRSLIATGTGRRA<br>LFVMEALAILQRLSDTGAVMGYIAASLPPRPLPFLEKQHAVMGLEAAKLFTGLISTLVIDLFTRKSLMLT<br>ASLACTAAMFWSGTWYLLVPKGTYNLHSTNYDLRTGTAWMPYGCLALHQAGYSLGLSPVFWSLRSE<br>LFPMAVKAQSSAITTTTLALVACVVNRMFFIIAQEFGIYLNYYIFCISCLLNALVTVFFVVETGGKTLSQI<br>QDDLDEAPSKDNEASADTRTQIPAV*	501
NlST34	MVPLMEKLTCDLLDPLKKLKKKNVSFSLPADVGAATVLAQSVTLGMIAGWPTVSVPLLLAGHGPVST<br>TPDQVSWLTTLHFLGNLLSPVPAGYLMDHWGRRTTLTVASILPVVAWPVVALCDTTTQLVVAWFVVG<br>MWSGVVYTVVPVYLGEIAKPQQRGLLGTLFSAARRLGAATEFVLGQVLTYRQLALISALPAILSALLLL<br>RAPESPYYLLRRRRDRQAAGEALKQLRPMHESHDLERELDDIERFVNERTKGGYRSLIATGTGRRALF<br>VMEALAILQRLSDTGAVMGYIAASLPPRPLPFLEKQHAVMGLEAAKLFTGLISTLVIDLFTRKSLMLTA<br>SLACTAAMFWSGTWYLLVPTGTYNLHSTNYDLRTGTAWMPYGCLALHQAGYSLGLSPVFWSLRSEL<br>FPMAVKAQSSAITTTTLALVACVVNRMFFIIAQEFGIYLNYYIFCISCLLNALVTVFFVVETGGKTLSQIQ<br>DDLDEAPSKDNDASADTRTQIPAV*	501
BTST1	MPRCIPRHIFNQIVSSITAFLTLLLSGIWLGWMSAVLPKFRGGEIPIPMTTDDLTWTVALMDFGNLLSPI<br>PTGYLMDRYGRLLTLRLAMLAFVAASALVLAASAPYHLFLARLLAGVGKGVGFTAATLYVAEIAGAK<br>IRGALSGVFIVMLMGGTVVSMTVGPYVSFTTLNVITAVCPVVGLFLTLFIVESPYYHLIRDDPAAAGEAF<br>ARVRDQSKGAANEAEFALVKRKVAEDMSGQKSILNLFTEKGNRRGLIIILVQGFLQRSGGISCILAYAST<br>TLPSDSFWQGKISVMVFSWIMVVFGLVALSLVDRFGRKPLHLISCVGLTLVTGVSAVYYYFYQKTEVDV<br>SQFMFVPHVGVVLFGVFYPVGVGQIPHTLQSELFPTSVKGQASALMTMALAISSFIVNKVYFAVDRGL<br>GVYFMYLIFALSNFLSMVFTAFYVFETKGKTLEEIQHFLKK	455
BTST2	MVIGFPAILIPAVTNDDNADNLHLTMAQASWCASLSFIFQPVGGIMTGLCLQSLGCKAVMILLNIPHI<br>VCWLMTYYASSIYTLCFAQAFFGCVLGLIEVPGLRYVSEISEPSVRGIIISSTSFFVSVGYLIMIFIGSLTDW<br>RNAAAISASLPLLCIILLILIPESPMWLMSKGRSEDALRSLQWLRGWTSAQMVHEEFQRIQFYSKNKTQ<br>KFLKTDHSGDSRSGSGGLSYFTTSTFLKPLIKCCIIFAIFDFGGMISFRSYLVKILQDLHSPVSSKWSSLWV<br>ALFGILGNVGCMLFIKKVKKKPMLRISLLCCILCLIFLTMFLFGYMQVIADSAFHHWCPLIMIVALFFFY<br>NLGIHPIAWAFLGEILPYRGRGPATSFVVCSHNLFTFVSVKTFPNLTQWLGLEGALLVYAGVCLCGILFT<br>YFLPETEGKHLSDIEIEVACGGKTNEDGLKATPCGS	458
BTST3	MELHLDTCPDGRSQQCQSSSLRQTLAQVLAATGKSLIMLSIGMLIGFPTVLIPVLTSKDYKGDLHFNR<br>DQASWYGALTYIFQPVGSIASGALLQSFGCKKLMILINVPQIGCWLMIYFATSNFVLYVSSALVGLVIGL<br>MEAPTIRYISEISHPSLRGILTTYSVLFTSLGFLAVYSLGSLTDWQHVALISAAVPVICIIILFQIPETPMWL<br>MSKGRAAEALEALQWLRGWTTADMVEDEFAKLEYYAKKKSCKTLHGYGKSVDTKDEVKETPAENG<br>IDGVSGGGKVSVKPSVSFQVEVGFEKRGFADKLGDLTCKEMTVPLGKCIVLITLSCFSGLPLIRPYLVAIF<br>AQLNLPIQPNWTSVLVTFLGIAGNIGCMALVNRVGKKPLVICSSAASAVCLILLSIFLMQSTPEAMQDR<br>ANPNWWPLILFFVLFFSFNMGLQPLPWVYLSEILPYKGRGIATGIAASIFYIVIFFGVKMFSTMERELGL<br>QGTFLLYAAVCIAGIFFTYFILPETEGRFLSDIETDAEREITVISNKGNTVNA	539
BTST4	MSCSSGNAVYAALPKHGDPVEIENFDVERPSTKTEFIHSKRDILYQGFTSLVAFSHVIHAGINLSFSTVL<br>HPQLDITKEQASWIASLGAVGTPIGSISIGFIMDRIGRQKTLLATAAINILAWSTLCLSPAQVDIKMIYLA<br>RLLEGIASGMTSIAIVYVAETTDKHWRPLFLSMNSIALSGGILLTTTVGVVLAWRAFAVFCLGVSTLSAL<br>LSLTLPESPSWLAQHHPERAKQALARLVTDPRAFEIEWTSLEPKAPKLLKTRKRPSLDKKIIRPLAVVLI<br>LITLQQLVGIYPTIFYSLELFQKINKRAQQSTEFNSSEETTMIIPTTPHIVTTPSSAQLFNSSSFEKSTGYPL<br>NLDVFNTSALIDHQHNVSAFIRGTGNPHDTRAFIQRTSLPQDAKVVINGTKHELGDELGEIKSKFDLR<br>SGAVQALMGLGIIRFITSVSMTALSKHLGVRTLLMSSSLGVGLTAILFSAYQAGWCSRSSDAVSFAIVLA<br>FITFGSLGLLVIPWTLVGELLPFEFRGVGQGIVVAYAYVIMSVVVKSYPSVEDELGTSAVFGFFGAVAFLTI<br>AFIYYFVPETKGRTMDEIQVYFQKR	589
BTST5	MENKRSDEYTALKLKNFDNNVQIVKHEFPDSESSDTDHRFRDGLYQSFVSLGVLSLLVHPGICVTLSTI<br>LYPQLNITKYQNAWIASLLSIGMPIGSLFVGPMLDKFGRKRTCIFTAILTSITWAMIISLPEDFDLNLLYIIR<br>FISGIALGLATSVIVYVSEVTNKHWRPVYLATVSVFLASGVLVATTVGIMMPWRSFALFSFGVSLVNITLI<br>LLVPESPHWLVRFSPEQAKRALVKLNKNKQDFNEEWEALEARRSARASSTFRKARLLSREVRRPMLRL<br>AMVFTFQQLGGGYLVVVYALQMFQTVVQSATTTSNHAPTPAPTNVTLPLVQKHEFRLGDLDTLCFIC<br>VGVIRVGMSGLAVLLTKHVNSRPILISSALSSAFSAFAFAAVMSGVFGPVRDLLPLFFVLLFLLLQSYGM<br>LVIPWAQIGELIPLSYRAKGGGFMTAYAYILMFVAVKIYPYAVDWVGLASVFVFFGLVAALASVYVYLYI<br>PETHGKTFKEIEDFFK	504
BTST6	MEYDNECTSLRHEENSARIVKGAAEKNGAVTSDHTWRDGFYQGFVSLVALSMLIHPGVGMGFSTILH<br>PQIKHLVSDEQNSWIASLVAFGTPVGALSSGPLMDKFGRRSTCILTCGVAIASWSALVFMPPEFSLLLLYI<br>ARILSGVAGGLTSAGVVYVSEVTNKYWRSVFLGLASVLLSTGVLLVTSVGYWMHWKSFSVFCLAIAVL	533

	NLLLLLTIPESPHWLIRTRPEKAKRALMRLNKNIESFEEEWQSLDEQFRKKQKAQMEGTPRPSLSSRQV<br>YVPFLIMGVTFTMQQLCGVYPIIFYALEVFQAITGDPGEISPSANSTNPSTETTTLASLIGNSTLMATDGS<br>RQDLKVKSLIGVGLIRFVMSILAVSLSRTIGRRPLLLSSCAGSAISGFAFSLYFFGCFGQEVNDLVSMSLVL<br>VFLLFSSYGLLVIPWAQIGELIPSSHRAKGGSYLISYAYFLMFLVVKVFPFTMETFGIGGLFMFFSIILTLEG<br>FFVYFYMPETLGKTFLEIEKYFATGVDSNGKGARSKWPIDKV	
BTST7	MFPGLESVALVYLVELSVKEYRSLLLGATAAIYTVGILISNVVGGYLPWHLASGIFSLTAFGFGVVHFFA<br>PESPAWLYKSGRPDAAVRSLQALGRSPASVRAELQLLELSARTVSENVSVAVLLEPTVWKPLVIVSLLLV<br>FQAFTGGYQINSYSEDIVQRLGTKYDPLHVSNIMSVATAVTNCTLGVYCISYMRRRPATITLSILVTLASL<br>GAGIYELLLRGAPGPFDWLPIALLAANLSLGSVVTNISWILSGEVFPLRVRGSTTGAIFFVGWGSQSLAI<br>KLYYASLAALQVSGLCFAYAAGSLCTVPLAVFALPETHNKTLYEVEQSFKRRESKDAETPVQDESNL	348
BTST8	MKSALKELHRIWESGIGRTVAATVAAHLNSISVGMCQGYSAVLLPQLTSHASPLQVSNDEASWIASLG<br>VISNPVGALLSGVCMEIFGRRTAVQLTSLPFLIGWTIIALSQTLTTLCIGRFISGMAIGMASACYVYVAEIS<br>QPEHRGILSSTGPVFVSLGVLIVYSLGSLCSWQFVSAVCAAAAMLSFSAMQLVPESPYWLASKGMTKE<br>SHAALSWLRSSAHVEKDISELVNNSRDISPRVSTLKLISDRFNDPCVWKPFFILVGFFLFQEGSGIYIILYY<br>AVDFFRRAGSTVDHNVASIIVASLRFAMSIFGSLCIQNFGRRTLAVTSGILMALSIGAAGVYEHFFEDFA<br>PADRPYPWVPLACILTNVCASMLGLLQLPWLMIGELFPLKVRGIMGGVVSSLAYLFIFATVKIYPNLM<br>ANLQMSGSMFGFAIASLMVVVYALMFLPETRGKTLLEIEQRFCDIPKTNSTENLEKGFYINPAISVSTVC<br>AIVENIKK	496
BTST9	MYRRFKDITMSAKKELDLAVAADGDQRPKAAPNKEEKPLISSKAKFSPFFRQFLAASGPIIATLSSGMT<br>AGFSAVLLPQLKSPNSTLKIDHDQASWIASMAALPMALGCIFSGVLMERYGRRMTQLLLCVPFLLGW<br>VLLSLATTVWHLYVGRFLTGFSVGLLGPPSIVYIAETAEPRHRGALLATVTLAISVGILLSHVLGTFLYW<br>KVAAAVSVFFPCLSFGLFWICPETPSWLAIKGYTSEAEEAFHWLRGYSDQAQGELKVILSKKPASRSDS<br>EEGSFKRLAHSLRSIFSLSFLKPFFIMNVFFFVQQFSGVNAVAFYSVDIMKTVSGNVDEYLATIVIDVIRV<br>VMSLATCILLRQFGRRPLGLISLVGTTVSLLSLAAVLKTPFYKEYPSLSWLPTGLLASYICFISIGLVPLPW<br>VMTGEVFPAAHRELGSGATSFFGFFVFFVVVKSSPFFFSTLGMVGTFQLFGGITLLGTVFIFCFLPETKN<br>KTLEEIEDLFSKSKPKPSPESAEVV	513
BTST10	MHNQAEPSTSLDVPEQNVKDHFQYANATKSAWAQILASIMQNWLFIEIGLELVMPTVILGSLHNNPA<br>EPLNMNDDQASWFGSIPAFCHPIGSLMSGLLQDKFGRKGAMMLVNIPIFMGWMILYFAESIHALYIVS<br>VIMGLCTGLAEAPLHAYIGEIGEPRMRGTISTISTSCCSIGVWLMFLFGYLFDWRTVALVSSSCSIITFTFM<br>TQLPESPTWLTLRGRLDEAKKSLCWLRGWVSSAEVEPEFLSLVKYTVKSARLSQENSAYSSLPIKEGEPV<br>AKRGGFLKEQLKELTNKRTFRPLRLMFIVFIITDIAWVHGIKPYFVKELRMLESPIDPNLALMIFSGLFIM<br>GAMVNVAFLRRFGKRRIALFSHILAGICILSIGMYASFLQSLTQYPLRVWLPLMLWFIIKFLHGFSIITLP<br>WQLVCEVFPLSGRGTATGLAAAWTHIVMSVLTKTYLYMEAWLGFSGVMYLYGVCTMAGVVHHYFY<br>LPETEGKTLEQIETYFTKNHDKREKFSVGKLQRQRAQSGSPF	526
BTST11	MGDSPKSSEREKLIKRYGYSSRSDFAQISATLIQGFILINHGLFMAAPTLILGALYEHPEDELYMDDDEA<br>SWFGSIPYICTPLANLASGLLQEMLGRRGSGLLSTMPMFATWILLYSANSVTTLYAVAAMMGLSVGLSE<br>APLNSYLGEISEPHIRGTLVTIASSAISVGIIVLYALGSFYDWRTTASIISVVPVITFILMTQIPESPAWLIGR<br>NRLEDAKKSLSWLRGWTSPNEVEEEFADLVSYTRHHVEDDEGALAKVAKMNGLLMTQVNAVFSKK<br>VLRPLRLVLISFAITFLAAISGMRPYSINELNAMNCPIDPKLILMVAQVLFIGGAVVNVSFLQRLGKRKI<br>ALFSYGVAAVSIIGIGAYCSFYRDLSSYSSAVWLPPSLLLVINFLGGLSILVLPWQLMSEVFPPAGRGLAT<br>GISAAWTHLVVSALIKSYLSIKSRVGMDGVMYLYGGATALGCVYFYFCLPETEGKTLEQIEACFVDQPD<br>TEEMLSVGASPVRHPENTRLRGNRKERGYGSTARSEL	525
BTST12	MSDPSPASGGPSDEKSSLTTKRQYGYSSRSTYAQALATIIQGWILIDHGLMMGASTLILGALHGNPNEE<br>LNMDDAQASWFGSLPYFCTPLANFASGFVQEALGRRGSGMLVNIPIFAAWILLSFADSIATLYVVAIIM<br>GVCIGLSEAPLNSYLGEIGEPHIRGTLITMTSTAISTGMTIMYGLGVFFDWRTTALISSIFPVLTFALMSQI<br>PESPAWLISHNRLDEAKKALCWLRGWVGPDEVEEEFQNLLMYAKKSAKEAKASEKTDDIDSKTDGL<br>LMTQLKALTNKNVLRPLRLVLISFTITFLAVVSGMRPYSINELNALNSPVDPKIILILFQILFILGAVAYGS<br>FLRDFGKRKIALFSNGIAAASIIGIGVFCSFFLDASEYPYLVWFPSILLLVINFLSGFGLYALPWQLMSEVF<br>PQAGRGLATGISAAWTHLVVSALIKSYLYIKAWVGLSGVMYLYGATTFLGFLYYYLYLPETEGKSLEQIE<br>SYFTDDPDLEEFFSVRRSPATDAEKIGLRSNKEDHSYGSTA	531
BTST13	MRDPPGPSVSLIDDAVDVDEDVPVPTNTRYEYSNRSTFAQVLATIVQSWLLIDNGLMKAVPTLILGSL<br>HDNPNEPLDMNDDQASWFGAIPYICTPITSFASGIFQEKFGRKGSMILVNIPIFVAWILLYVAESIAAFYT<br>VAVIMGLSIGLSEAPLSCYIGETSEPHLRGTLATIMSTAMIIGYFIMYTLGYFFDWRTAALISSAFPIVTFVL<br>MTPIPESSTWLIGRSRFKDAKKSLRWLRGWVTAEAVEEEYQSLLSNNRDPIRKKPSESTLQERGPEQED<br>YGFFRTQYQTLMNDNIMLPLRLVLITSFIGYVAVLRGMTPYLIGELNALSTPIDAKLVLIITQILFLLGAA<br>ADMMFLQGLGKRKIALFSHAIAAVCLLGIGFYAFHLQASAVYYPHLAWLPVIFLMVINFLGGFSLQVL<br>PWQLMCEVFPRVGRGLATGISAAWTHLVISLLIKSYLYIKAWIGLGGVMYLYGTITAFGVVYFYLHLPE<br>TEGKSLKQIETYFTSNHDRKEKFEVEK	516
BTST14	MSDPSEHSKSLCTGKNPEKTSSRYGYSSRSAFAQVIATLIQSWLILENGLLFGAPTLILGALHGNSSAEG<br>LRMNDDEASWFGAIPSICTPLASFASGYLQDRFGRKGVALLANIPILATWLLLYTANSIPALYIGAGMM<br>GLSQGLAEAPIISYTGEISEPHLRGILSTITSTAVMIGMIIMFVLGYYFDWRTATLISAAFPMITIAVMTQIP<br>ESPTWLLGKNRLDDAKRSLCWLRGWISNEEIEEEFQNLVNYTRNSAKEDCSATSNGHVQDSCNVKSD	524

	GFLKTHYNILTRKNVLRPLRLVMLTAFFTFVAVLVGMRPYYINELKALNSPLDPKLLLIIGQFLFIGGAV<br>TNMAVLWMTGKRKLVLFSYLTAAISILGLAAYSSFLKDSATWVTWIPIVLLCVISFLSGLSILILPWQLSG<br>EVYPPVGRGLATGISAAWTHLVISALIKSYLYMKAWVGFSGVMYLHCGCVVIGFVYLYCNLPETEGKS<br>LEQIETYFTKNVSRREKFSISKATNKSKSLRGDQRL	
BTST15	MSDSQETISAHDEVPLVPRNTRYGYSSKSTYAQVSAVLIQSWLFIDLGLQMTMPTLVLGALHRNPDAA<br>PLDLDDDQASWFGSIPDLSLPLASLSSGLFQDTFGRKGSMMLVTIPLFSGWLLLYSARSITTLYAVAVIW<br>GLVGGLCEAPLMCYMGEIGEPHLRGTLSSISTLATLTGSFMMYTLGYFFDWRTAALVCSAFPAITFVIVT<br>QMPESPSWLIARNRLDDAKKALCWLRGWVEPHEVEEEFQDIVNYTRMSTGVETILDDSDQSIEGNSL<br>GKKDGYLKTQYKQMTDKKILRPLRLIFALFSICGVASVAGNRPYLIGELTELGVPINPKLVLIGALVCFT<br>LGAMGNVIFLRRFGKRNIALVSHFLGALCIFGIGAYCSYAKVFTPEPQLRWLPVILWFTLQFLAGLSIILL<br>PWQLVSEVFPLTGRGLASGIAGAWAHLSSSILVKSYLYTESLISLSGIMYLYGCGTVVGLIYLYLYLPETEG<br>KSLEQIETYFTDQHDRKEKFSIGRPSSFSHASP	521
BTST16	MTDTVVPSRGSDVQHGKSTSNHQTKSSYRSGFAQILVALIQSCLIFDHGLEMGIPILVIGALHRNSSEAL<br>NMNDDQASWFGSILNIVHPVASLTSGFFQEKLGRKGSMISTTIPLFGAWMTLYFAQSVYALYAVVLIFG<br>LCRGLTEAPLHAYTGEIGEPLLRGTMSTISVSAAIIGASVVFALNYFFNWRSVALICSAFPIVTFALLTQIP<br>ESPTWLISKNRLDDAMKSLCWLRGWVEPNKVETEFKNLVNYVRNSTEQNESSSNGSDQTKKDGFFA<br>KGTRYLATNYKIMTSRKVLRPLRLVFIVVVVSLTAFLAGIRPYFIREIQELKSPIDARLILVSSTGSLFIGAI<br>MNVAFLRRFGKRKIALFSHAVAACCIFGMGAYSSFLRDAGSYVQLRWIPIIFWLLLNVVCGLSISVLPW<br>QLICEVFPVAGRGLAVGISAAWAHVVMGLMVKFYIYTEAWLGFSWMMYLHGAGTLIGLTYYFFYFPE<br>TEGKSLEQIEQYFAGNYNREENNSIDRRMKY	517
BTST17	MSSPEKPVKIQPIYQGCETNKFRYKNASRSTLSQVVATLIQNWLLIDLGMQLVMPTIVLGAIHNNPAE<br>DLSMNDEQASWFGSILFFAHPIGSMVSGFLQEQFGRKGSLILVNIPIFAAWSTLYLAGSIYMLYFVSLAM<br>GLSVGFCEAPLHSYIGEVGEPHLRGTLSTVTSAACILGMLIMYIIGYLVHWRTAALISSAVPVITIIFMTQI<br>PESPTWLIMKDRLKDAQKSLSWLRGWVEPEEVQEEFQELLSYTKISPPPYQANDIEIEKYELVRTDENG<br>REVAKEKESYLQAKFRELTDKKLLRPLRMVFIVFVFCYASSLIAMRPYMVGVFNEFGFPMDSKLILILTS<br>AFFFVGSILNVVLLRRLGKRRLTLLCQGMASVSIVLLGVYCSFFDRTNRIPSLVWVPISLLVSISFFSGLSV<br>ALLPWQLLSEVFPLKGRGAAGGISAAWAYYVGAVMSKTYLYLERWIKLNGVLFFYGAISLIGFWYFLR<br>YLPETEGKSLEKIESYFTKNHDKKEKFSKPKRSKKPVSPL	529
BTST18	MMPAADGSAPRTSSKWFRTFLAVTGAIGIEFIAGTIEAQSAVLLPQLEGSKELPITKDQASWIASMGTL<br>LCPLTSILCGPLMDILGRRLVFKIYYSVSTIGYLIIAFAKEVWHLYIGRLCLAFSLGFTVANVIYLPEITTTS<br>QRSLVLATINPLFSLGLLFSYVVGGYLRWDVASLIHTLICGLGLFSVLFLPESPAWLVKEQRPEEARNVF<br>RWLGRNAAKIDGDISRLQTTGNGPVKRSIPLKQLRHATVWKPFLILITFHFLQTMTGIYNIMFYTVEFF<br>RDLGTAFDPVLVTIGFAFSRFVVCVTVGYYFTTKCPRRVATAVSGFGSGAAYLVAVAYEVWWKGDRRF<br>QWVPVAAVLVSPGSPVDYDRRGVPALRARVHGRRDILRRERVPVPVHQVPLRAGRGPGDARRDDLL<br>RGGVPRVGRLRAPRPPGDPGQDHAPDRARLHGQEAEAATGEEVPIRQGGGALVRAAQRGSSTARGS<br>ARDGGGVRCQTTINGMEMLHV	502
BTST19	MGVGTYRQLVAGITASISVMCLGIAMGWSSPILQKFATERPSPILPVPTEDQLSWMIAFMEFGNLFTPVI<br>TGILVDVIGRQKTLVLIGPLFALSWLIIYLSQTIYFLYVARVIQGLGCGVVYTAVPIYLGEISDPKVRGALS<br>NLFQGFMYIGLLYAYVLGPFYSYSNFTLFCMAIPLVYSISVLFVPETPYFLLMQNKDTLARKVLRELRDS<br>TDDINEEMRVMKESVEKEMEGAKNPDKNRKITNIKLFLISQFFGSCQIMTNMYAILTYSSMVFDKGGS<br>HWLTPDQYTIFLGVVTLVSTVPSSFLVDKLGRKPLLVTSAVTCGVLELLAGVYFLLRERNAIQGDDYG<br>WCVFVFVSGLSFCYSFGLGPIIPTIQCELFPTNARGLAFGLTILITSVTAFLNILQFQWFASSPKLGMSAN<br>FFFGCVMCLLVAEFTVAVMPETKGRTFAEIQLLFHKDEGVERVSVLEAGEGDGPGSRLNPEEREQDEG<br>AGVGS	492
BTST20	MLIPRCHLRQYYAAAIASLSAMMTGAAMGWPSPVLEHFCEGAHCEVRMTAGEASWVLSLIEIGNLFS<br>PIPCGYLVDMYGRQPCLFATGPLFLGSWTLIICSRSVGYLYLARLVQGLGMGIVYTVTPMYISEIAGADV<br>RGRLSILFVGLLNLGILLEYIVGPFVSYRTLGYISISVPILFIATSIWLPESPYFLLMNDKSKEAINALMWLR<br>SDYSKDRIFEELTLIKDEVEQEKGTKTAAKNTFGDIFSSAANRKAFLIVQIAACADVLSGMTAILAYASV<br>IFAAPHNTTMEAEDYPIMLGLIMLLAIFPAAYLVDMAGRRPLLIFSCLFSGLFELVAAFYFYAAMKLDC<br>DVARLKWIPLLAICAFSVAYSMGLGSLVPTLMGECFPSQIRGPASSMTSITLCAISFLVIKFFQVVNEEIGL<br>YFNFFIYGVSSIACSAVLWLVLPETKGKTLSEIQQDLKASCKPSKPSLPLYVNASVPKGVEAS	484
BTST21	MFVKLQRGVVNQIAAAFTAELAGYNFGVWRVWPSLSITELRAGSAGFAVSDDQLAWITSLLYLGFLLT<br>PFFCSYLVVRLGRRTILYLTSALHTVSWLLVVLAQNPYHLYIANFFGGLAGGVGMTMVPVYVSEISSVN<br>IRGALIGSFLVFINLGQVMMVNMGIWLSYQEVNLFGLGGASVAFVLQFVVLTESPFYLLASKREEKATE<br>AYKRFHASSGEDKIETEVSALKAAVEKDMEHKSSYMELLACKRAFIILVVESFFQNLGGANSMLAFGV<br>ISLPKTELFLTPHQTILICVISGTFFNCISSSIVDSVGRKPLLVISVGMCSVFTGGLCVYFFLVEELQVDSLE<br>YEYIPHLLLIGFIASYTSGFAISRSLIIGEFFPTNTRTHAGLTSTFCFATAGFVVTLSFLHVVRLIGLYFMFFL<br>FFLVNFTNFWFSLFFFVETKGKNLFEIQEYLKSL	455
BTST22	MTPRKPPVPSPNQFFISCENVQLMQTEWSDETTNNTSTNTDDIDRPPASIFKPVLATLASTACQFFLGA<br>MLGQSSTMLPQLKAEGSSIRITDEQATWIASMGVIGTPMSSILCGPLTDKLGRKRIILVFLFLSAVGHAL<br>LGYSSNLTEILIGRFLLGTAAGFGFPSLVYISEISTPKHRSLLLSSATISASLGLTYVYSVGGSMPWDRASLI<br>TASLSLLALVYACSIPESPAWLFQHQRRQETIESLKWLKGQHCNIEVELKLLEASCNHQKKRALALSQL	499

	TKPTVFKPFLVLSCLAFMQNGTGFYILLYYSVDFLREFKTTMDPQKVSIALALTRLISCTVASLFIKRLKR<br>RTVGIFSGLAMAGILGVIYLSLTAFREAVGSGPVPVLSLLAYIFACSLGVHPLPWLMIFELYPLSVRGLMC<br>GLSNSVCYLFTFLFLKLYYVMITNLQIHGTILLFLCSSAAFGLFSAFLLPETQGKTLLEIEQGFMSKKDRA<br>ARRSG	
BTST23	MEADKLPVSEGFIRPFLLSACIYPLHIVGGAIMGQSAGMLPQLLNKDSSIPIDMEQATWIASSTAIGTCI<br>SSAISGPLSDMFGRMRVVQMSYFLMALGHALMMAASSFTGLVVGRLTVGLGLGCDFASFIYVSETVPA<br>ALRGVLMALYTVMCSLGFIYIYVVGGYYHWTIATGINAITATSGLALSFFLYETPVWLVRQGRLKAARK<br>SLRQSGIAASNLEAKLKELQDAAENKSTETFSLGDLLGPTVWKPFSMVCIMAVLQNMAGFYIVISYSIQ<br>FMAEFHSGYSPVQVTVGIAVVRLIAMTLTSFWMRHARRRVIGSVSGFGSSACLLAVFAFLHFGHLAPV<br>LVENQWILIALFFAYIFTMTLGIYALPWTMPFEIFPMKVRGLMSGMTYVSQFIAMFVSVKLYNVLMDN<br>LHLQGMILMFAVGSALFGSFCVTWLVETHRRTLDEIEAEFAGKSKVYT	460
BTST24	MEAEPPASAPTGCLRPFFVAACLFPLHICVGSIYGQSAGMLPQLLEKGSSIPIDRDQATWIASAPTLGA<br>CLASAISGTLSDVFGRMRVVRLSFFCMALGYAVMVAAESFMLIVLGRFLAGAGIGCNFPAFVYVSETA<br>PPAYRGLFLSLNALMSSLGLVYIYSLGGYFPWVYAAGATCLMATAGLVLTFFLHDSPAWLVRNGKLEA<br>AQKSLRRIEENAANVEIKLKELQETAKNEPTSSFNLRIFIEPTVWKPFMQVLGMSVLQNIAGFYIVIYYT<br>VQFMSEFHSTIGPLEVTVLIAVVRLVGISVASAWMRHAGRKFIGAFSGFSTAVVLLAIYAILKLGDRVKF<br>LAENNWILIALFLLYVLTMTLGIFPMPWTMPYEMFPIKIRGMMCGVCFCAMHVVMFVSVKLYNVLL<br>DNLELDGMILLFAAGAALFGVFSASMLVETHRRTLDEIEAVFAGRPIVTPPQKQKT	467
BTST25	MAESGDRKESLSWRCWSRTLIACSGAMMAFVFSGVTEGQSAVLLPQLKDEASPIHLTPEEETWIASLG<br>IVLSPVSASLTGPITDAFGRKLGLVVYHIIMGIGFAVIAVAKEVWHFYVGRCICSFAIGLEVAAIVYLTETC<br>SKEQRGLLLSTISPAFTIGVVVAYVIGGFLPWNVASAIFAAGSFLCSLGQLFGVESPAWLYKRGHTEAST<br>RALRRLGRTQAGIRQELELFKLTVKEQSQKFHLRELLHPTVWKPFIIMTIFHLIHCATGVHHIVYYTIDF<br>INRLGTTYDPLTVSIAISVARTIATCTIGVYFTSYVKRRFATILSGTLMTILSVGAGVYVYVWRDTAVDRR<br>PFQWLPVACVIAYIVIGRVGVTPLPWLMSSEVFPLRVRGSMSGATFVIGTGSIFISIKMYEDLIAAFHIWG<br>LLFIFGTACFSAVLLAVFVLPETLNKTLYEIEQYFMPKKGKKSGEQVDSTSRGEVIDSSPFKAVVKNRFLR<br>HSLRTPCLSIIFQRFIWQINRY	515
BTST26	MFSATPGVRRQFAAAITCSIGCLIAGLMVGWPAPTLKKLRQPDSPVHLTPHEEAWVVNAMYYGTILS<br>PFPSGFLINLIGRKTTLLVLAVFPTLSWILVYFSTSATMLMVARLFSGFWLGGIQTVVPLYTGEISEPHVR<br>GIFGSFFQVSSFVGNNFSFIVAPYVTIQTMATICGFFPVLFIILFVFCPESPYYYAMKKNSKAAGKSLSWL<br>RGDEPIMKELQTIQTSVDKELKDDETFTQKLTSIATDPANRKGFIIVETLDVMQRLCGISCMKAFSSIILP<br>PKLGPLTTDHCTIIIGFVWMFSSLICTGLIDKAGRKPLLYASSLGIFVSMLWTGIWYYLNDNTDIDVSSFK<br>WLPLAGFLVFGVTFSFGLGPIVKIYQGEMFPNNLKGMASALTAIIAAFASSVSTGMFPILTETIGMYANF<br>LIFSTVGLVNFLFTYFYVIETKGKSLQEIQAAELNGERPVVTQSNGDSEKAV	473
BTST27	MATLPGKGEAPKGQELEKVAPEKWCRVLWACGGAMMIFFFSGVTEAHTAVLLPRLEEVDSPILIDAD<br>EKTWIASLGIVATPLSSVLCGPCVDYFGRKIMVQCYYLVCALGFALIASANSVYQIYAGRLICSLGIGFEV<br>AAIVYIAEVSTVRMRSVLLSLTYSVLYGGGTLFAYAVGLSLPWNLGSAVFALVCLILFGYESFVPESPSYY<br>YKKGDTKKAIVAFTQLGRTEDQIAQEIKILEERKTKTEQKVDWRTFIHPTVWKPFLIIAFFHCLQAFMG<br>LWDELYYTVDLVTELDSAYDPFEVSFILTLSRFLVASTAGVYFTTRVSRKLAAAASSFSMAVALLVVAVYE<br>KRYELTAKWERPYPLVPIVGLVGAVMASGAGMFFLPMLMSGEVFPLRVRGTMSGAVFFVGTGSMFLF<br>LKLHVFLVTTLGVPGIYTMWTTACFVAGFFAVFVLTETHGKELHEIEDSYRSKKHRSTDIERTKF	481
BTST28	MEFATEQEPKQTPSEKWGRMFWACGGAMMIFFFNGVAESHTAVLLPRLQEPDSPIHINPDQMTWIAS<br>LGIVGAPVSGVLCGPCVDYFGRKIVVQCYFIVCALGYALIGAASSVYEIYVGRLILSLGIGFEVAGIVYIA<br>EVSTARMRSVLLSLTYSVLYGGGTLFAYVVGLSLPWNLGSAVFALACVLLFGYESFTPESPPYLVKNGH<br>TDEAIAAFKRLGRSDDQIAQEIRILERKGEPRQQVEWRTFLEPTVWKPFLIISCFHFLQAVTGVWDTLY<br>YTVDLVTNLGTQYDPYEVSLFLTVGRSLMASTAGVYFTTRVSRKMAAAVSTFSMAVSLFILAVYEKMY<br>EFTSELERPYPLLPICALIGAVMASGAGFFFLPMLMSGEVFPLRVRGTMSGAVFFVGTGSMFLFLKLHV<br>FLVTTLGVWGFYAMWTAASFITGFYSIFVLTETHGRELHEIENSYRSKKQKGADIERTSQF	474
BTST29	MLTLTLGIRRQLAAAFACSLASLIAGCVLGWPSPTLKKLREPDSPLHLSTYQEAWVVNALYYGTVLSPF<br>PSGYLMNKLGRKMSLLVLCVFPTLSWILIYFSSSAYMLMLARLFAGFWTGGTQTVMPIYIAEISEPQVR<br>GVFGTFIQLNIYLGTNFAFLVGPYVSIQLMAILCGILPVIFFVLFGLCPESPYFYTMEGRHAAAADALTW<br>LRGDAPVDAELRTVRHSVEKESANQSGVFRRIADLVTVPANRKAFIIVETMNALQRFSGISCMMAFSS<br>VVLPETGALNSDHCTIIMGIVWMVSCLGTSGLIDKAGRKPLLYVSSIGIGVSMLWTGVWYYLDENTTY<br>DVTGWNWLPLAGFLAYGCTFSLGLGPLSSTYQGEMFPSNLKGQASAITTITTALASAISTGLFAVLSKN<br>VGVYMNFYIFSAVGFINFFFTYFYVIETKGKSLQMIQAELNGEEIIKPEMKRLGKSVKK	472
BTST30	MYTFACSGIQRQLAVAFTAAFSQFLIGFLMGWPAPTLKILRHPSSEVHLTPSEEAWVVNAMYVTSFLSP<br>LPSGVLMDTIGRKTTMVVLCLFPIISWILIFYQQTGLMLLIARAFAGVFVGGVQMLSPVYAGEIAEPRVR<br>GIAGALIMVHGFAGAISVYIIGPYVSIRTMAVIGGAFPIIFLLLFTLCPESPYYYIMRGRQKSAEEALTWL<br>RGGAPVKQELDIIQTAIEKETQSGKGYFTKMLSLVTVPGNRKAFFIVEVMNFMQRFSGLSCLTVFSTIVL<br>PERVGPVTSDHGTLLMGVCCLLASLGCIALIDKAGRKPLLYFSSIGIFFSMLPTAFWYYLDRETSTNVKE<br>VNWIPYAGFLSFAATLSLGLGTIAPAYKGEMFPSDLKGQACALTSIIVGIASALGTALFPVLTSHVGLYA<br>NFLLFAAMGLVNLIFTYFCVIETKGKTLQMIQAELNGETLEKI	463
BTST31	MYLFKLNLGVERQLAAAFVASISIFTVGMLLGWPAPTLKLLRQPDSPLHLTPSEEALIVNALFFGTFLA	423

	PFPCGALMDHVGRKTSMLTLALFPILSWAVNGFAGMIFVYIVGPFVSLPTMAMIGGIFPVTFLVLFLHC<br>PESPYYYIKRGLHADAGKALSWLRGGASIETELASIRTSIEKEAKADRGLVKMLRLITNPANRRAFIIVQ<br>GIACLHRSTGIPCIVAFSTVLLPSHIGALTNDNCTIIMGVALLSASLCCSAIVDTVGRKPLLYLSSIGMFTS<br>MLPTAVWYYLDRETSTDVSGVNWIPLAGLLCFAVTFSVGLGPIMQIYAGEMFPTDLKGHACALATMN<br>QAVSAVIVTQIFVALTVYVGLYANFVLFAAMALVNLGFTYFCVIETKGKTLQMIQAELNDFVQPLLLTR<br>STVRRSQ	
BTST32	MTSFIYGTLVGWSAPTLKKLREPDSPIHLTPGEEVQMINAIYAGTLLGTFPCGALMNRVGRKGSLLLLS<br>AFPITSWSAIYFARTASTLLIARFFAGVWGAAAMTIRPIYVAEIAEPRVRGAAGAFTMVAMFAGTIFVF<br>VVGPCVSIQTMAVINGVAPPVFFLLFSLCPESPYYYIMRGRHADAAKTLAWLRGGAPIESELTSIQTSIE<br>REAKAGQGYFKKMLSLVTVPANRKAFFIVEVMNFLQRVCGLSCMAAYSTVVLPQRVGPFTADHCTLII<br>GIVWFLSSLGCSTLVDKLGRKPLLYVSSIGILASMLPTSAWYYLDKETATDVTWINWVPLAGFLLFGVT<br>VNVGLGSIAPTYMGEMFPSNLKAEASALTIMAVSVSSGVSIAVFALLTVHVGLYANFLVFAAVGVVNW<br>VFTYFCVIETKGKSLQLIQDELHGGTRWKPPKNNHSDGKLTV	455
BTST33	MLNFIVVSGIERQLAAALISGFSCFIAGALTGWPAPTLKKLREPDSAIRLTPSEEAWVVNALHITTILSTL<br>PLGSLMNTLGRKTTMLVLCVSPIVSWVLVYFARTSFVFIVARSIGGLWLGGCQTLLPIYIAEIAEPRVRGI<br>AGSFIMVNAFAGIIFVFTIGPYVSVPLMAVINAACPCVFFLLFLFCPESPYFYVMRGRYEAAGRALTWL<br>RGGAPIDGELNIIQTSIENEAKEGQGFYKRMLLLITNPANRKAFIIVEVMTFLQRFSGLSVLNSFSTVILP<br>ERTGLLTADHCTLIMGIVWLLASMCCSALIDKLGRKPLLHISSIGIFASMIPTAVWYYLDRETSTDVTRV<br>NWVPFAGFLVFGFTVSIGIGPIAAMYPGEMFPSHLKAQASALSNMVSSISATLSTVMFVTINAHIGLYA<br>NFLVFAAVGIVNLVFTCFCVIETKGKSLQMIQEKLKHGTWKESGVNRETPETH	474
BTST34	MLNFITGGIQRQLAASLIAGFSCFIGGSLMGWPAPTLKKFREPNSAVRMSPSEEAWMVNALYIMSMLC<br>TLPIGAVMNRIGRRTTMLVLCASPTISWIMIYFARTSFVLIAARAIAGFWLGGCLTVLPIYIAEIAEPRVR<br>GIAGCFMMLNAMVGMLSAFAIGPLLSVLTVAVINLVYPILFFALFLFCPESPYFYAMRGRHAAAGRAL<br>AWLRGGAPIEGELTIIQTSIEDEAKAGQGYVTRMLLFITNAANRKAFFIVEVMNFLQRFSGLSALAAFS<br>TVILPNRIGPLTADHCTFFMGVNWLVASVCCTCLIDKVGRKPLLYLSGIGIFASMFPTAVWYYLDRETST<br>DVTRVNWVPFAGFLLFGFTMDIGLGCIAPIYTGEMFPSNLKAQAAALSNMVASISSTLSTALFVVISEK<br>VGLYANFLVFASVGIVVFLFTYFCVIETKGKSLQMIQEELHCKSRKKSETDREKSVTS	473
BTST35	MLTLTFSGTQRQLLAAFISTISLFMLGSMMGWPAPTLKLLREPDSSLHLTPSEEAWVVNALYFTTILSPL<br>PSGALMNAIGRKATMLALCVFPTASWALIYFGRTASVLLAARVLAGFWVGGCQTIMPIYIGEIAEPRV<br>RGIAGTSIMVNAFLGTIFVFIVGPYVSVPTMAVMNGVIPPVFFLLFSFCPESPYYYVMRGRHADAARTL<br>AWLRGGAPIESELTSIQTSIQTCIEREARAGQGYFNKMLSIVTVPANRKAFFIVEFMNFLQSAFSTVILPA<br>HAGPLTADQCTLLLGAAWLISSLCCSALIDRLGRKPLLYFSSLGILVSMLPTAVWYYLDRETSTDVREVE<br>WVPFAGFLLFGLTFSAGLGSIGPAIAGEMFPSHLKGQASALTTITAAASSTLSIALFSALDARVGMYANF<br>LIFAAVGPVSGVFTATSGDYREAVEFQRASVLRACLNGDFSGKFEVMRRSRNNRKLPYSASGAAGSSP<br>PGKSPAEKRSRNMTEILHYLSEHR	510
BTST36	MSFRRVHGVRKLQPAISRDDAAESEHYGKMVFACGGAIMIFFFNGVVEAHSAVLLPCLQEPDSPIQIT<br>KDQETWIASLGIFAAPLSAILCGPFVDYFGRKVVIQCYFLTSALGYGIIAAATSVIHLYIGRILCSLGVGFE<br>VAGIVYIAEVCTKRQRSLCMSLSYSTFTAGILFTYVVGAALPWNLGSALYALLCLLLFLYEWFTPESPPW<br>LVKKGRSDRAVAELQRLGRTETAIAEEIKVLRLTCQEESNQRVEWHTFLQPTVWKPFLIIALFHFLQAA<br>TGMYDLLYYTVDFIDQLRTDYDSFKVSMGLAIGRFLMTSTVGSFFTTKVPRKLATAISGFSMGGTLLVA<br>AYYEYLFDGVAPGQRPYTWVPILAVFASVMVSCAGVLHLPWMMSGEVFPLNVRGAMGGAVFFVGS<br>WAMFVFLKYYIFFMETFKVTGTLLLCAAASIVTGLFGVFVLTETQNKTLQEVEDSYRRKPRKEIDVEKT<br>GL	484
BTST37	MFLTPGVRRQLAAALTCSLCCLITELMVGWAAPSLKKLREPDSPVHLTRHQEAWVVNAMYYGNIVS<br>PLPSGFLINLIGRKTTLLIVAVLPTVGWILIYFSTSATMLMVARFLYGLWTGVIMTTLPIYTGEISEPHVRG<br>VFGSFFQICNSVGSNLSFLVAPYVSIQTMAVLCGSVPIMFIVLFSQCPESPYYYVMKKRPDAAARSLFWL<br>RGGKPVSEELQVIQTSVANEEKHGSRCINLSVPATRKAFVIVVTMNILQRLSGISFMKAFSSVVMPRVGI<br>LSPDHCTIIMGVVWTVSSFICTALIDRAGRKPLLYASSLGIFVSMLWTSVWFYLNDKTDVDVSHLSWLP<br>LAGVLVYGCTFSFGLGPITKLYPGEMFPSDVKGQAAALTVMIAAFSSSVSTGLAPILNEHFGVYSNFLIF<br>SLIGLINLLFTYFCVVETKGKSLQLIQAQLRGERLDEIRDSDASEKHA	465
BTST38	MCLALGIKRQLIAAVACALATLVGGMINGWPAPTLKKLRQPGSPIHLTTEQETWLVTALHVGTLLSPF<br>PAGFMMNKLGRKASLLALGVLPVISFGLIYLSTTPEMLILARLFAGLWIGGSHTVVPIYIAEISEPEVRGI<br>LSALNQVLSFLGNILIYAVGPYVSIRSTAVFCGAVAVIFLVLFASCPESPYFHIMRGHPERAVTSLIWLRGG<br>PPTPAELGAIRGYLAARGGHGWRRITDLLTTPENRKAFVIVEVLSGLDRLSGISCMKAFSSVVLPAHLG<br>PLTSDHCTLIVSLVWMASSMVCTALIDKTGRKPLLYISSLGVAVSMFWTGMWYFLSSQTHIDVSSFSWL<br>PLAGFLVYGCAFSVGWAPITHTFQGELFPSHLKEQASAMTTIVTALTASFSTFVFGMVTKRVGVYANFL<br>WFSVVGVVNLIFVYVYVIETKGRTLEEIQAELKGKKREEPVVRASTDSIFYFN	471
BTST39	MIATLKLIKEKIFNKTLLAVIIVNSINIATGMGQGFSAILLPQLENSKEFFISQEEKSWLASLGVILNPVGA<br>MAAGVIMQFAGRKYTLIGACIPFFFGWLIIAMSTSLAMLYVGRLLSGLGMGMASAAYVYISEISTTHER<br>GLYSSFGPTGTSFGVLTVYFLGYVADWKTVAWICAATCALNALSICFMPETPSWLVSRQRLPDALRSLV<br>WLRRNELVAKKELNDIVSHAVLESQVTRKQRTILSILRKATVWKPTVILVAFFILQQGSGIYIMLFYSVTV<br>FQEIQSVLNPFVDSIIVSVVRLLTCIIGSAFIQVISRKRLVILSSFGMFFSMLSLFAYGNIASDDATARLVPW	498

	FPEMCLLINISFSMFGTLQLPWIMIGELYPLAYRGIMGGLISSVGYALIFLHVKIFPAISTVMNIYSIFLVYG<br>LFSLVAIVFGKVYLPETKDKELHEIEEIFKKKKGEVQKVDEKPNQTPIFICNPKLPFVPMQEQVVLSAPQ<br>V	
BTST40	MSQGDKMERGQESITMIPRGDVREEGKKLPQYIAAITATLGAVAIGTVLGWSSSASPFLKGEITNVTSTI<br>DPPLSVDESARVESFVAIGAIMGALPAGYFADLLGRKTLIAALTLPFLLSWIMILLAKVAWLLYVARILA<br>GIATGATCTVVPMYISEIAELSIRGTLGAYFQLMITLGIFYAYVYGYLVRFAVLNILCALIPIAGFFMFMFV<br>PESPKYLLMRQKKQSAEKSLRWLRGNKYNIKQEIETLQNEIAKSSRTKVSFKDLVATKVAFKSVNIALG<br>LMVFQQLSGVNAVIFNMNAIFMASGSTIEPAICSIIIGAIQVIVTFFSSILIDKAGRKILLLISLGVSTLSLGV<br>LGYYFHLKNSGEDVSGIGFIPLICLILFIVVFSLGLGPIPWMMSGEILAAEIKGLASSLATALNWTLTFVV<br>TRSYAPMEKTLGTDVTFWLFACICAIGFVFVVLIVPETKGKTVDQVQQLLAGKKPARKNGLV	488
BTST41	MSTPEKQPPPSYQSSEKGRFHYSKKSTYAQVLVALLQNWLLLDFGMMLAMPAIVLGSLHNHPSEELC<br>LNDAQASWFGSILFFSHPLGSLCSGFFQEQFGRKGSMMLVNIPIFIAWITLYFAESVYALYFVALTMGLG<br>IGFDEAPLHSYIGEMGEPHLRGTLCALLCAASFFGTLVMYFIGYLVPWRTAALISSAVPIITVVCMSQIPE<br>SPTWLVMQGRLKDAQKSLCWLRGWTEPEVIRAEFEQLCSYAKISPNPDQEKNPEKEKYELVPGNDNE<br>KVSPEEEEESYLKTKFKELTDKKLLLPLRMVFIVFIFCHATQLTAMRPYMVGILDEFGFPVDSKLVLIVT<br>GASVFAGTTMNMLFLRKFGKRKMTLTSQGMAALCILLLGVYCSFYDRSNRILSLAWMPVALMLLASF<br>FGGLSLALLPWQLLSEVFPLKGRGAAGGISAAWAYYVSFAMSKTYLYLEHWIKLNGVFFFYGGITLCG<br>FLYFLRNLPETEGKSLEQIESYFTKNYDRREMFSKPKRSKKVPFSA	526
BTST42	MCIPEEPSRQSREMGPFNYDRRSTYAQVLVALLQTPMIFNFGMMLSMPTIVLGALHNRKSEALWLDD<br>DQASWFGSILFATHPLGSVCSGFFQTYLGRKGSMLLVNILFFIAWITLYSAESPLVLCIAALTMGLGIGFD<br>EAPLHSYIGEMSEPHLRATLCALLVSCSWAGALLMYLIAYLAPWRTVALISSAVPIFTIICISQIPESPTWY<br>VMKGRLNDAQKSLCWLRGWTEPDVTRTEFEQLCGHIKKSSNSDQEKPQAEEKYQAVPGNDNEKES<br>VGKAESLFKTKFRELTDKKLLLPLRMVFIVFIFSRATQLTPMGPYMIGILDDFGFPADSKLILFVAGLSGF<br>AGTLMNILLVRKLGKRKISLTSQGTVAFCMLLLGVYCSFFDRSNRILSLTWIPISLLVIAGFFAGLSMALL<br>PWQLLSEVFPLKGRGVGGGVSAAYAYYVSFAMTKTYLYLEHWIQLNGVFFFYGGITLYGVWYLLRYLP<br>ETEGKSLEQIETYFTKNYDKKEMFSKPRGSAA	517
BTST43	MAIDKGKLRQYLAGFIASIGSACFGVAMGWPAPVMWALRDPRGRIRMTAEESSWMVSIMELGNLLSP<br>IPGGVLADRYGRKFVLHLAAPLFAASWIIVLLSKAKLMLYAMRVLQGLATGLVFTLTPMYLGEISKKE<br>HRGTIGSMFSVMMYVGSLYAYVFGPPFSYDVFAMICLAMPTVMFFGFMFIPETPYFYLMVGDLKAARK<br>SLAFFRSKDDPIEEELLLMQESVETDMANKSTFMDLMTEVGNRKALIILQVLSMFKIMTGICALLTYAT<br>MTFEETGTRCDANLVSISFAVVIVVSTIASAGFVELTYATMTFEETGTRCDANLISISFAVIIVVSTIASAGF<br>VDRCGRRPMLLISSAGLTLTNVLIALYFYELRLLPPLSDYAFIVFAAVGALACFHTIGYGAVHSTIQCEYF<br>PSNTRGLANGITAVTLTVFSFFTLKIFQSIDTYFGMYVNFIVFAAFCFSASVFVNEVVFETKGKSFSEIHA<br>EFRNRVSEPYVDEHHVVTV	506
BTST44	MDTPTIEAQPVSVFTKLHFQKSESSVSTSKADGMKEIEAAKPEDSPREVFTLKDEEVDESKAKTGTQYL<br>AAAIASLTGVMMGQMLSWSSPVTPLLIKEGKIDKIEESWLVSILNFGAVLGCTAAGSVNSYVGRKSVLL<br>TACLPQMASWLLLALCSDIRLLCLGRFLGGLCVGFFCVTSPLYISEIAQVSVRGALGALFQLSVTIGILTT<br>YTLGLLPTATSITLASSSTVVLFFALFFWMPETPVFLLRTSQSNRAATSLRWFRGPAYNLIPEMRLLERM<br>VQKADSAAAYSDFVTDPASRRALVVALGLFFFQQFSGINAVVFYMNTVFRTAGGDVSPTVATIVIAAL<br>QVVGTALSVFLMERAGRRFLFLASFSACTLCVFALGLFFFLKERGHPVAGPWQWVPLGSVGLFLVVYA<br>LGAGPVPWAVVGELFSKRMAALAMSLVTGVGHWASAFVVTKAFAMLEAWLGIGGTFWVFVGFCLV<br>GIVFAWALLPETKGRPLQEILDELGGKKKGVKSET	515
BTST45	MMAETNDNMDLPATNKVQANTTEDSTTNSRPPPCPQEVCDTEKGDGSRTGPQFLATGIVSLTGFLM<br>GEMVSWSSPVTPLLIKSHRITKDEESWIVSTVNFGAIIGCLLAGYVNKYVGRKTVLLSLCVPEMISWLM<br>LAFCEGAILLCLARFLSGLCLGFICVTTPLYIAEIAQPCVRGALATFFQLFIVIGILFTFILGILQDALWITL<br>GCSIVVLVFFALFLWMPESPVYLTMVSRPKAAAASLQWLRGRDYDIYAEIRVIEAVVQEGRDVEVTYG<br>DFISDAASFRGIIIAMGLFFFQQMCGINVVIFYMNTVFETAGSTISPTLATVIIGIVQVLATALSVYMMDK<br>AGRRFLFIFSQAACSLCLISLGTYFFLKSRGDDVTPIGWLPVASVAVFLVMFAFGSGPVPWAITSEIFSKNI<br>ASLALSLVTAVHWLLAFFVTKVYTSLEAFMGTGPTFWMFAAWCWVGVTFCCLLMPETKGRPQEDIV<br>DELRGCKKKQTSGNCP	501
BTST46	MAPQDTLFGSGEKKKEYDDSVDEVKSLDLETSDSEQSVNMDNMRTALPQVLATLAQSLLLLSLGMII<br>AVPTIVIGAIYKAKEGLSLDDDQSSWFCSILLIVQPIGSLLSGYVQEVVGRKISLVVVNIPQLVGWYLMY<br>AATTVDMLYWSCVTLGFSIGFMEAPTLAYVGEISQPRLRGMLSCITNSHVPLGHLVEFFIGGYVAKDW<br>RMAMAISAVFPIISILAISQVPESPVWLLTKGRKADAMKALCWLRGWTTPECVRDEFEGLVRYVEASR<br>LQNENQQKAAGKNYVQVPTAGYVNADGKGTTPAKVPAENSYKFKVTIGEKIKDLLRPAMLRPLVLV<br>VSYFFFYNCASLNAIRPYMVPVFQKLRLPKDPHFVAILSAALQVLGGLVCIATVHKLGKRCLSLISMTL<br>CAVACILIGIYAVLIERTDFDCPWFPFIVLLALYFCCNVGISPIPWMLISEVFPSRGRGAGGGVSAALFYII<br>LSIISKTYLDLESIVTFPGVFFVYGLVACAGVIFIFLCLPETEGKTLQEIEDYFTRSRKKGINNLSV	547
BTST47	MTEPDSTPPSSGCCRPFLVISSLFPLYVCVGALFGESAGMLPQLMEEDSLIPTSREEATWIASVPTIGTCIA<br>ATTSGSLSDVFGRIRMVQMAYFLLGMGYGIIGAANDFTLLVVGRFLAGVGVGCSFPANVYVSEMAPP<br>AYRGLFLVLNPLLASTGLVYMYVVGVYLPWNIAALFSCLIAFLGLLLTFFCRDSPVWLLRKNRPDAAR<br>RSLAQIEGPANVDARLKQLQEIADAQRDAETQSGHKTFSLRVLASPTVWKPYLTVLILSALQNISGFYI	465

	VISYTVNFMREFHSTFDPLQATVAIGVVRLAAICVTSALLRHVGRRTIGAVSGFGAAASLLLVYWCLVK<br>PELAPNAWTPMALFLAYIFTMTLGIFPLPWTMPYEMFPIKVRGTMCGVSFCSMYGLMFVSVKLYNTLL<br>DNLRLEGMILLFAAGSLVFGLYSATLLVETHRKTLDEIEAVFAGKRTKKVDG	
BTST48	MEDPYVPPRSDVKKISRFRQILPQIIATSATIVLYLTMGMIIGFPTILIPALTAKNSQDVLHLTMEQASWC<br>GSVGCIFQPLGSIIAGLALQPLGCKKSMMLLNIPLIACWLIVHFATSNYALYFANGLFGCVMGLTTAPG<br>LRYVIEISEPSLRGILVASTSLFISLGFSFIIFLNSLTDWRQTAAISASIPLLCIIILFQVPETPMWLVSKGRSD<br>AALKSLRWLRGWTDAETVREEYEKIVSFTKNQNQKLLKRQWSGKVAEYKNCPTVEEAELAEPASAS<br>QGLSERVRKMFKDMTRKEMLIPLTKCCIIFAINCFSGVPILRTYMVKIFDDLNLPVDPKKASVWVALM<br>GMLGNIGCMFVIKKLKKKPLFLASLAGSALCLFSMAADLMGYLEGTVIASFHRWCHLTFAMALYFF<br>WNLGIQPIAWSYLGEILPYKGRGPATSVASSFYFILTFVGIRTFPAMTEFLRLEGVLLFYAVVCVAGLFFT<br>HFLPETEGRHLSDIQAQDKEGAETEKL	513
BTST49	MSGNGKRQPVEKSFLGYPGDEVDRCKSTLSQFYATCVQCIFLISLGMQFVMPTIVLGALHNKAVINDA<br>MYLDDADASWIGSTLYICHPIGSLISGFLSERFGRKGGMMLVNIPFIGGWVLLYCATSVRGLYVATLTM<br>GLGMGFCEAPIAAYLGETSEPRLRSIFTTMTTAACNLGVLIELAIGSSLDWRTSTLVSSFVPVFSFVLFFTI<br>PESPVWLITKGRMKDAQKSLAWLRGFAKPHQVQNEFDELVRYTKMSYALGNSQGNPEEKAPLDNK<br>NNTSKHVEEDSDGWLKTRYKEITNPKLYMPLKFVMFSFFWAQCACLIPFRSYMIGILENFWFPVDRK<br>WILIMTGVVAFIGSVVPMFIIQYTGKRKLGLSCMFVATASILCLGVFASFYTHTENLVVAWLLIVDLAVV<br>HFVGFLGIINVSWMLVCEIFPVRARGIATGISTGWSCFIAFLLTKGFLWMESMVGLSGLFYMYGVLSFL<br>GCIYYYFNLPETEGKTLERIETYFTSNHDKKEKYSMPSRSASKA	524
BTST50	MIQDSTQLVSAPNSPFFTPTRIKQGWTSLLMFCAFSTIMGLAIPMGYGIGVINTPADVIRAWCNETLQA<br>NYDVVLTDKKDLDLIWSVIVSVFLLSGVVGSFIGGWLANLIGRKGAMLVSCLLSTVAGFCFLSPLIVNRI<br>ELLFAGRVIVGLSAGLGTAVVPMYLLEIAPTKLQGSIATFFSLGITIGVLLGQILGLNWLLGGETRWPYL<br>LSAYILCVLFCLLTFPCLPESPKYLFSVKNERQSALQALSRLRGLPADLLQSELDSKDTAENNFNNEEIQ<br>TWSVAQVLRTRSLLLPLALVIALQAGQQFAGINAVFFYSSDIFKSAGLDETSREYAVIGTGCVNLGVNVI<br>AVFTLKYFTRRFLVLLSCYGTVLSLLLLTLCSHYMQTVSWLPNASIAVVMLYVFMYGVGLGPIPYFIGSE<br>LFAVGPRPIAMAFGSFANWGGNFLVSLTFTTFFNYLAGYSFLIFAGSTMLLSIFIHAYLPETKNSVASL	488
BTST51	MVVATGDFVSTSRDSFIRPLLASVPVFWLQLLTGSIEGHSAVLLPQLEESEKFYISLEEESWIASLGIMATP<br>LVAVLSGPMVERYGRKFIFYIFYILCTLGFSFIGLAQRVEHIYIGRILGAGAHGLTLCSILYIYEICVAHQR<br>NKLLPLLCPMCSAGILYAYVIGGYLPWNVASLVLAGSGVVGLICIFFIPESPAWLVMQGDINAAIQSLE<br>WLKRDKETIAQEVDELRKSSTSKDLPQSISLQHFLHPTVWKPFLILLIFSALQNGSGFYMLLYYTVNFFQ<br>NLGTGGEIDPLTITVGLALMRLVSGSFGALFIARFSRKKLTATTAFGMFIVASAAVAYLVTFGEDPTNRP<br>HQWFLVLCSLSYVLLCTLAIQPLPWLMTNELYPLQLRGLMSGITFFCLFTMVFVGIKAYPFFMFYIHITG<br>ILCIFAGACLLAVIFAVFFLPETYNKTPYEIEEYFMRRKKKSVHCVNFYEKTCVQIPLKFKEFASYYGEHS<br>LFARKSA	501
BTST52	MMAIKKELNLDNPRESYVRPIAAGAILGMQLINGIMEAQSAVMLPQLAAESSAISITQSQSSWIASLGI<br>VASPISSVLCGPLMDFFGRKMILEGYYVIAMLGFLIIACAKKVVHLYIGRFALSIANGFGVGTIVYLPEM<br>CSRDQRSRLAALLMPLFSSGILTAFLVGGYLPWNVASASYTGVCTIGLICSVVWTPESPCWLVNEGRYE<br>EAKRSLRYLRGSDSTNIEAHVEVLKMSRKSVLVERNAVFSDLLEPTVWKPFVILVLFHFLQTGTGFYGL<br>MYYTVDFFDDLRTSFDPLTVTIFLSVARLVMSCVFGTYCATRLNRKVVTALSSGLSGVSLLGAAAYEHV<br>FTSIDADERLHTWIPIGCILTNVLVCTITVQPLPWLMTRELFPLPVRGIMCGLTYFIGTVLVFLSVKYFMS<br>VMGLFGIPGALSFFSASSFLVCLFGIFVLPDTNNKNRSEIERNFTKAKPRVEEQPLIGKGVGNVMPTLLV	487
BTST53	MVFACGGAIMIFFFNGVVEAHSAVLLPCLQEPDSPIQITKDQETWIASLGIFAAPLSAILCGPFVDYFGR<br>KVVIQCYFLTSALGYGIIAAATSVIHLYIGRILCSLGVGFEVAGIVYIAEVCTKRQRSLCMSLSYSTFTAGI<br>LFTYVVGAALPWNLGSALYALLCLLLFLYEWFTPESPPWLVKKGRSDRAVAELQRLGRTETAIAEEIKV<br>LRLTCQEESNQRVEWHTFLQPTVWKPFLIIALFHFLQAATGMYDLLYYTVDFIDQLRTDYDSFKVSMG<br>LAIGRFLMTSTVGSFFTTKVPRKLATAISGFSMGGTLLVAAYYEYLFDGVAPGQRPYTWVPILAGRAPP<br>PLDDERRGLPAQRPRGHGRRRLLRRLLGHVRLPQVLHLLHGDVQGHGNAPLVRRRVHRHRSLRGLR<br>PHGDAEQDAPGSGG	428
BTST54	METFKEQKSPPPENWIKTFWACGGAITILVFNGVVEAHSAVLLPQLQEAYSPIHVDKDEETWIASLGIS<br>ASPLSAVLCGPCIDRYGRKIVIQGYFLISAIGYGIIAAASSVVHLYIGRIICSLGVGFEVAGVIYIAEVCTKY<br>QRSLFLSLTLPMFTGGILFTYVVGATLPWTMGSSLYALLCLLLFVYESFTPESPPWLVKQGKISRAKAEF<br>KRLGRSDEWIEEELKLLQASCDTLERNHHLDCKTWLEPTVWKPFLIIALFHFLQAATGVYDLLYYTVD<br>FVGELGTQYDPFQVSLYLAIARFLMTSTVGLYFTSKVRRKTATALSGFAMGASLLVAAIYEQRFDGVAP<br>AERAHTWIPILAVSVSVLVSCAGVLHLPWLMSGEVFPLRVRGLMSGYVFFVGSCSMFVFLKSYVFFVE<br>VFKVTGVLLLCAAASVLIALFGLFVLTETQDKSLYEIERGYEKKSRGDADKTNLREVE	475
BTST55	MKLMIGDEEVCVKIGNDGKEELSWRCWLRTMFAASGAMAVFVFTGVTEAQSAVMLPQLKQKDSPIQ<br>ISADEETWIASLGILLTPVSAILAGPLVDAFGRKKGLQGFYIIIGLGFGVIASAKEVYQIYIGRCICAFAVG<br>MEPIAVIYLAEISTKRQRSLFFSLMAAMYSGGVTITYVIGGFLPWNVASAIFSLGCFAIFVVQCVTPETPA<br>WLYKTNQVEASTESYLRLGRSHTNILQELESLGLSSQQRTEKFHIRAFLEPTVWKPFLILSLFHIIHCGA<br>GIYDVLFYTVEFVETLGTSYDPLAVSIFTSVARFITNMTVGLYFTASLSRRFATIFSSFFMCLSLFVMGVYE<br>YLYRDVSVKPFDWVPVLFTVVSVVSCSTGLLSLPWLMPGEMFPLHVRGVMNGAAFLVGSACMFVTL<br>KLYAFCMETLQIWGMLLMFAGFAFTGIFFGMFVLPETQGKTLYEIEQGFLPTQKKRENEISPSGKVETIT	487

BTST56	MAFVVDTEVACAKIQSSDEDESEKLSWRCWFRTLFAASGPLMVFLYTGVAEAHSAVLLHQLKKEDSQ<br>IPVTTDEATWIASLGILLAPISALLAGPVLDAFGRKKGLLSFFLSMGLGFCVVAFAEEVYHIYIGRCICAI<br>AIGLEVTSVVYLAEICTKRQRSCFLSLTAPFFSLGVALVYLVGGYLPWQMAATIFSLSSFGFFVIQCFAPES<br>PAWLFKTGQIEASTKSLRRLGRSHENILHELDLLTLSTRTRSGKFHLRAFLEPTVWKPFLILSIFHFITNA<br>AGVFDVLYYTVDFVKAFGLTVDPLIFTVLLAVARFTTNCTLGAYFLVSVPRKFTTAFSGFIMAASLLGST<br>VYEYAYRGLAEKPLQWIPVTLTVIAIVASAMGMNFLPWIMPGEMFPLQVRGAMTGASFLVGTFCTFVS<br>LKIYGFYVETFCIWGLLLRFALFAFAGALFGILVLPETQNKTLYEIEQGFVAGNKKIPEAPEQ	482
BTST57	MTSSVSDEETRGKISSNEETKDKTVSDTEIGAKPVNDEECGEEVSWRCWIRTLFAASGAMMVFVFTGV<br>TEAQSAVMLPQLKKPDSYIRVGPDEETWIASLGILLAPPSGILVGPVIDAFGRKKGLLFFFLCMGLGFAV<br>IACATEVYHIYIGRCICAFAVGLEVVAVVYLAEISTKRQRSGFFSMMSVVFSGGVTLTYLIGGYLPWYIAS<br>AIFSAGCFAYFAVVCFAPESPAWLFKTGQIDASTKSFLRLGRSHVGIVAELENLKLSSKEDDEKLEFKAF<br>LEPTVWKPFVILSMYHIFQCGTGVYDILYYTVDFVESLGTSYDPLPVSILLSVARFVTTATLGIYFTASVSR<br>RFATAFSAFWMAVTLAGTGVYTYVYRDTTQKPYDWFPIVCMLINIVASALGVTSLPLLMSGEVFPLRV<br>RGAMTGASFLIGLGALFVVVKIYAFCLQILQIWGLLFVYAVFSVLCVLLGVFLLPETQGKTLWEIEQGFL<br>PKKERRRNGERRTEDTLGSGVIRK	513
BTST58	MGKVEMHSSEKVYRPCEMTTSNVNNVKSTVAQFYATFVECCFLILVGMIYMMPTIVVGALHKTESSN<br>STLTSPEDEAMRMDDHTASWIGSIVLMSHPVGALTSGFVSERFGRRGAMMLGNVPFLGCWVLYYLAT<br>SVKGLFIASMLMGFTIGLCEAPMGAYLSESCEPRFRGISNSMVVAFCTMGNSLELFLGSAFHWRTSALV<br>GVSVPVICFLGFLTVPESPVWLITKGRLEEAHKALAWFRGFAEPQHVREEFDDMVRYSMASSRLSRHD<br>LSAISEEKAPLDGKHNIQDPEKSQSIKRGNWLVERWRELSNPRLYLPLRMVLITFFFTQSAGLVPFKAFII<br>EILNEFWFPFDNKWAVVATGVASFLGSVGATILVKLAGKRLMCIICMIISTISIFVLGFSASFFRHQEDLLL<br>SWLILSVFAVVHFVGNVGVINIPWMLSYEVYPVRARGMANGISAASGCFMAFLQTKTYLDTERAIGL<br>DGVFYAYGVVALAGCIYVILYIPETEGKSMEQIETYFTPHHDRKEKYRMPSKKNRSKA	539
BTST59	MSEKEKLHSTEKVYKPCEYSTENTGNFKSNLAQFYVTCVECIFLISLGMQYVMPTIVVGALHNKVGDS<br>MALDDTTASWIGSILYFCQPLGSVTSGFLSERFGRKGAMMLVNVPFVAGWILLYYATSVQGLCIATLTM<br>GLGIGFCEAPIAAYIGEVSQPHLRGIFTAMTTAACQLGNLIELFVGSVFDWRTSALISTVIPLISLISFTTIP<br>ESPVWLITKGKMEEAQKALGWLRGFLEPHHVQKEFDEMVRYARMSNTLSSEPGENFSEKIPLDSEKIP<br>IKEVSDGFFKQRYRELTNPKLFLPLRMIFITFFFTQTASLAPFRAYFVRILDQFWFPIQSRWVLVMTGATA<br>FIGSVTAIFILQKTGKRRVMLFSMAVNLLATFILAIYATFFTHTVDMTVSWILIVTFGISYFVGSLGINNIP<br>WMMLCEVFPVRARGIASGLSAAWSYFVQFVMTKTFLQTESLIGLSGMFYMYALISVGACIYTYLCVPE<br>TEGKSLELIETYFTKNCDRKQKFRMLKRGRNPSKA	524
BTST60	MISSGKTQSPNERPYQYEYTALGTQDVEKSGEHGTGVSGNRRKVNRFRSAAPQILAVTAKNLVLLDL<br>GMTMAFSTIVVPVLLDPNNKDPNGLSFTEDQATWFASIPMVFQPLGSALSGLISAPLGRKRSLMLVNIP<br>QIIGWLMLYSSSSVNIMYLAAAIQGLGAGFMDAPIFTYVGEICEPSLRGVLISYSLQFCSVGFFLQCLLGS<br>LTTWRHVAFISMLFPTLAFLAISQIPETPMWLLSKNRMKEAEKALCWLRGWVSKEEVAEEFAQLVQYS<br>KNSKYKSDDDKKKLQMDLISTAKQPCGGCTRPPIVPCDSNTGDDDYAKLKLHEKVKDLLRPEILKPM<br>SIIIIVNFLYFTSGFPGFKTYMVLLFQRVHSPIDPNWASVFVSTSIILIHIAQMVAVKTIGKRWMTLISSFGA<br>AVAGLAIGVHMSFQGFFDETFGDLSNWLLFTYFEILTLATVIGLGPVPWMLMSEIFPFRGRSFASGFCA<br>AIYYAASFFAAKTYLSTLNLFGVAGTYYIFGTISALGLVYVYLYLPETEGLTLEEVEDIYRPKKRSEVKNL	555
BTST61	MSDTTPSREAPAPTMTTESPANSLDVYYIPNINNEEKNYKSNCKSTLSQVIATLVESLLMVVLGTQSVM<br>PTIVLGALRNNPHETLSLNDYDAAWLGSILFLCQPFGSVASGFLSEKFGRRGSMTLINVPFIVGWILLYY<br>ASSVTGLFAAVLVMGIGIGFCEAPIAAYLGEIGEPHLRGSLLCIMCSAVSLGYLSTFFLGSIMPWRTFALV<br>NVIYPVTTMILFTQIPESPIWLIHKGRLKEAQKALGWLRGFVEPRRVQQEFDRMVKHIEASKSSDRPKG<br>NVESQDDGSCESKFTVIKRICELRNKKLYLPLRLVFITFIFTQCMCLQAFKPYLVNILDTFKFPVDSKWV<br>LVMIGLMNFVGSAMPLFIFRFTGKRQLILCNQFICVVGVFALGLYCSFLNDTLDTNSDWRWLPIVLFAI<br>VFFSASTGIMNIPWMLMGEVFPIQYRSFANGLCGAWAYCVTFVTARLYLPMEHVLSLSGMFYLYGIVGI<br>LGFFYFLFFLPETEGKTLEKIESYFTPHHDKKEKFTRPKR	527
BTST62	MAKNEDEPPTGGFLRPFLIMTALAPIQLVVGSVLGQSAGMIPQLMQEDSVIKIDIDVATWIASMSTVGT<br>FVAASSSGFFADKFGRIRMVQVAYFFLAIGYGIIGAANTFFLLIFGRRLIGFGVGCSFPASVYISEIAPPAYR<br>GLLLTTNPAIASLGLVYMYVLGGYYPWNIASLATGLMSILGLIIAFFFYDSPVWLLRKNRIEDARKSLSRI<br>EGPTNVDVKLKQLQEIVDSHPVSKFSPHVFIEPTVWKPYVITIILSILQNTAGFYIVVSYTVNFMREFHST<br>YDPLQIMVAIGLVRLVAICLSSAILRHVGRKTIGAFSGFAAAACLLPIYGCLVAPHAAVLRTYPWIPIALF<br>LGYIFTMTLGIFALPWTMPYEMFPIKVRGFMCGVSFCSMYALIFVAVKLYNFLLENLQLPGMILMFAVG<br>SLLFGVFSATVLVETHKKTLDEIEEVFLGRRSKKIQKTTSDS	466
BTST63	MFSVSILPFGFGQFATWPSLAIEQLLEGDAGFSVDQSEISIIASTWSLGLCLLPILFGFMLVRQGRRRNLLI<br>TAVVYIVAWALILFARSPLWLMAGNFIGGLGSSIQLIIGPIFIAEIADKYIRGALISFYIAAIPLGQAFMCSV<br>GIYVTYFQLNLIALVISTVAFFCILATAVESPSWKLMKQKESEAESCFNYYWNTRNADRTESTVALAELR<br>ETVELEMRSKCSYSELVRTPSNVRGTIIVAAISLFQSASGILVILDYGSTTLPKYEGFWAPHPTMAAVSILY<br>FFLSLVSAGLVDRLGRKPLTILSNAGDALGTAIVAVFFALERRTEWDTTNLQWLPYVGMLLFIASYGSA<br>MSAMPHVLVGELFPANVRYHASVLSVIAIAGSLAFFNYTYLGGCRLLGMDVMFFIYTLCSIAATIFSWL<br>FMFETKNLSLAEIQAIMTGRKMASNDPPQELNDLR	460
BTST64	MVQLAWLSRNRGRVRQISVCCSASILPFGHGLLVAWPSLAIERLRRGDAGFEVSSGEISIIVSMMSLGM	477

	CLMPIPFGYVLVRLGRKTNLLINAVVYAVAWGLIAFAPSPLWIMVGNFFAGLGSSIQLIIGPLYIAEVAD<br>KDIRGALISIYIMAIAIGQVFMTSIGIFVSYFELNLMSLIIAIVAFFCILTTAVESTSWYLMVDNEYEAERSF<br>HYYWNTGAGTDRTQSLATLKETVALEMKSACSYVELFRTPSNIRASIIVIAQSVFQSAGGIVAILTYGST<br>TLPAYDGFWKPNPTMALVSVLNLVFNIVSAGFVDRIGRKPLTIISNAGNALGTAMVAAYFAVERRTNW<br>NVTDLEWLPYVGFLTYVAFYGSGMFTVPHILVGELFPVNVRYHAAVLSTISIAGSCAFFNYIYLGVSQV<br>AGVDVMFLIFTLCSISATVFSWIFMFETKNLSLADIQAKMTRNSTRASDPTPELENRR	
BTST65	MVKDEIEIELSKEYDDSVRCPVNRLGKFRQCLATFIANIITICLGTVNGWAAPVQPQLQSETPPVGRRL<br>SDDEISWLGAITFMGGVAGVLVWARAADLLGRKGAGYLIAAPFLLSWTLLLFCDHYYLLLAARFIAG<br>FGGTGVLVNTPLYVGEIACAQLRGPLGSSLILFINFGYLLAYFFGSVLTYARFNLFCLLLPVVYLALFAYL<br>PETPNYLYMNRREDEAKRSLLYFCGDNARAMNHEFNLIASVTNGGPRVELSDFLRKKSTRRALVIGM<br>VLITGQQVVGINILLTYTVAIFSAAGSAISPNLCSVIVGVAMLIASIPSCYLINRLGRKYLLIFTSTGMSASL<br>LLLAVCFLFDKSNAFVQSTYLPLVSFSTAIVCYALGVGPVPFVLSSEIFPSSVRNMATSLIIAWGIFGSFAT<br>VKLYPSMLSLLGYFGTFSLFSVSALCLSLFIHFCVPETKNLSLNAVIELLENHSTLKVGRFS	481
BTST66	MASLGIVTAPIGAILIGPFVDAFGRKVGILIFYLTIGSGFGVIALSMDVTQIYIGRIICAFCEGFKACAVVYI<br>AEICTPTQRSLFLSAISTMFSGGVLICTVMSAFVSWNSACLAYSLAAFAFAGVQWFVPESPGWLYRHG<br>KEDEALRSLERLGRSKADILREMDDLKERKSNQEKLELKSFFEPIVWKPFVILSTFHVLQFSTGIYDIIYY<br>QVDFIQSLGTTYDPMTVSVAMSTIRFLSNATIGVYAKSVSRKGSTALCGLGMALTLLATGAYELAYRDT<br>EIPARPYQWLPISLILSCIVASNLSVTCLPWAMSGEMYPLRVRGIMSGATLVVAYFAFFFYIKMYYVFLEA<br>LKIYGVLFVFAACSVVVLLFGIFVLPETQGKSLLEVELGFEKKAKRSENVENRNGRVEKGEKSDFVTRF	421
BTST67	MLIEDLHVECIIAADVTFSRVKLIVRLSSNYNIECLVEKEYLAGATRQFLILSSISPQNRKLRVQAAPLKTI<br>DDMAGEPEIITWSCWLRTVVAGITALFLLAFAGMNNGASNLLLSQLTKKDSLIPISQDQESWVASLGL<br>LAAPIAPILIGPFIDFFGRKKGVLVFYLIMGIGWAVIGSAKNVTQLYIGRMICSFGEGFEACAVVYLAEIC<br>ATEQRSIVLAWLRALFSAGVLFVDVINTCVSWPVACLGFSLAAFAFAIAELFVPESPAWLFRQGEEEAA<br>VKNLQRLGRSQAGVHLEIETLRQRESSTESLSWRTFLKPTVWKPFVILAVFHVLQLSTGCDVIIFYQVDF<br>LASLGTTYDPVSVSVALSTVRFLSNITVGVYTNSISRKISTAISGFCMAVPLAGAVIYEYHYRSVPVLDRP<br>VQWLLLTFIFAYLVAAELAVNCLPGTMVGELFPLSVRGTMSGATHFAAHCSYFAYVKFYFACLRVLKIH<br>GILFVFAASSFLAGLFGIYILPETHGKSLVEVEQGFEGKTQEIDQNIVVPLSSINS	546
BTST68	MITVASAVMENDTNSCRKASVENGLKTDISNNRRGKRRFRSASAQILACVIQAWLLVDLGMEMATPT<br>LIIGALHKISAEAEPLHMNDEEASWFGSISNMVFLFASLSSGFLQELIGRKGSMIVVNVPRFAGWMTLY<br>FASSLSTMYLAAVVMGICEGLCEASVHSYIGEIGDPRLRGTLASISSHGYFFGTLTTLILGCYFEWRTVVL<br>ISSAVPVLAFICLTQIPESPTWLIVRNRLDEAKKSLCWVRGWVSPDEVEEEFQEMVNYVKNSSEESLKA<br>FNSNECAESNAKDLTVFKGVLSTMKAVASKKVLRPLCMVCTAFLTSLAGNVIGITPYMIRELRELGAIV<br>EPKLILVMFQIIFVVGSLTNVAFVRRFGKRRLALLSQGLAVLCILGIGTFCSLAFSSADRSPQLSWIPVALF<br>FFLNFINGVGVRLLPWQLLSEVFPPVGRGFASAISVAFAKLILFTLIKTFLMTEDWLHLSGVMYLYAGVS<br>FFGLCYYYLYLPETEGKTLEQIESYFTKNHDRTEKFRIGNQDRNTLY	534
BTST69	MGASMNACTRTPTEDASKTTAKRHPCRSTCAQLLATLIQGWLFLDLGLEAAVPTLIIGALHRNPSATE<br>SLRMNNDQASWYGSLQSFCFPIASLSSAFLQELIGRRGCMMAVNVPSFAAWMTLYFAESVPALYVASA<br>IMGLSSGISEASLHSYIGEIGEPRLRGTLSSLSSSGFCAGSLGGFILGYYFDWSDWRTVVLISSACPVIAFIC<br>MTQIPESPTWLIVRNRMDEAKKSLCWLRGWVSPNEIEEEFQTLVQYVKNSSAETRRGLRQETSIASTEE<br>NSSILKEFILIIKVLASKKVFRPLRMVFITFVISSVACVGGIRPFLIGELKDLGTTIDPKLVLIMFEVIFFVGS<br>MFNVTFVHRFGKRRLAIYSHSFAAIMITGMGVYCSYSSFYEDNSNSQLPWIPVALFAILNLVEGVGISLL<br>PWQLTCEVFPPVGRGLAAGMSAAWSKLVFSALIKSFLYLEVWLNLSGVMYLYAGLTVFVSLLLSTRNG<br>GEKSGADRIILHESSHPEGEN	511
BTST70	MTETEKNETSGQEEAPSEPVISNTTYRQIVLALILAIPSIAPGMTFGYSAVSLDSIPANLSQESWFASLAW<br>IATPVGCLASGPIMDNWGRRPALLLINIVGFCGWILLAYASTTLSLYTGRILTGASIGFASAPSSVYVAEC<br>IASNSLQLRGILLTWPTVALSTGILLVYIMGSLLRFTVVAGLGAIISVASFFCILFFIPESPAWLLLKGRRED<br>AEVAQRRLGLGKPLSESRVESGEASTSTKLLPSQSELTWSTAWEELKKPEAYKPLTIVIFFFLFQQFSGVL<br>VVINYLVEIVRISGFVLLNPYFVTVVAGFIILICACSVSFLLPKFGVKGLSTISGVGIAISWLIIGLYIFIRRTW<br>LVELQYSLFNLIPLCGIILNVVSSSIGFYPLPFAILGEIFPPKIKGVASGIATCVAYLFSFIAVKTFIYLQLHFY<br>SAVIFFYAVMAAFGVIHVNLFLPETTGKSLQEIVKHFSATKSGYEKI	483
BTST71	MGAEDAASAESFLKPFLAALASFMCQFQLGAILGQSSTMLPQLQAEDSPIRITKEYASWIASAGVIGTP<br>IASVLAGPLTDKMGRKSVIRMHFLLSAIGHTIVGVSSDGTEILIGRVILSCATGFGVPSLVYIPEICNPRHR<br>SPLLFTATVSSSLGLVYVYTLGGILSWDITAMLTSSLAIIGLVYTFIVPESPAWLFRSHRLNEAIDSIKWLK<br>GQNVNMELELRSLKDACHEQPKERVSLLKQFASPTVIKPFLVLTIISFLQNASGFYILLYYSIDFFLEFKSS<br>YDPRFVSVGLAVTRLVSCTVASVIINRFCRKTMGTFSGLSMGVILLGILGYLHAFGDDVEVLSRYSWVP<br>AAGLTLYVFACSLGVHPLPWLMIFELYPLEVRGRMCGISNGMCYVFTFVFTKLYYTFIANFKIQGTILLF<br>MVASVLFGLFSAFVLPETQGKTLVEIEDRFRPKKKPDKESTLP	467
BTST72	MSLEAEKLEGLNTQNEAVTIIKSRYNYSRRSAFAQVLATLIQNWLLIEIGLDTAMTTMVIGALHLNSAE<br>ALSMNDEQASWFGSLPFICHPLASLLLSGYFQDRFGRRTTMILVTIPTFIAWVSLYFAQSMYVLYMVSA<br>VTGMCTGLTEAXLHSYIGXIGEPHLXGTLSSISTSAVXVGIFMMYVFCYXFTWRTVALICSACPVITFTC<br>MTQIXESPTWLIVKNRYEDARKSLXWLRGWVDPSEVEEEFQALVXHARNSXQKNKXAQSXGXGLIKK<br>DSYLKTXFKEMTSKRVLLPXRLILIVFVFREITTFSAIRPYLIGELNKLHTPINAKLILILSEVLVFVGAMM	511

	NVVFLRRLGKRKIAIFANGIXXICILGTGIYCSFLQDSTRXPQAAWLPXXXXLMLSLFCGFSATLLPWQLVCEIFPIVGRGLATGITAGTKYLIQSAMVKSYLFIETYIGLSGMMYLYGTGAVLGVIHLYFCLPETEGKTLQQIESYFTKNHDRKEKYSIGKAA	
BTST73	MTQASDETEKLVESPSTTAVYRQSLAALTCCLSCFTIGLSIGWSSPAFHKIQASETSFTLDGFQQSLVVSALNIGIMFGAIPTSFLMDQLGRKKTLLYTATLSLLHWVLIAGAMNAKFLYFGRFLGGIYSGIATAIAPVYLAENLEPQIRGSIGTLFSILLYGGILCTYIIGPIASYMNLSLFCGAFTVLFMVTFAPMPETPYFCIIKNRREDARKSLEWLRGHSNVDAELKQIEAYVTSEQEHVTGWSDIFTDPNLRRPFLVCVALCFIQKSTGFFTIISYQSVILPGMVGPLTSEGATLVIGVVLLMAGTASAFLIDKVGRIILLNLSYVGVVLSMIPTALWFYFNKTDEPADVEYVNHYNWVPFFGFIAFIVCHAMGLGPVGNIYPGEVLPLSIKADAMALVVSLAALFTAINTEIFAFFNAYIGMYANYFSYAVVAVVGALFTRLYIVETKGKSLQAIQEEFIEQAKMPRMKGDYFVL	482
BTST74	MTVGAIAGWSASAFPKIRNDELKFRLTLFQEAWVINTYYVGIMMGPLLAGIAMDAIGRKTTLLLFSIFSVANWTLVILASNEYMLYIARVFSGLWAGSVFTVCPAFLAEVLQPHVRGSLGSFLMSMYFLGNLYEYIIGPYVSYSTFGIASCIPCLIFAVAFLFIPESPYYYIMKNQRGKAEASLSWLRGDVDVNQELDAIETYAVAFMRNRGSFKDVFLNENYRAALINVQAIYFLQKLCGMFTVLAYLTVIIPPYVGPFTSENCTLIVGVVLWISTTLAASLMDRIGRKRLLVISNAGIIVTMTITGAWYYLDSTDLDLSETTYVPFLGLVIYGIFFCLGLGPIPTLYQGEILPSNIKARACTVTTMCSAWASILNTTLFAICIRYIGLYINFFLFAATSVFGLYFAKYHFIETSGKTLQEIQEELMKRRHRGKFTDDKKSPVNAKPTIYTVPMPNATEKVETKKHFEKDAKWTDE	477
BTST75	MFKISRSIFRQTLAAFCCSIGPMTVGAIAGWSASAFPKIRNDELNFRLTLFQEAWVINTYYVGIMMGPLLAGIAMDAIGRKTTLLLFSIFSVANWTLVILASNEYMLYIARVFSGLWAGSVFTVCPAFLAEVLQPHVRGSLGSFLMSMYFLGNLYEYIIGPYVSYSTFGIASCIPCLIFAVAFLFIPESPYYYIMKNQRGKAEASLSWLRGDVDVNQELDAIETYAVAFMRNRGSFKDVFLNENYRAALINVQAIYFLQKLCGMFTVLAYLTVIIPPYVGPFTSENCTLIVGVVLWISTTLAASLMDRIGRKRLLVISNVGIIVTMTITGAWYYLDSTDLDLSETTYVPFLGLVIYGIFFCLGLGPIPTLYQGEILPSNIKARACTVTTMCSAWASILNTTLFAICIRYIGLYINFFLFAATSVFGLYFAKYHFTETSGKTLQEIQEELMKRRHRGKFTDDKKSPVNAKPTIYTVPMPNATEKVETKKHFEKDAKWTDE	499
BTST76	MFIIPKRVRRQIFAALSCCIGPLMVGSIAEWSASAFPKIRSNELGFRLSVFQEAWVINLIYAGIMVGPLLAGIAMDAIGRKSTLLLFTVFAIINWTLVTFAPTKHITLLLFTVFAVTNWTLVTFAPTKHILYLGRFCGGIWNGCVITIVPAFLAEILEPDVRGSLGSLFVMMYFAGNLYENLIGPYVTYRSFCLISSAPVFVFAATFVFIPETPYYYMMKGQRKKAEASLMWLRGDGDVTVELDKIEKYAETFMKQRGSFKDLIFNEKYRKAFLNVQGVYFIQKLCGTFTVLAYLTVIIPKRVGPLAPSNCTQITGIVLLLSTFSSTFLLDAVGRKPLFIISNIGIIVTTSITGAWYFLDGHTDFNMAGTTYVPFLGILLYGGFFCVGVGPIASIYQGEVLPSNIKARASTVTTMISAFASIVNTTLFAVCNRYIGIYVNFFLFALTSVFGLYFAKYHFIETKGKTLQEIQEELMMSYQKRKASALSGKLGVCQVPIPHSVKTATKR	507
BTST77	MTEEETKEKLSWSCWLRTMFACSGAMMLFVFTGVVQAQSAVLLPQLKGNDSIIHVTPEEETWIASLGIFMSPVSALFVGPFIDVLGRKKGLLFFYINMGLGFSIIACASKVWHIYLGRCICSFAVGLEVAAVVYMSETCPKELRSIILSISSATLTIGISITYVIGGYLHWALASAIFAVGCFVYVIIQALAPETPPWLFKQGFKDDATRSLQQLGRSPSGILREIKLLEISAPEHTERLSIGTFLDPTIYKPFLIIFAFMFLQVLTGVYHIMYYTLNFVERLGTTYDSLQVSIIIALARMLANLTLGGYSTAFVSRKWATALSAGLGAIVLALAGAYEFLYRSVPVGQKPYEWVPIALVVVNIAASMIAVTPLPWLMGGEVFPLRVRGSMSGAVFVVGSAMMFVFIKIYEELMELLQIWGMLFFYAVASVVMVLFAVYLLPETQGKSLFEIEQGFLPKNKRLSREPEPAGGATS	475
BTST78	MTTDMNFGAKPESRKAILIQIISSVIASSTLLSSGMSLGFSGVALPHMEAPDSLVKVGPQEASWIASLANLATPVGCLLVGPLLDRLGRKNTMIFVGVPAVCGWLLIAVEPSLPRVYLGRLLTGLATGLSSIPSTVYTSEITSNAMRGILVTCSSISIAVGILTEYCLGWWFQRHWHCVALVSGVISILVSGLVLIGIPESPVWLVSRGQNQEASKALCTLRGTKSKNKIEKELNQIIENCRAYRGRSTSIARSISGLALPQAYKPLIIMNTYFLFQQVSGLFVIVFYAVDVIKIAGVTADAYLIAVLIAFLRLVTIIVSVWVNKAFGRRFASIISGVGITLSMFALVGYCYFVPGAAAPTPVLVNSTTTTTAIPQALVGSTDAPIPMANFSLVENVTVVMSESFQGVHGLSWIPIAALFVHIVFGTIGFLTVPWCMIGEVFPAQVRGVACSITSCFAYLSSFVVIKLYKSMLMSMGTVGIFTFYGIMSLLGTLFVMIYLPETKGKSFEAIEKHFANGSGVPASPEEVSLQTKNSKQPIIRPSRPN	546
BTST79	MAEEWTXTAPPKASFLRSFLVAASMFPLYICLGALIGQSAGMLPQLLEEDSTIHINKNQATWIASLPTIGTCMSSAASGYLSDLFGRIRVVQAAYSFFAIGFATMMAADSFMLLALGRFLAGIGMGCYFSGNVYLSEVTPPKYRGALLTLNSVLCSCGLVYVYIVGGYYPWYIAAAATCLISIIGLTLTFSLYDSPVWLVRQNRLKTAAKSLRLVEISSNVETKLRKLQETAENHPKTDFTLKILTEPSVWKPFVMILVLSILQNTSGFCIIIAYTVQFMWEFHSAYDPLHVTVAIGVMRLLAILVSFVLFQHFGRKTIGAVSGFGAAIFLLGVYGYLIFAPRVQLLSENQWIPIVLFLAFIFTSSLGIYPLPWILPFELFPIKVRGMMCGACLCALYLNTFVAVMLYYVLIDNLRLGGTILLFAAGSALFGIFSMTLLVETHRRTLDDIECTFASGRVT	460
BTST80	METQLPPTGCCKPFVAVACLFPLQILVGAIFGQSAGMLPQLLEEDSWIRIDREEATWIASLPTIGTCVAATVSGSLSDTHGRIRVTQVAYFFIGMGFAVMATANNFTMLALGRFMGGLGIGCYFPALLYVSEIAPVAHRSILLALNGLMASAGLVYIYILGGYYPWPIAATASCLLAILGLLLTLFLYDSPVWLVRHDRLETARKSLHRIENPANVEATLKHLQETASNQPKCDFTLKVFVEPIVWKPFLIILALSVLQNLAGFYIIIAYTVQFMREFHSAFDPLQVTVAIGVFRLMAIALSAVLFRYFGRKTIGAVSGFGAAACLLATYAHWKFSSMVALLAENQWIPVMLFLAYVFFMSMGIFPLPWTIPYEVYPIKVRGMMCGVSFCSMYVIMFVAVKMYNILMDNLRLEGTILLFAAGSLLFGVFSVTILIETHRKT	441

BTST81	MDKKTAFSIEVLEPVVRKESKKTTQYVAALTATIGGFIAGNILAWSSPAGPKLMDGEYGFPVTEDDMS<br>WVGGIMAIGAIIGCIITGLTVDVFGRKNLMLFLVAPTTIGWCAIIWAESVFILCCGRFLLGAACGSFSIVC<br>PMYTGEIGENSIRGTLGTYFQLQIVIGILFVYLIGSILNTFWMSITCAVIPLVYAGLMGLMPESPTFHFKK<br>GEVENAKMSLQWFRGPEYDINGEIKEMLDIIDRDEREKVPLAIAIRSKAAKKGFVIGLGIMFFQQFSGI<br>NAVIFYTTQIFQSAGSTIPPDLCTIMTGVVSVISCYIATVIVDKLGRRLLLLTSGTVMALCCGVLGGYFY<br>MLKHNMDVSNIGWLPIACVCGFNIAFSLGFGPIPWMLVGEIFSSQIKGTASSIACLFNWACVFMVTKF<br>FSVIAEMFGSYSTFWFFTAMLVTAIAFTFFVVPETKGKSFEQIQSELSGENESQSEASTVSAYPSKDLKY	487
BTST82	MSKNDRCCGVSLSVYRQFLSAIFCCIGSLSLGLSLGWAAPAFVKIKNGEAPFELTIYEQSIVVGALNIGL<br>MIGTYPAGYLMDRIGRKTTLLYASSFSLINWILIAFASSELYLYVARLFAGLWAGAISTLVPIYVTECSETKI<br>RGSTTTQHLVFMSAGILLGYIIGPQVGYMDFALICGAFTVFFAIVFSFPPESPYFLTMKGRTEEARAALV<br>WLRATDDVDNELKSIEAFIADGTKGRCSYKDLVKNPLYRRPFFICLVLWFCQKFTGFYTLIAYQTVILPK<br>KLGVLTSDNCTQIVGVIILTSVFIASRLMDATGRKVLLTVSHVGIAVFMGIVGALYALNDTGYIQIEDYS<br>YLLVFSFVAYVFSFSIGIGPIASLYTGEVLPQAAKGTAGSVILSLSSIASAGNTFAFAATANWIGMHWNFF<br>FYSALSVASLVFVHLCLTETRGRTFQDIQSNLSVKKKPEMTTIEKSDGSVAHFDGRTS	482
BTST83	MMSSIIEVRLHLFVMISFCYFXXGLGSMTVGLMVGWPAAAFPKILRHETPYHLSIFNEAFIISCMNIGSI<br>VGTVPASLLMDRIGRRASFLLFSLFAVASWIFVAYAPTVEMLYCGRLLGGVFVGAYLTILPSYLSETLEPD<br>MRGFLGTSSTLLNTLGTLMAYGLGPRVSFIDLSLISCCVAIVFIISLIFMPETPYLLVMRKDYAGARRTLA<br>WLRGTSESDVTVELTTIQNFIETEKAKASLTTSDLFFDERYKWPFVSCVGLLLLQKSSGYFTVIGNQTIIL<br>PHHAWIFYSEDSTLIIGLILVIMSIVAALLMDALGRKVLLQISNVGQASAMLVVGAWYYLSAEQRTELE<br>AYNYMPLLAVFAYVVAFSMGLGPVPHIYIGEVLPPLVKGRATGLLVTLAALFVVSVNEIFAAVTTFADM<br>YVNFLFFGVCSLVGIYYVNGWVIETRGKTLPEIQEEFRHRRTMKDGYYILD	472
BTST84	MFFKISKGIRRQLLAAFCCCIGAMTLGAIAGWSAAAFPKIRNNELEFRLSLFQEAWVINAFYIGIMMG<br>PLPAGIMMDAIGRKSTLLFFSTFAITNWTLVTLAYHEHMLYLARFCAGLWAGSVTTVVPAFLAEVLQP<br>NVRGSLGTMYFIMYFAGNLYEYIIGPYVTYFTFGISSGLLCFVFATSFVFIPETPYYYIMKGKRKKAEASL<br>RWLRGDEDVSAELESIQTYVKTFMKRRGRFKELILNENYRAAFINVQAVYFIQKLCGMFTVLAYLTLII<br>PSQVGPLSPEHCTLITGVVLWLSVFVATSLIDRVGRKPLFVISNIGIIITMTITGVWYFLNVHSDMDLSST<br>TWIPFSGLLLYGVSFCLGVGPIASLYQGEVLPSNIKARASTVTAIISAFASILNTTLFAICASYIGMYINFFL<br>FALTSVFALYFAQYHFIETKGKTLQEIQEELRKKPGFSRETSVNSNYSTVSSLPAIYTVPIARTHSLEKSAI<br>ANSLAKNA	500
BTST85	MSRFINRGVARQLAVAFTAGGSCFIIGALMGWPAPTLKKLRAADTPIRLSVLEESWVVNALYLTTMVS<br>PFMCGALMNSFGRKLTLLALTVFPTLSWILVFFSRSGAMLIAARFLAGFWVGGCSTVVPIYVAEIAEPA<br>VRGVVGTFTAVSTMLGIISAYVIGPCVSVYTMAAIYVVTPVLFFALFSLCPESPYFFVMRDQHVAAAAA<br>LTWLRARDSVTAELAAIQGSVERDAQTRQGCLRKLFSLVSVSANRKAFVTVEFMMVLQRMSGFSCLM<br>AYSSVILPSKVGPFTSDNCTLIMGIVWLGSALICSVLVDRVGRKPLLYFSSIGIFVSMLPTSLWYYLDRETS<br>TDVSGANWVPLAGVLIFGLTFTMGLGAIPSIYQGEMFSSSLKGIGSALTVGVCAGSSALSVSVFAVLVKF<br>VGLYAPFLLFAAVGPATFLFVYYFVMETRGKSLQAIQDELRGEELDR	462
BTST86	MGDDSSEKPPDRISKPLMAAVASYACQFQLGAILGQSSNMLPQLQAADSPIQIDYDSATWIASMDVLG<br>TPISCLLCGPLTDKMGRKATIRLFLLLSAVGHAIVGVASDVTEILIGRFCLGIAAGFAFPSIVYISEISSVEH<br>RTPLLAINTISSSFGLLYVFIVGAFISWDIISLMTSLISVISLVYAFFIPESPAWLFQNHRLNDAIDSIKWLKG<br>DDCDMTQELKQLKDACTEEPKGTGTIFRHFTGVTVVKPFFILLVFAFLQNGTGFYILLHYSINFISEFKI<br>DFDPRYISIGLAVVRLSVCIMASYFLSRVNRKTAGMVSGTGMVVVLGGTLVAMYFMMGDATMSTGYS<br>VIVTVGLLAFIFVCGLGAHPLPWIMIYELYPLHVRGTMCGVSNAINYVFIFIFIKMYYVLILNLQIHGTVI<br>LFAAFSAAFAAYSFLILPETQGKSLVEIEQGFLPKKQRQNGSA	466
BTST87	MIDKVLNAGQFFEKEGNQHKNACRSLLSQVVATIVLGGLVFDIGMMTTMPTLVIGALHKNSAGELK<br>MNDDQASWFGSIIFFAHPMGALISGYLQERFGRRGSMILVNVPVLAAWLTLHLADSVYQLHLVSVLM<br>GLCVGFCEAPLHSYIGEVAEPHLRGTISTLVCIAGHTGGVLLHVLGYLAQWRTTALFCGAVPAITFFA<br>MTQIPESPTWLILNSRLKEAQKALGWVRGWVEAEVVHEEFQRLLEHAAVAPKSRRVSTFEGQKYEMV<br>PLTEDGTPQKSTQETESFLRIKCRELSDQKMFRPLRMVFIVFIFSFATQLMGMRPFLVNIFNEFGLQIDSQ<br>LVVALTRFSLLVGAILNVTLLRRFGKRKLTLLCQAAATVSIILLGVYCSIFDETNRNASLSWIPISLMTSVY<br>FFIGFSLTILPWQLCAEVFPIRGRGAAQGLSAGWSYYVRFGMSVTYLYLEKWIRLSGVFYLYGVISVIGF<br>WYYWRYLPETEGKSLEQIESYFTDNHDKTEKFSRVKSK	519
BTST88	MREIDESGGTQDGQINEQKSSKKHKTDLRSASAQILATLIQDWLLLGIGLTLGVPTLVVGALYRNPAST<br>FTLDDDQASWIGSIPFICNIIGSLASGPFQEQFGRKGSMILVNIPFFCAWLLLFYARSVASLYAASAIMGL<br>CAGFSEAPLHSYCGEIGEPHLRGMLSTMSTSAAIMGSLLIYVFAYFFEWRGAALISSAFPVITFISMTQIP<br>ESPTWLVMRGRLEDAQRSLCWLRGWVEPDQVRDEFEALVNYTKQKVASLETAHLNILERPAIEKCNV<br>FTAHLKGLTAKNLLRPLRMECIVIANSYTTGFAGSKPYQIQIFRQLGYGDFAKRILILGHLIFFVGAMGN<br>LILLPYFGKRKLALFAFGLSFSCLFGIGTFGIFRMELIQIPGLFWLPLILILVFKFTLGLSIMPLPWQLLCEV<br>FPPIGRGTASGISSAFGNLISFGMTKSFLYLKAWLDLPGVIYLYVACAFLGWLYFYFYLPETEGKTLEQIE<br>SYFTENHDRKEKFSIGKSGRKN	514
BTST89	MEKLNESYGMQQVTIDTENTEIKHKQNLRSTSAQLLATLAQNWLLLDIGLTFGVPPLVLGALHLNTG<br>SGLSLNSSETSWLGSLPSICHLIGSLASGLFQEQFGRKGSMVLVNIPFFCSWLLLYHAESVLALYIAFITM<br>GLCAGFCEAPLHSYCGEIAEPRLRGTLSTLCVAATILGAVFMYLLGYFFEWRVAALISSGVPVITFLLTT	474

	QIPESPTFLLMRGKTEEARKALCWLRGWADPDEVKEEFQALINYTKQRVASLESSGGNKNASLKFKKP<br>NDLKAQLSELTSDRILIPFRLEFFVTLNAGITSFVGFQPYQIIVYKELGYPNFGKEILVRYTRIFLGESDTV<br>MKKRHDTKVRSSLTNVTDEQLLGETKENQKKLLSAIRGRGTASGISTAWAYLLTFSLTKTFLVMVAWL<br>NLGNVFYLYGTCGVLGWIYFYFSLPETEGKSLEQIESYFTKNHDRKEKFSVGKSGHLK	
BTST90	MCNKTEPSKSLDVPGNDAKPSFQYKNPGKSAFAQILAIIMQNWLLIEIGLEMAMPVIVLGALHNNPA<br>AALNLNDDEASWFGSIPDFFHPIGSLTSGLLQEKFGRKGAVMMINIPIFIGWMTLYFAKSVYMLYAVSV<br>IMGLCTGLAEAPLHAYIGEIGEPRMRGTLSTISTSCCIIGVSLMYLLGYLFEWKTVALISSSCSVITFLMM<br>TQLPESPTWLIVRGRLDEARKSLCWLRGWVTADEAEPEFQALVNYTRNSAGLSRQDSTNSTVDDDGP<br>LVRKDGFLTQQFKELMNKKTFRPLRMVFTLFIICFFGYVGGIRPYFINELKKLESPIDPKLFLTMGTGWL<br>FLGAMINVVFLRRFGKRRIAIFSHALGGVAIAGVGIYATFLQGLTQYPLRVWVPIALWTVVNFLNGLST<br>VTLPWQIVCEVFPPLSRGTATGLSAAWAHLVLSVHVKLYLYVEAWIGFNGIMYLYGICTLLGSTYHYFC<br>LPETEGKSLEQIESYFTKKHDKKEKFSMGKSVQKGDP	519
BTST91	MFSKFMPFKSKSMSMETGNDRAIKPLICVALLIVFLAGCILGRSEDPRDEDDPYNRLKNHHDPKTFGD<br>ILYEYIRDIVKVPVIATVFFCWVCGSFADEHGRVGAMQLFFMLSGIGFGFLVYAQEYDFSILGTFILGAA<br>LGCSIPAPIYIAELCPVAYRSFFLGLVPVALSLGMFTVDVIELRGAEDTAWKSLCCFSGIGFLLSLFLHEAP<br>EWLVMRNRPDAAIESLKWLKETSVDVDVDLRKLQETSMAANHRSDTTLEMLTDKRVWKPFAMLLG<br>LALFQHLCGFYILIFYAPYLVNQYRTNIYWFSSYTGTDFLLLVATSAALVFHANLPRRTVAGLSGIGSSA<br>ALLGLFLHAHLFVAPQDLLDPTPDKDMLVPVFFFTLYIFSAVMGIYTLPWILMFEVFPLRHRGILCGLSF<br>STLYLGLFAFESRLNNYLLTGMDLQSLLCFFGTCALGFALFARSCLVETHKKTFEEIERGFTKERIFLPID<br>EKM	489
BTST92	MLKNPTSRLKLMALLEGDSIIHSDRIGYSRVPSSDIDKDAAKSHIEAETDGSTGRKISRFRSALPQVLSVT<br>AKNFILLDLGMTLAFSTIVVPVLLNKKDPRGLSFNESQATWFASLPMLCQPLGSALSGLISGPLGRKKS<br>LMLVNIPQILGWLMLHSATSVEIMYLAAAIQGLGSGFMDAPVLTYVGEICEPSLRGILISYSLQFCSAGF<br>FLQCLLGSVTTWRNVAFISLFFPATAFLCISQIPETPMWLLSKGRTRDAEKALCWLRGWVSPEDVAEEF<br>SRLVEASNNAQYRHHAETKKPETNSHQGNSQSIVTCPSSRPPIPDPLVELHSPKLSFREKAKDLLRPEIL<br>RPMLVIMTMNFFYLGSGFPAFKTYMVLLFQRIRVPMNANWASVCVSSAIIIGHLVLMLAVKWLGKRRI<br>ALISIFCVAFFDLAIAVHVNFQTAIEDAFGASANWFLFSYFIILTLTVSFGLGPVPWMLMSEIFPFRGRSF<br>ASGICAALYYITSSLIAKTFLSILNLLGVPGSYCLFGTVGMLGFIYAYLYLPETEGKTLEDIEDIYKHSHRQ<br>RR	562
BTST93	MNKEEEDEKITWSCWLRTLFACSGAMLCFIFSGVTEAQSSTLLPQLKAKDSIIHVTPEEETWIASLGILM<br>APIASVVVGPTIDLIGRKKGLLFFYLDMGVGFTIIACATEVWHIYVGRCVCSFAIGMEVAAVVYFAETC<br>TKKQRSVLLSIISASFTFGVSLTYFVGGYLPWNVASGVFALGCFLYFLIQLLAPESPAWLYKKGDIDASKR<br>SLQRLGRSPSGILRELEMLRLSSKEQSEKFQFKVFLEPTVWKPFLIMCIFHILLNLSGVFHIMYYTLDFIER<br>LGTSYDPLTVSIIISVTRVISCSTVGIYSTAYVGRKPATIVSSILMTLSFLSAGIYEYVFRDTPVGQKPYEWV<br>PIVLLISNLVSGILAVAILPWLISGELFPLQIRGSMNGAVYVFGTSLMFVSIKLYAVCLEVFQMWGLLLVY<br>SLGSFLAILFGIFVLPETQNKTLLEVERGFLPKNRRDIAPSPNAEATKNCGDTSQSEAGLERR	489
BTST94	MDEEKEDEKISWSCWLRTIFACSGAMMIFIFSGVTETMMIFIFSGVTETLSSTLLPQLKEKDSIIHATPEEE<br>TWIASLGILMAPIASLVVGPTIDLIGRKKGLLFFYLDMGVGFSIIACATEVWHICVGRCVCSFAIGMEVA<br>AVVYIAETCTKKQRSVLLSILTVSFTFGVSLTYFVGGYLPWKVASGVFATGCYLYFFIQLFVPESPAWLYK<br>KGDIDASKRSLQKLGRSPEGTLRELEILRLSAKELSEKFRFKVFLEPTVWKPFLIMSTFHLLQNLSGVFHI<br>MNYTLDFIQRLGTSYDPLTVSIIIAVSRVITCCTAGMYATASVGRKPATIVSSILMTLSFLGAGIYEYVFQD<br>TPVGQKPYEWVPIVLLIVNIISGSLAVSILPWLMSSELFPLQVRGSMNGAVYVVGTSLMFVSIKLYAVCL<br>EIFEIWGLLLVYTLGSFLAVLFGIFVLPETQNKTLLEVERGFLPKNRRNIASSPNAEATRNSEDAGQIEA<br>GSERSDESKE	506
BTST95	MERDDEDEKLSWSCWFRTMFACSGAMMLFVFTGVTEAQSAVLLPQLKERDSTIHTTLEEETWIASLGI<br>LLSPVSGVIVGPTIDAIGRKKGLLFFFINMGLGFGIIACATKIWHLYVGRCICSFAVGMEVAAIVYLAETC<br>TKKQRSMLLSMVAASFTTGCSITYVVGGYLPWNIASAIFALCCFLYFLVQLLAPESPAWLYKQGRIDAS<br>TRSLQKLGRSPEGIARELEMLRLSSKEQSEKFQLKVLLEPTVWKPFIILCIYHFLQCATGVFQILYYTLDFI<br>DRLGTSYNPLRVSIVISIVRVIANSTIGMYATAYVGRKPATVTSGILMTISFLGAGAYEYIYRTTPVGHRPC<br>EWVPIVLLIVNIVGGILGVCILPWLMSGEVFPLRVRGSMSGAVFVVGSAIMFVSVKIYAAAMEVLAMW<br>GMLFVYAAASFLAVLLGAFFLPETQNKTLYEIERGFLPRDRGDSRVQATEKSEIENETTVS	481
BTST96	MNEGTANLLLSQMKGATSLIHLSQDQETWVASLGILSAPIAAILIGPFIDAFGRKRGVLLFYLNMGLG<br>WAVIASAREVTQIYIGRIICAFGEGFQACAVVYLTEICTKEQRSVVLACLIALFSGGVLFVSVVNTCLPW<br>PMACSAFSLASFALAGAECFVPESPAWLFSQGEEAAAVRNLQKLGRSKAGVLLEIDALKERESCTEVL<br>SWRTFLRPTVWKPFLILAVFHLLQFSTGFYDMIYYQVDYLERLGTKYDPIALSVAFSTVRFLSNATIGIYF<br>RSLDRKFSTTVSGLCMTVPLLGAGIYELKYRDTPPLEKPFQWLLLFCIFAQLVAGNLAVTCLPWSMGA<br>ELYPLNVRGIMSGATLCVAYSIFFTYVKLYHVAMGALKVYGLLCLGLLF	394
BTST97	MTRINAHGVKKKDSFFGPFVASIPAFVTQVLAGVLEGHAAVLLPQLNDASSPVSISSDDAPWIVSLGFV<br>LTPFIAVIRKPLMDTFGRKCNLYLFFVLSTFGFLSVSVASAPAHLYLGRVISSAAYGLSPDSFVYVSEICAD<br>GQRSSFLFFLSLMRPAGLLMVYSLSTILEWGICSMVFVVTSHIGLLFVFHVPQSPVWLVQQSRLEEAATS<br>LKWLRSDHDVIDKEIAAMKISTPDEEFQQRSGLVAFFNPSIFKPLAALVAYSVLQHATGLLVVLSFALDF<br>FGSLSSPFNPAYVIVCIAFYRIFLTAFWVYLSSKFDKVFLTTTAAFGAGCLVLSVCVMQVAYPDVVGRNE	468

	SLQLLQLSLCASYTLIGGGIDFHNLPVDLRTVFPPDVLGTVRGFVKFLGGMFLFLSMLSYPFLVRVFGLS<br>WVLFFFGISCFSFGLLVKSLLPKFKGKTRTDDTEGTHSTRLQLTTYL	
BTST98	MSRGPYQMTGHALQPLNPTSNGLGTHTAGTMVNLPRGKSQYLSQVLAAVAISLGPLAAGLGKGYSS<br>PAIASLQGKQSWEAGHGAGAYRGHGMGHRGNYTLLTVSPQEASWVASLSLLGALFGALVGGLAMK<br>FGRKNVLLIASLPFSASWLVTVYAESVQTMFATSFVGGFCCAVVLMVSQVYISEISDPDIRGFLSAVLKIF<br>SHIGTLLSLTLGAYLDWRELAMIISGAPLLLFVSMLYMPETPSFLVLSGREPDAVRALRFLRGNDTDITR<br>ELITIRNNILTASTHQYTYRGLAHAAARLAHPILITCGLMFFQRFSGANAFQFYSVTIFSQTFNGMNPH<br>GGAIVVGFVQLLASLLSGLLIDTIGRLPLLIASSVFMSIALAGFGSFVYYEQLSRHNSYVHVQHLPPGVA<br>PPGISATYDWIPLLCVLVFTVSFSMGISPISWLLIGELFPLEYRGLGSALATSFSYACAFIGVKTYVDFTQT<br>LGLHGAFWLYAAFSLAGLCFIVCFVPETKGRDLDELDSRYI	524
BTST99	MISSGKTQSPNERPYQYEYTALGTQDVEKSGEHGTGVSGNRRKVNRFRSAAPQILAVTAKNLVLLDL<br>GMTMAFSTIVVPVLLDPNNKDPNGLSFTEDQATWFASIPMVFQPLGSALSGLISAPLGRKRSLMLVNIP<br>QIIGWLMLYSSSSVNIMYLAAAIQGLGAGFMDAPIFTYVGEICEPSLRGVLISYSLQFCSVGFFLQCLLGS<br>LTTWRHVAFISMLFPTLAFLAISQIPETPMWLLSKNRMKEAEKALCWLRGWVSKEEVAEEFAQLVQYS<br>KNSKYKSDDDKKKLQMDLISTAKQPCGGCTRPPIVPCDSNTGDDDYAKLKLHEKVKDLLRPEILKPM<br>SIIIIVNFLYFTSGFPGFKTYMVLLFQRVHSPIDPNWASVFVSTSIILIHIAQMVAVKTIGKRWMTLISSFGA<br>AVAGLAIGVHMSFQGFFDETFGDLSNWLLFTYFEILTLATVIGLGPVPWMLMSEIFPFRGRSFASGFCA<br>AIYYAASFFAAKTYLSTLNLFGVAGTYYIFGTISALGLVYVYLYLPETEGLTLEEVEDIYRPKKRSEVKNL	555
BTST100	MDSTRGLRRQVTACIIANQGLFLIGINLGWSSAVNEHLLSGVLGYKYTQDQLSWAVSLLDLGTVFAPL<br>PTGYLMNKIGRKFTFLLIASLFTLSWCLKVISVQPGFLYAAQILAGVARGVGLTVTPMYSGEIAETGLH<br>GMLSTIFKLMFYSGMLLMIIVAPYMNYTTISYMGLTFSLLFFFSLFYIPDTPYYYCAVKKEREAFQSLKW<br>LRNQDKTENASVLNKELAMIKVAIEKVMGEDSGFRGLIMKPSNRRALFIVLGLFILQRMIGLNTIIGYG<br>SITLPKGHPFITPQTGMISFVVALFISSALIALFIDRIGTKPLLISSSIGCGFCTSVIAVYYWCDRTNGKAAV<br>AGFFWVPYLFFVLEAFVFSIGVGVVPTVYLSQLFPINVVGQASAASVIVASFVTFVINKAYFYVGVQFGI<br>FMMYVFFSMSAFGCAAFTHFFAIETRKKRDAEIVPVGSVIETASARK	466
BTST101	MPLRNVPVSILPFVYGLVQTWPNLAIEEILKGEAGFAVDPEDIAVIVSMVTLGEFLMPILFGFVLVRLGR<br>KTNMLLNALIYAVAWALIIFARSPFWLIAANFAAGLGCGIILIIGPIYMAEIADSKIRGALISVYITVIAIG<br>QIFMTSVGIFISYFDMNLIALILSIVAFFCILVIAVESPSWHLIDNDEVKAEESFNYYWNTGEEADRPQAG<br>EALAELKETVKLEMESSSSYLELFRTPSNIRASIIVITLSFFQGASGIVAVLTYGSTTLPRYDGFWKPYPTM<br>ALVSALNLVFNLVSIALIDKLGRRPLSIVSNAGDALGTGIVAVYFFLEQNTEWDTTNIKWLPYIGMILYIS<br>SYGGAMAVIPHILVGELFPTNVR	379
BTST102	MTTMVIGALHNSASEELSMNDDQASWFGSLPDICHPVSSLLSGYVQEAVGRKTAMIMVTIPCFVAWIT<br>LYFAQSINTLYIVSIIMGLCTGLTEAPLHSYIGEIAEPHLRGTLSSISTSAALIGMFMMYVFTYFFYWRTVA<br>LICSACPVITFTVMTQIPESPTWLVVRNRLEEAKKALCWLRGWVKPDEVEEEFQALVKHAKKSVGLN<br>QAGDDKPRTKIAFLKMQLTEMTRKKVLLPFRQICIVFFICSLAYFCAIRPYLIGELQKLDTPIDAKLILIYS<br>QVLLFIGAMMNVMFLRRFGKRKIAIFCNTVIAISMFGLGIHYAYLKGSKQFPLLAWLPVVFWLSISFFG<br>GFGPALLAWQLVSELFPIIGRGLATGISAAFSKLMGAAEVKSYLYIEAWVDLSGVMYLYGTATILGTLYL<br>YFYLPETEGKSLEQIEVYFTENYDRKEKFSIGKRVKLEEKNPS	461
BTST103	MGPLTDAPESHSIKNGNAASSNRPRHSTRSTCAQVLATLIQNWLLIEIGLDIAMTTMVIGALHNSASE<br>ELSMNDDQASWFGSLPDICHPVSSLLSGYVQEAVGRKTAMIMVTIPCFVAWITLYFAQSINTLYIVSIIM<br>GLCTGLTEAPLHSYIGEIAEPHLRGTLSSISTSAALIGMFMMYVFTYFFYWRTVALICSACPVITFTVMT<br>QIPESPTWLVVRNRLEEAKKALCWLRGWVKPDEVEEEFQALVKHAKKSVGLNQAGDDKPRTKIAFL<br>KMQLTEMTRKKVLLPFRQICIVFFICSLAYFCAIRPYLIGELQKLDTPIDAKLILIYSQVLLFIGAMMNVM<br>FLRRFGKRKIAIFCNTVIAISMFGLGIHYAYLKGSKQFPLLAWLPVVFWLSISFFGGFGPALLAWQLVSEL<br>FPIIGRGLATGISAAFSKLMGAAEVKSYLYIEAWVDLSGVMYLYGTATILGTLYLYFYLPETEGKSLEQIE<br>VYFTENYDRKEKFSIGKRVKLEEKNPS	514
BTST104	MQNDTAETHRHMHTRHKIFSRSAFAQILAILMQNCLLIEVGLDTAMPTMVIGALHKNPSETLNMND<br>DEASWFVSIPAIFYPLSSLTSGYVQELLGRKKAMLLVTIPTFAAWMILYFAQSIYTLYAASTLMALCNGLT<br>EAALHSYVGEIGEPHLRGTLSTISISAMLFGGLMMFVLGYFFDWRTVILICGAYPIITFMVISQLPESPTW<br>LIVKNRKEDAKRALCWLRGWVKPEEVEQEFQAHLSYAERSVSSSLEKLVSNGSLENLDYIKIQFTEMA<br>KERVLRPLRMICIMFIICITAYCWAIRPYQIGELKKMGSPVDPKLVLIGAQVFIFVGLMMNVLFLRRFGK<br>RRIAIFSCSIIAFCMFGIGFHHANLKGRETTFLAWLPVILWLTINCFAGFSAALLAWQLVSEVFPIVGRGL<br>ASGVSAAWSSIVVFVMIKSYLYIEVWIGLSGVMYMYGTITALGVLYLYFYVPETEGKTLEQIESYFTDNH<br>DPEEKFSIGKSK	499
BTST105	MTNEMESSENQAESQTEDLIPKPKEIKYKNAGRSTFSQIVAMLVLACLLVDFGLELIIPTIVIGALHKNP<br>DEALNLTDEQASWFGSILYFAHPIGALISGFLQELLGRKRSLLLVNIPMLVAWSTLYLASSVYQLYFVSA<br>ALGLCIGFCEAPLHSYIGEMSEPHVRGTLSAMGTASCLMGMLIMYLIGYLVHWRTAALISSFVPVITFL<br>AIAQIPESPTWLVMNGREKEAQKALGWLRGWLKPEEVQEEFQRLLEYTDTKPKSKRFRGSQREKYEM<br>VPTSENGVPQPLREHDESYWRKKFREITNKKLYLPLRMVFIVFFFGIATQLAAMRPFMVGVLIQFGLTV<br>DNYLVLVLISFFYFVGAMMNVIFVRRLGKRRLTLYCQAIATLSILLLGVYLAYLTGPAKVRAIDWIPISLF<br>VSLFFASGSSIALIAWQLCAEVFPVEGRGTAQGLVAAWAYLVNFVMSKSYLYLERLVQLKGVFYFYGAL<br>SALGFFYYWRYLPETEGKSLDQIETYFTENCDEKDKFTKRKNNRG	530

BTST106	MTENLPQSMETRQETSNPSTFRQLLPQVLACSAKSVLYLSLGMLVGLPTLLIPDVTDPSNLNELFLDND<br>QASWYGSLTYIFQPLGSLASGALLQSVGCKKLMIMVNIPQFVSWIMTYYASSALVLYISSALVGLVVGL<br>MEAPTCRYISEITHPNYRGVLTSYSTSFVTVGFLLVYALGLVTNWRNVALISASTPVLAIIVLLMIPETPI<br>WLMSKGRSEEALESLQWLRGWTTKEAVQEEYTKLQFYAKKQQGKTIHAYGKEVDIADEKPTVLNGA<br>SSGGHLEVEEVEERRGLKEKIRELTCKEMLVPLGKCIVLFFVSICSGLLSLRPYFVQVFEEFDLPTDGLTT<br>SVLISVIAIVGNIVCMLIINRVKKRPLIIFSLISTALCLILLALFLMAPTNPHNNASLRWWSLILFLIVNFV<br>NNLGIYPISWTYLSEILPYRGRGIATAIGSSFFYIVIAVGVKTYPSLEQQIGLDGIFLLYAVISLAGAYFTYFS<br>LPETEGKFLSDIETHGKDKKIQVPSASLRP	522
BTST107	MRCFGSARGLTFFLSYTILASMLGMFQFGYNTGVINAPEKNIKDFMKDVYKSKYSEDISEETVQFLYSF<br>AVSIFAIGGMIGGFSGGLIANKFGRKGGLLLNSFVGIAGASLMGFAKFFHSYEMIFIGRFIIGVTCGLNTS<br>LVPMYISEIAPLNLRGGLGTVNQLAVTTGLLISQILGIEQILGTDDGWPLLLGLAICPAVLQLILLPVCPE<br>SPRYLLITKQWEEEARKALRRLRASNQIEEDIEEMRAEQRAQQAEATISMTQLLCSRTLRPPLIIGVVM<br>QLSQQLSGINAVFYYSTGLFVSSGLSEETAKFMTPGIGVIMVTMTIITMPLMDRLGRRTLHLYGLGGMFI<br>FSIFITISFLIKEMIDWMSYLSVISTLMFVVFFAVGPGSIPWMITAELFSQGPRPAAMSIAVLVNWIANFVV<br>GIGFLPMKTALENYTFLPFSVFLAIFWIFTYKKVPETKNKTFEEILALFSKPGDTPPTRGQPQANPAFEET<br>PLNTTKAFTGSTSTSLLNCVDQRLPPSERAALMVAEEKPLPDTSSSSSSSVLPASLYDADGTMSPPVNPG<br>SRNQMQYGAASEHCVRDLSQPSRPPPPLPPPRSFLPNSAV	603
BTST108	MREIDESGGTQDGQINEQKSSKKHKTDLRSASAQILATLIQDWLLLGIGLTLGVPTLVVGALYRNPAST<br>FTLDDDQASWIGSIPFICNIIGSLASGPFQEQFGRKGSMILVNIPFFCAWLLLFYARSVASLYAASAIMGL<br>CAGFSEAPLHSYCGEIGEPHLRGMLSTMSTSAAIMGSLLIYVFAYFFEWRGAALISSAFPVITFISMTQIP<br>ESPTWLVMRGRLEDAQRSLCWLRGWVEPDQVRDEFEALVNYTKQKVASLETAHLNILERPAIEKCNV<br>FTAHLKGLTAKNLLRPLRMECIVIANSYTTGFAGSKPYQIQIFRQLGYGDFAKRILILGHLIFFVGAMGN<br>LILLPYFGKRKLALFAFGLSFSCLFGIGTFGIFRMELIQIPGLFWLPLILILVFKFTLGLSIMPLPWQLLCEV<br>FPPIGRGTASGISSAFGNLISFGMTKSFLYLKAWLDLPGVIYLYVACAFLGWLYFYFYLPETEGKTLEQIE<br>SYFTENHDRKEKFSIGKSGRKN	514
BTST109	MGTAVADIYHDSVSNSGKSRRFDNQNNNINMFPRKENPAKPFRKALPQILAVTAKNLLVITYGMTLG<br>LPSIAIPALQEKRNSTMNDGHPHDQLTLDEAQISIFSSLNLICVPIGCLLSGVLTQPFGRKRCMIFLNLPF<br>IVAFLSFCYSSSVPMLYTALIISGLSGGLLEAPVLTYVAEITEPHLRGMLSATASMTTILGTVSQLLLGNFF<br>TWRTVALIDLFFPVAAIVALCFVPESPHWLISKGRITAAEKALCWLRGWVEPDSVQSELSLLQKSHDQS<br>LNRSSPTSSMYIMYMKRTFLIPFLIITMSYFIGHFGGMTVIQTYVVSIFEDLGAPIDKYFAAMLFGLVELA<br>GALTCVGLIHYTGKRPLTMFSTVGCGICLFGVATCAYMGYGDASSKSQYSSFSTMLLLATAYLSHASIRL<br>LPWIMIGEIYPAEIKGMASGASASVSYIFAFTANISYDTMIRYLSLHGTMYFYSAISLLGSLFLYYCLPETE<br>NRTLHEIENHFANKEHLFKKKISKTDILHRKDEKPEENEDESTEADCILETS	544
BTST110	MGKDEDEEKLSWGCWLRTAFACSGAMMAFVFNGVTEGQSAVLLPQLKEKESFIHITSEEETWIASLGI<br>LLSPVSALLIGPITDAFGRKLGLLFIHIFMGLGFAVIACATQVWHIYLGRCICSFALGLEVVSVVYMTET<br>CAKRQRSFLLSTISPAFTLGVVVAYVIGGYLPWNIASAIFALSSFVYFVVQLLAPESPAWLFKRGRIDAA<br>AWSLRKLGRSPSGIDHELQLLKLASSEESESFHLGIFLDPTVWKPFLILSLFHLVQCATGIYHIVYYTLDF<br>VTRLGTTYDPLTVSIVISVVRVISNCTIGMYFTSYVSRRFSTILSALLMTVSSGAAGVSSSGIIGVTTLPWM<br>MSGEVFPLRVRGAMSGAVFGVGAGSMFVFIKIYEDCLALLNIWGLLFGFAIASFLTALLGIFLLPETLNK<br>TLYEIEQGFMPKEKRSNGEESTLPAEAVS	450
BTST111	MGGEDQKPRASKQSANGRLLFAISAAALGSAFQHGYNTGVVNAPQSLIESWISDVLRNRSGAGAEY<br>KPEPSQVTMIWSIAVSIFCVGGMIGGSLTGLIAEKLGRKYGLLYNNILVLAGCLLQQHSKNFGSYEMFI<br>AGRFFIGVNCGLNAGLVPMYLSEISPMNLRGAVGTVYQLVVTISILISQIFGLKSVFGTAENWPLLFEIA<br>LLPSIFQVITLPFCPESPRHTLLHHGLELQAQKDLSWFRGTIEVHDEMEEMKNEYEAMKLTPQVTIRE<br>MLSNAQLRIPLFIAAMVMVCQQLSGINAVMFFSTKIFKMAQLSDEAAQYSTLGMGSMNVLMTLISLV<br>LVEKAGRKTLLLIGFSGMFVDTVLLTICLAFVEKSIVISYFCILLVIVFVVMFAVGPGSIPWFLVSELFNQS<br>ARPTAASIAVAVNWTANFMVGLGFLPLQEALGSNVFVIFAVLLGLFVLFVWKKVPETKNKTMEEISSM<br>FRQISYQ	487
BTST112	MGPLTDAPESHSIKNGNAASSNRPRHSTRSTCAQILATLIQNWLLIEIGLDIAMTTMVIGALHNSASEE<br>LSMNDDQASWFGSLPDICHPVSSLLSGYVQEAVGRKTAMIMVTIPCFVAWITLHFAQSINTLYIVSIIMG<br>LCTGLTEAPLHSYIGEIAEPHLRGTLSSISTSAALIGMFMMYVFTYFFYWRTVALICSACPVITFTVMTQI<br>PESPTWLVVRNRLDEAKKALCWLRGWVKPAEVEEEFQALVKHAEKSVGLNQAGDDKPRTKIAFLK<br>MQLTEMTRRKVLLPFRQICIVFFICSLAYFCAIRPYLIGELQKLDTPIDAKLILIYSQVLLFIGAMMNVMF<br>LRRFGKRKIAIFCNTVIAISMFGLGIHYAYLKGSKQFPLLAWLPVVFWLSISFFGGFGPALLAWQLVSELF<br>PIIGRGLATGISAAFSKLMGAAEVKSYLYIEAWVDLSGVMYLYGTATILGTLYLYFYLPETEGKSLEQIEV<br>YFTENYDRKEKFSIGKRVKLEEKNPS	514
BTST113	MCSNPDMTSSSSMQNETAETHPHMHARHKMFSRSAFAQILAILMQNCLLIEVGLDTAMPTMVIGAL<br>HKNPSETLNMNDDEASWFVSIPAIFYPLSSLTSGYVQELLGRKKAMLLVTIPTFAAWMILYFAQSIYTLY<br>AASTLMALCNGLTEAAIHSYVGEIGEPHLRGTLSTISISAMLFGGLLMFVLGYFFDWRTVILICGAYPIIT<br>FMVISQLPESPTWLIVKNRKEDAKRALCWLRGWVKPEEVEQEFQAHLSHAERSVSSSLEKLVSNGSLE<br>NLDYIKIQFTEMAKERVLRPLRMICIMFIICITAYCWAIRPYQIGGLKKMGSPVDPKLVLIGAQVFIFVGL<br>MMNVLFLRRFGKRRIAIFSCSIIAFCMFGIGFHHANLKGRGTNFLAWLPVILWLTINCFAGFSAALLAW<br>QLVSEVFPIVGRGLASGVSAAWSSIVVFVMIKSYLYIEVWIGLSGVMYMYGTITALGVLYLYFYVPETEG	511

	KTLEQIESYFTDNHDPEEKFSIGKSK	
BTST114	MTNEMESSENQAESQTEDLIPKPKEIKYKNAGRSTFSQIVAMLVLACLLVDFGLELIIPTIVIGALHKNP<br>DEALNLTDEQASWFGSILYFAHPIGALISGFLQELLGRKRSLLLVNIPMLVAWSTLYLASSVYQLYFVSA<br>ALGLCIGFCEAPLHSYIGEMSEPHVRGTLSAMGTASCLMGMLIMYLIGYLVHWRTAALISSFVPVITFL<br>AIAQIPESPTWLVMNGREKEAQKALGWLRGWLKPEEVQEEFQRLLEYTDTKPKSKRFRGSQREKYEM<br>VPTSENGVPQPLREHDESYWRKKFREITNKKLYLPLRMVFIVFFFGIATQLAAMRPFMVGVLIQFGLTV<br>DNYLVLVLISFFYFVGAMMNVIFVRRLGKRRLTLYCQAIATLSILLLGVYLAYLTGPAKVRAIDWIPISLF<br>VSLFFASGSSIALIAWQLCAEVFPVEGRGTAQGLVAAWAYLVNFVMSKSYLYLERLVQLKGVFYFYGAL<br>SALGFFYYWRYLPETEGKSLDQIETYFTENCDEKDKFTKRKNNRG	530
BTST115	MSLEAEKLEGLNTQNEAVTIIKSRYNYSRRSAFAQVLATLIQNWLLIEIGLDTAMTTMVIGALHLNSEE<br>ALSMNDEQASWFGSLPFICHPLASLLLSGYFQDKFGRRTTMILVTIPTFIAWVSLYFAQSMYVLYLVSAV<br>TGMCTGLTEAQSPTWLIVKNRYEDARKSLCWLRGWVDPSEVEEEFQALVSHARNSVQKNKAAQSV<br>GDGLIKKDSYLKTQFKEMTSKRVLLPLRLILIVFVFREIFIGAMMNVVCLRRLGKRKIAIFANGIVAICIL<br>GTGIYCSFLQDSTRFPQAAWLPVIFWLMLSLFCGFSATLLPWQLVCEIFPIVGRGLATGITAGTKYLIQS<br>AMVKSYLFIETYIGLSGMMYLYGTGAVLGVIHLYFCLPETEGKTLQQIESY	396
BTST116	MAENPCETCRHSYKNGRRSMMSQVVVMLVLSGLFMNTGMQATMPTLVIGALHDNSAAHLELNDD<br>EASWFGSILSFSHPIGALTSGLLQERLGRKGSMLFVNIPTLAAWTILFRATSVYQLYLVVVLMGVSIGLM<br>EAPLHSYIGEVGEPHFRGIMSTMATAATILGVLAMHVLGYLFPWRKVALISAAVPLISILCLTQIPESPT<br>WLILNGRAKDAQKALSWVRGWLRPEEVQKEFDELLHHTEVALKINRTSASDRETYRAVPAVENEALS<br>RQVEPVLTEENGSCLKIKCEELTDKKVYRPLRMVCIVFFFSSVTELSELRPFMVGIFKDLGFPIKNHPISV<br>LTAVFFFIGAMLHVVFLRRLGKRRLTLISQALATLSILLLGVYCTFFNKPNSSLVWIPISLMSCISFCGGFG<br>IALIPWQLCAEVFPLKGRGTAQGIAASWAYYMRFVMSKTHFYLERWIKLNGVFFLYGVIAVAAFLYYL<br>QYLPETEDKSLKHIETYFTENHDKTEKFCKPKHNRSHNVR	522
BTST117	MCHPSNPSKNVSIEDGPAEANVQYKYTRRSAFAQILATLIQNWLLIEIGLDWAMPTMVIGALHRNSEE<br>SFNMDDDEASWFGSIPSICHPLASLSSGYFQELLGRKSTMILVTIPTCAAWITLYFAQSVQTLYLVAGTM<br>GICTGLTEAPLHSYIGEIGEPHLRGSLSTLSQSAGFIGVFLMYLLCYFYDWRTVALICSACPIITFTSMTQI<br>PESPTWLIVKGRFEEAKKSLCWLRGWVKPCEVEEEFQTLVDHTKKSVKSIDSTQMVEDCASAKIDGYL<br>RMQFERLTSKKVLLPLGMVCIVFFFSVVAGFVGIRPYLIGELKKLGVPIDPKLILIAFQVLVFVGAMMNV<br>LLLRRFGKRRIALFSYSTHAFSILVLGIYCSSMESLEQFPQLAWLPVIFMLTLGFLTGFSSTLLPWQLVSEL<br>FPIVGRGLASGISAAWAYIVGFILVKSFLYTETWIGLSGVTYAYGAVSVIGVAYIYFYLPETEGKTLEQIEKY<br>FTKNHDRKEKFSIGKSTNNSGLEHSS	519
BTST118	MKSSEEQTEVSFEEPPDTSKIRYENSRRSTCSQVVVMMVLVGLLLDIGMQASMPTIVIGALHRNPSERL<br>SMNDEQASWFGSILSFSHPIGALISGFLQERFGRRGSMILVNIPTLAAWTTLHLADSIYQLYIVAATMGL<br>SIGFLEAPLHSYIGEVGEPHFRGTMSTMGTAAALLGVLTIHMLGYLVRWRTAALISTAVPLITIVCLTRIP<br>ESPTWLIMNGRIKDAQKALGWIRGWLRPEAVQKEFQQLLNHIEAAPKIHRTRSIDNETYQMVPTADC<br>EAESSLRPPEENKSYLRSKYEELTDKRLYRPLRMVFIVFFFTSATELSGMRPFMVGIFKDFGFAIDSQLLL<br>VFSIAFFFAGAMLNVVLLRRLGKRRLTLMCQAVATLCILLLGTYTTLFNKSNRIPSLVWIPVTLMSCINF<br>CGGFAITLIPWQLCAEVFPLKGRGTAQGLAAAWAYYVRFVMSKSHLYLERWIKLNGVFFLYGAVAIIA<br>FLYHLRYLPETEDKSLEKIESYFTEDHDEAEKFLKPKSSNKSR	529
BTST119	MCHPSNPSKNVSIEDGPAEANVQYKYTRRSAFAQILATLIQNWLLIEIGLDWAMPTMVIGALHRNSEE<br>SFNMDDDEASWFGSIPSICHPLASLSSGYFQELLGRKSTMILVTIPTCAAWITLYFAQSVQTLYLVAGTM<br>GICTGLTEAPLHSYIGEIGEPHLRGSLSTLSQSAGFIGVFLMYLLCYFYDWRTVALICSACPIITFTSMTQI<br>PESPTWLIVKGRFEEAKKSLCWLRGWVKPCEVEEEFQTLVDHTKKSVKSIDSTQMVEDCASAKIDGYL<br>RMQFERLTSKKVLLPLGMVCIVFFFSVVAGFVGIRPYLIGELKKLGVPIDPKLILIAFQVLVFVGAMMNV<br>LLLRRFGKRRIALFSYSTHAFSILVLGIYCSSMESLEQFPQLAWLPVIFMLTLGFLTGFSSTLLPWQLVSEL<br>FPIVGRGLASGISAAWAYIVGFILVKSFLYTETWIGLSGVTYAYGAVSVIGVAYIYFYLPETEGKTLEQIEKY<br>FTKNHDRKEKFSIGKSTNNSGLEHSS	519
BTST120	MWPWKKLESKDKLKSSKWLSGSIESLSEEELTAVYGAGARIHSSHHRPPPSALSSTCSSSTSVATNGSTV<br>ALLHSSQRKKSKNKYNVHKYNLYNKGIKMTSGAQKELNLLLTAPTVPEIIKSESEKDPKKDEANDGR<br>LQNSHEATFETLNSSGIIIKEKQDSSQSASIIPQVLASLSVSLGSLAVGFSSAYTSPALPSMTDATSILYGKV<br>SAEEMSWIGSIMPLAALFGGMAGGPLIESLGRRTTIISTAVPFIVSFLLIALAVNVAMVMTGRAIAGFCV<br>GIASLALPVYLGETVLPEVRGMLGLLPTTLGNIGILLCYVAGAYLDWSMLAFAGALIPVPFLICMFFIPE<br>TPRWYIGRNKHKKARKALQWLRGQNADISAEFDEIEKTNAESNKNEKTAGCSELFAKMYRRPLLISIG<br>LMFFQQMSGINAVIFYTVKIFKEAGSTIDGNICTIIVGIVNFGSTFVATMLIDRLGRKVLLYISSIAMIATL<br>GVLGLFFWAKERNIDVTAYGWIPLASFVIYVIGFSIGFGPIPWLMMGEILPAKIRGPAASLATSFNWSCT<br>FIVTKTFVDLLALIGSSGTFWLFTGICAVGLVFVVLFVPETQGKSLEDIERNLTGGPKVPVRQCRRMSSI<br>ANLKPLP	637
BTST121	MGKDEDEEKLSWGCWLRTAFACSGAMMAFVFNGVTEGQSAVLLPQLKEKESFIHITSEEETWIASLGI<br>LLSPVSALLIGPITDAFGRKLGLLFIHIFMGLGFAVIACATQVWHIYLGRCICSFALGLEVVSVVYMTET<br>CAKRQRSFLLSTISPAFTLGVVVAYVIGGYLPWNIASAIFALSSFVYFVVQLLAPESPAWLFKRGRIDAA<br>AWSLRKLGRSPSGIDHELQLLKLASSEESESFHLGIFLDPTVWKPFLILSLFHLVQCATGIYHIVYYTLDF<br>VTRLGTTYDPLTVSIVISVVRVISNCTIGMYFTSYVSRRFSTILSALLMTVSSGAAGVXXXGIIGVTTLPW	450

	MMSGEVFPLRVRGAMSGAVFGVGAGSMFVFIKIYEDCLALLNIWGLLFGFAIASFLTALLGIFLLPETL<br>NKTLYEIEQGFMPKEKRSNGEESTLPAEAVS	
BTST122	MSRGPYQMTGHALQPLNPTSNGLGTHTAGTMVNLPRGKSQYLSQVLAAVAISLGPLAAGLGKGYSS<br>PAIASLQGKQSWEAGHGAGAYRGHGMGHRGNYTLLTVSPQEASWVASLSLLGALFGALVGGLAMK<br>FGRKNVLLIASLPFSASWLVTVYAESVQTMFATSFVGGFCCAVVLMVSQVYISEISDPDIRGFLSAVLKIF<br>SHIGTLLSLTLGAYLDWRELAMIISGAPLLLFVSMLYMPETPSFLVLSGREPDAVRALRFLRGNDTDITR<br>ELITIRNNILTASTHQYTYRGLAHAAARLAHPILITCGLMFFQRFSGANAFQFYSVTIFSQTFNGMNPH<br>GGAIVVGFVQLLASLLSGLLIDTIGRLPLLIASSVFMSIALAGFGSFVYYEQLSRHNSYVHVQHLPPGVA<br>PPGISATYDWIPLLCVLVFTVSFSMGISPISWLLIGELFPLEYRGLGSALATSFSYACAFIGVKTYVDFTQT<br>LGLHGAFWLYAAFSLAGLCFIVCFVPETKGRDLDELDSRYI	524
BTST123	MDSTRGLRRQVTACIIANQGLFLIGINLGWSSAVNEHLLSGVLGYKYTQDQLSWAVSLLDLGTVFAPL<br>PTGYLMNKIGRKFTFLLIASLFTLSWCLKVISVQPGFLYAAQILAGVARGVGLTVTPMYSGEIAETGLH<br>GMLSTIFKLMFYSGMLLMIIVAPYMNYTTISYMGLTFSLLFFFSLFYIPDTPYYYCAVKKEREAFQSLKW<br>LRNQDKTENASVLNKELAMIKVAIEKVMGEDSGFRGLIMKPSNRRALFIVLGLFILQRMIGLNTIIGYG<br>SITLPKGHPFITPQTGMISFVVALFISSALIALFIDRIGTKPLLISSSIGCGFCTSVIAVYYWCDRTNGKAAV<br>AGFFWVPYLFFVLEAFVFSIGVGVVPTVYLSQLFPINVVGQASAASVIVASFVTFVINKAYFYVGVQFGI<br>FMMYVFFSMSAFGCAAFTHFFAIETRKKRDAEIVPVGSVIETASARK	466
BTST124	MTSSVSDEETRGKISSNEETKDKTVSDTEIGAKPVNDEECGEEVSWRCWIRTLFAASGAMMVFVFTGV<br>TEAQSAVMLPQLKKPDSYIRVGPDEETWIASLGILLAPPSGILVGPVIDAFGRKKGLLFFFLCMGLGFAV<br>IACATEVYHIYIGRCICAFAVGLEVVAVVYLAEISTKRQRSGFFSMMSVVFSGGVTLTYLIGGYLPWYIAS<br>AIFSAGCFAYFAVVCFAPESPAWLFKTGQIDASTKSFLRLGRSHVGIVAELENLKLSSKEDDEKLEFKAF<br>LEPTVWKPFVILSMYHIFQCGTGVYDILYYTVDFVESLGTSYDPLPVSILLSVARFVTTATLGIYFTASVSR<br>RFATAFSAFWMAVTLAGTGVYTYVYRDTTQKPYDWFPIVCMLINIVASALGVTSLPLLMSGEVFPLRV<br>RGAMTGASFLIGLGALFVVVKIYAFCLQILQIWGLLFVYAVFSVLCVLLGVFLLPETQGKTLWEIEQGFL<br>PKKERRRNGERRTEDTLGSGVIRK	513
BTST125	MEFATEQEPKQTPSEKWGRMFWACGGAMMIFFFNGVAESHTAVLLPRLQEPDSPIHINPDQMTWIAS<br>LGIVGAPVSGVLCGPCVDYFGRKIVVQCYFIVCALGYALIGAASSVYEIYVGRLILSLGIGFEVAGIVYIA<br>EVSTARMRSVLLSLTYSVLYGGGTLFAYVVGLSLPWNLGSAVFALACVLLFGYESFTPESPPYLVKNGH<br>TDEAIAAFKRLGRSDDQIAQEIRILERKGEPRQQVEWRTFLEPTVWKPFLIISCFHFLQAVTGVWDTLY<br>YTVDLVTNLGTQYDPYEVSLFLTVGRSLMASTAGVYFTTRVSRKMAAAVSTFSMAVSLFILAVYEKMY<br>EFTSELERPYPLLPICALIGAVMASGAGFFFLPMLMSGEVFPLRVRGTMSGAVFFVGTGSMFLFLKLHV<br>FLVTTLGVWGFYAMWTAASFITGFYSIFVLTETHGRELHEIENSYRSKKQKGADIERTSQF	474
BTST126	MATLPGKGEAPKGQELEKVAPEKWCRVLWACGGAMMIFFFSGVTEAHTAVLLPRLEEVDSPILIDAD<br>EKTWIASLGIVATPLSSVLCGPCVDYFGRKIMVQCYYLVCALGFALIASANSVYQIYAGRLICSLGIGFEV<br>AAIVYIAEVSTVRMRSVLLSLTYSVLYGGGTLFAYAVGLSLPWNLGSAVFALVCLILFGYESFVPESPSYY<br>YKKGDTKKAIVAFTQLGRTEDQIAQEIKILEERKTKTEQKVDWRTFIHPTVWKPFLIIAFFHCLQAFMG<br>LWDELYYTVDLVTELDSAYDPFEVSFILTLSRFLVASTAGVYFTTRVSRKLAAAASSFSMAVALLVVAVYE<br>KRYELTAKWERPYPLVPIVGLVGAVMASGAGMFFLPMLMSGEVFPLRVRGTMSGAVFFVGTGSMFLF<br>LKLHVFLVTTLGVPGIYTMWTTACFVAGFFAVFVLTETHGKELHEIEDSYRSKKHRSTDIERTKF	481
BTST127	MISSGKTQSPNERPYQYEYTALGTQDVEKSGEHGTGVSGNRRKVNRFRSAAPQILAVTAKNLVLLDL<br>GMTMAFSTIVVPVLLDPNNKDPNGLSFTEDQATWFASIPMVFQPLGSALSGLISAPLGRKRSLMLVNIP<br>QIIGWLMLYSSSSVNIMYLAAAIQGLGAGFMDAPIFTYVGEICEPSLRGVLISYSLQFCSVGFFLQCLLGS<br>LTTWRHVAFISMLFPTLAFLAISQIPETPMWLLSKNRMKEAEKALCWLRGWVSKEEVAEEFAQLVQYS<br>KNSKYKSDDDKKKLQMDLISTAKQPCGGCTRPPIVPCDSNTGDDDYAKLKLHEKVKDLLRPEILKPM<br>SIIIIVNFLYFTSGFPGFKTYMVLLFQRVHSPIDPNWASVFVSTSIILIHIAQMVAVKTIGKRWMTLISSFGA<br>AVAGLAIGVHMSFQGFFDETFGDLSNWLLFTYFEILTLATVIGLGPVPWMLMSEIFPFRGRSFASGFCA<br>AIYYAASFFAAKTYLSTLNLFGVAGTYYIFGTISALGLVYVYLYLPETEGLTLEEVEDIYRPKKRSEVKNL	555
BTST128	MTTDMNFGAKPESRKAILIQIISSVIASSTLLSSGMSLGFSGVALPHMEAPDSLVKVGPQEASWIASLAN<br>LATPVGCLLVGPLLDRLGRKNTMIFVGVPAVCGWLLIAVEPSLPRVYLGRLLTGLATGLSSIPSTVYTSEI<br>TSNAMRGILVTCSSISIAVGILTEYCLGWWFQRHWHCVALVSGVISILVSGLVLIGIPESPVWLVSRGQN<br>QEASKALCTLRGTKSKNKIEKELNQIIENCRAYRGRSTSIARSISGLALPQAYKPLIIMNTYFLFQQVSGL<br>FVIVFYAVDVIKIAGVTADAYLIAVLIAFLRLVTIIVSVWVNKAFGRRFASIISGVGITLSMFALVGYCYFV<br>PGAAAPTPVLVNSTTTTTAIPQALVGSTDAPIPMANFSLVENVTVVMSESFQGVHGLSWIPIAALFVHI<br>VFGTIGFLTVPWCMIGEVFPAQVRGVACSITSCFAYLSSFVVIKLYKSMLMSMGTVGIFTFYGIMSLLGTL<br>FVMIYLPETKGKSFEAIEKHFANGSGVPASPEEVSLQTKNSKQPIIRPSRPN	546
BTST129	MAEEWTXTAPPKASFLRSFLVAASMFPLYICLGALIGQSAGMLPQLLEEDSTIHINKNQATWIASLPTIG<br>TCMSSAASGYLSDLFGRIRVVQAAYSFFAIGFATMMAADSFMLLALGRFLAGIGMGCYFSGNVYLSEV<br>TPPKYRGALLTLNSVLCSCGLVYVYIVGGYYPWYIAAAATCLISIIGLTLTFSLYDSPVWLVRQNRLKTA<br>AKSLRLVEISSNVETKLRKLQETAENHPKTDFTLKILTEPSVWKPFVMILVLSILQNTSGFCIIIAYTVQFM<br>WEFHSAYDPLHVTVAIGVMRLLAILVSFVLFQHFGRKTIGAVSGFGAAIFLLGVYGYLIFAPRVQLLSEN<br>QWIPIVLFLAFIFTSSLGIYPLPWILPFELFPIKVRGMMCGACLCALYLNTFVAVMLYYVLIDNLRLGGTI<br>LLFAAGSALFGIFSMTLLVETHRRTLDDIECTFASGRVT	460

BTST130	MNEGTANLLLSQMKGATSLIHLSQDQETWVASLGILSAPIAAILIGPFIDAFGRKRGVLLFYLNMGLG<br>WAVIASAREVTQIYIGRIICAFGEGFQACAVVYLTEICTKEQRSVVLACLIALFSGGVLFVSVVNTCLPW<br>PMACSAFSLASFALAGAECFVPESPAWLFSQGEEAAAVRNLQKLGRSKAGVLLEIDALKERESCTEVL<br>SWRTFLRPTVWKPFLILAVFHLLQFSTGFYDMIYYQVDYLERLGTKYDPIALSVAFSTVRFLSNATIGIYF<br>RSLDRKFSTTVSGLCMTVPLLGAGIYELKYRDTPPLEKPFQWLLLFCIFAQLVAGNLAVTCLPWSMGA<br>ELYPLNVRGIMSGATLCVAYSIFFTYVKLYHVAMGALKVYGLLCLGLLF	394
BTST131	MFSKFMPFKSKSMSMETGNDRAIKPLICVALLIVFLAGCILGRSEDPRDEDDPYNRLKNHHDPKTFGD<br>ILYEYIRDIVKVPVIATVFFCWVCGSFADEHGRVGAMQLFFMLSGIGFGFLVYAQEYDFSILGTFILGAA<br>LGCSIPAPIYIAELCPVAYRSFFLGLVPVALSLGMFTVDVIELRGAEDTAWKSLCCFSGIGFLLSLFLHEAP<br>EWLVMRNRPDAAIESLKWLKETSVDVDVDLRKLQETSMAANHRSDTTLEMLTDKRVWKPFAMLLG<br>LALFQHLCGFYILIFYAPYLVNQYRTNIYWFSSYTGTDFLLLVATSAALVFHANLPRRTVAGLSGIGSSA<br>ALLGLFLHAHLFVAPQDLLDPTPDKDMLVPVFFFTLYIFSAVMGIYTLPWILMFEVFPLRHRGILCGLSF<br>STLYLGLFAFESRLNNYLLTGMDLQSLLCFFGTCALGFALFARSCLVETHKKTFEEIERGFTKERIFLPID<br>EKM	489
BTST132	MTDKKPQKSAHHNYKIVSTTDVELNQALGKNDSERDGSDESLPKAVSRFRSALPQVLATTAKNLILL<br>DLGMTIAFPTIVIPTLLDGKDPSGLTFNTAQASWFGSIAFICQPLGSVLSGIVLEPLGRKRSMLLVNVPH<br>LIGWILFYNAESLSILYITCALMGLGVGFMEAPIITYVGEISEPALRGILTSYSGIFVSVGFLFEYTLGNFVD<br>WRTAALISAMVPVITLIAISQVPETPTWLLSKGRNEEAKKSLQWLRGWVPVGLIMDEFDQLKRYNEAT<br>RYHAQVTSHTVSSPDVNEKPRPVSYANDVSIDDELTKPDMKTNGNAYSVNGGVSPVGAVKGRKLSFE<br>EKFYDLIRPEMVRPLGLVVVFFFIFYSCGPPGMRPYMIKLFDKMNLPVTGKRVTVIMGLIGILGNIFCMI<br>CVKWCGKRPLSLVSTAGSAASIIILGFCALDAVNGPAAVPGAQSVKWTPFVLFCSLWFFSNFGLSQIPW<br>MLTSEVFPNRGRGLASGIAAACSYLMAFVASKTYPDLERYLGIHGVCFLYGTLTLLGYIFIYFCLPETEG<br>RSLAEIEGIYSTQQKAEKKAPSS	577
BTST133	MLAFVFTGMVEAQSAVLLPQLKDKDSRIPVTPEEETWIASLGILTSPISAIISGPIVDMIGRKKGLQFFYV<br>NIGVGFGIIACATEVWHLYVGRIICAFAVGLEVVAVVYLAEICTKKQRSAIFSVMFTLAATGVLLTYVVG<br>GYLPWNIASGIFSLACFAYLIIQSLAPESPAWLFKTGRIEASIRSLQRLGRSNSGVLREVDLLKLSTQEKTE<br>KFEFRIFLQPTVWKPFLILSIYHFLATASGAYDIMAYTVEFIAALGTSYDPLAASILLSVIRVIVNATAGIYF<br>VGSVSRRLATALSALLMTISLLGTGLYSYVYREAAPGSKPHEWVPIALMILNIGAGAIGVTSLPWLMSG<br>EMFPLEVRGAMTGAAFVIGSGFMFLFIKIYYIMLEGLQMWGLLLAWAVPSAMAVAFGVWILPETQGK<br>TLYEIERGFLPQGERMREPPQVAPEQIEIDVTKNGT	458
BTST134	MDSFPIAGDLENLIPTRRPSILTFEPGVAKVLPQYLATIIVTIGGFICGTIIAWSSPAAVKLENGEDGFPVD<br>ENDMSWIGGIMPIGAILGCILTALVVDILGRKNTMIVVVLPCTIGWSLIVWADSVLMICLGRFILGTTCG<br>SFTIICPMYTAEICQKEVRGTLGTIFQLQVVSGILFLYILGSFLSLFHLSLICMVLPTVYLVFICMIPESPVY<br>HLKLGRIDEAKQSLQSLRGPNYNILTELVDLSALVDSSSETEVIPFSIAIRSPAAIKGLIIGLGVMFFQQFS<br>GVNAVIFYAASIFKDAGSSFSYNVSSIIVGSVCVAFTYLSTLIIDKLGRRVLLLFSSVVMTGCTCGLGVYFY<br>ALSHNYDMSHVQIFPILSVCGFIVAFSLGFGPIPYMLISEIFSPQIKGTASSIVCLFNWVCCFIVTKYFCILS<br>TRFGSDVTFGAFSFLSFLGIFFVYFVIPETKGKSMEEIQSHLAGSDDS	480
BTST135	MSEESDVLGEPTSPRRSNVESGMLSKKGMSTLRVVILGLIVIVPGVAPGMSFGFPAVALPQLNLNIDEAS<br>TFASLGAIAMPIGCLLSGPVIDRYGRRTALMLINLPSFMGWLLIASKPHLTRLYVARLLTGLAVGLATTP<br>AAVYSAECLTVHKMSLRGSLTTWSTVALTSGILLVYFTGALLAYTTVAIIASAISLLSLVLISLFIPESPTWL<br>IDQGRFDDAENADKILKIHRKRTASECSQLIPKDSPKEKKEEFFSMETIRKTVQDFREPEAYKPLVIMITF<br>LFFQQFSGLYVMITYMVDIISSAGVEVVNPYLVTVISGVVILIAAISVTFLLPIFGVRKLSMFSCLGVSVSM<br>LTYGMYLSVRSKVPWVSNYPIFGLIPVFAIVLNVWMSGIGFIPIPYSMLGEVFPPHVKGTAGGIASSLSSI<br>FCFIAIKTYPYLFLNLEAGIFYLYGTLALFASLFVIYYLPETRGRTLEEINSSFSSKKKSEYEQH	492
BTST136	MGTQCVMPTIVLGALRNNPDEELSLNDYDAAWLGSILFLCQPVGSIVSGFLCERFGRRGSMALINIPFI<br>VGWILLHYAASVTGLFAAALAMGMGIGFCEAPIAAYLGEIGEPHLRGSLLSIMISATSFGYLSTYFLGSI<br>MPWRTFALVNLIYPVTTMILFTQIPESPVWLVHKGRLKEAQKALGWLRGFLQPNQVQEEFDRMVKHI<br>ESSKSASPTEKKLNDFGEVTSSERSGIIGKLLLLRDPMLFQPVRLIFLTFVFTQCMCLQAFKPYLVGILKT<br>FQFPVNPKWVLIIIGLMSFVGSTMPLFIFRFTGKRNLILYNQFICCVTVFGLGIYCSFFNDTLSSDSPWRW<br>LPIVFFAIVFFSSSMGIMNVPWMLMGEVFPIRWRSFATGICGAWAYCVTFVTARLYLPMESVLSLSGMF<br>YLYGAIGILGFFYFYFFLPETEGKTLETIESYFTPYHDKKEKFSRPKR	465
BTST137	MDAPLACLMDDTKEKLVVGPPHAQIKVAELTKSTPEIRRKKGSSLRQIGAAVFANLGTINTGLVFGFSA<br>VALPQLTRPDSEIPIDENQASWLASMSSVSTPCGCILSGYLMDLIGRRRTLIVTEIPLIIGWILIGMAPNIW<br>WMYVGRLLVGLGVGMVGAPSRVYTAEATQPHLRGILAALASVHVSLGVMIEYILGYYFSWSSMAFLN<br>TLVPIGSLGACLLLPDSPAWLLSRGRFEDSKRSLQRLRGATCDVEHEMGMLVAFAQTNATGSPGSAKQ<br>TLRAILHPSARKPFLILMVYFAIYQFCGINPLTFYAVEVFQHSGSDWDKNVATIILGVVRLVFTIVGCLL<br>MRRVGRRPLTFLSSIGCGVPMLGLGYYMWLKDDWISNGVTPKFQWFPVLNIFAFMAFSSIGYLVVPW<br>VMIGEVFPAKVRGIIGGLTTCGSHFMVFVAVKSYPLMQKVLTEAGSYVFYGVISLLGTVYFYACLPETK<br>GRSLQEIEDFFSGRRESLAPDAKRRIVNNNNNRPTILKPQKGKILP	528
ApST1	MTSEKIAMTQRAMVEEQEARIGNQKWTQYLAAFIATIGGFIAGTALGWTAPAGPMMENNQYSFVIS<br>NENLAWIGACMPLGAMLGCPVTAGLVDKLGRKNMMLMLCIPTLVGWAMIIWAESVAWICAGRLLT<br>GFASGSLSVIVPLYTSEIAEKEIRGTLGTYFQLQVTGGILFTYVIGSYFNVFGLTIICAIIPIVYVALMVLIPE	489

	SPNFHLMKGNVEKARLSLRYFRGPYGTVDQELSIMQDSLAKTERERVPLMEAFQTTPAKRGLFIGLGV<br>MLLQQFSGCNAVIFYATFIFKEAGSAMEPNTSTIIVGIMSVLATYVSTLIVDRLGRKILLLSSIIVMAICTLL<br>IGAFFYMKAYEYDVSSIGFIPLTSMCVFIILFSLGFGPIPWMLIGEIFPAQIKGTACSVACMANWFFAFIVT<br>KFFSSLVSAIHIYNTFWLFTLFSILGTFFVICIVPETKGKTMDEIQEMLGAGSDLTPPTHANASIDTKEKY	
ApST4	MTEKQMVKDAEKQAPTPQNQSLNGRLLFAIIASAFGSAFQHGYNTGVVNAPQALIEKWISGVISGRN<br>DGKPTDQTQVTLIWAVAVSIFCVGGMVGGLSTGFVAEKFGRKGGLLVSNALVILSAALQGVSKMYSSY<br>ELIIIGRFIIGINSGLNAGLTPMYLAEISPMNLRGSVGTVYQLVVTISILISQILGLDYILGTAELWPVLLALI<br>IAPAIFMFATLPFCPESPKYTLINKKKDIEAERGLQWLRGTIEVHDEMDEMRAENEAMKVIPKVTLRE<br>MLSNPMLKTPLGISVMIMLCQQLSGINAVMFFSTKIFNMAGMSNDGAKYATLGMGSLNVIMTLISLFL<br>VELTGRKTLLMIGFSSMFVVTVMLTIALMFVNVSSIVSGLAVVLVMAFVIAFAVGPGSIPWFLVSELFNS<br>SARPLATSIAVGVNWTANFVVGLGFLPLQEMLQSNVFLIFVVLLALFVLYVYKKVPETKNKTLEEIQM<br>GFRQESYK	491
ApST5	MSIPEFLGGFTINLAFITNGFALAYPTIALSQLTNNGTESCSFVMSKEEGSWFAGLLGIGGICGSVFFGTL<br>IGQRIGNRKTLLLAAILDIIGWLLIAFAVNSPMMMGGRFLNGVFVGTIGPSGYTFLSEIMHRKHRASCS<br>QATSVAISAGMLVTYGLGSVISWNLLAIGCGISSVLFFIMLLTMPDSPYWNASIGKIEEAKKSLSHFRSK<br>KDDVEEEFKEIMEGIQKSIKKEKISFFEAMKLLFTDETCYKPFIILSVLFLIQTLSGLYAVIAYAIQVLEESRT<br>PIDTNLGTIISGAMRLFFGTLAIPLFFYLPRKTLMYISTGLACLSISSLGILGLLELETNTFTTYFPVGAISLY<br>MVSFTFGFQSIPFLYLGEYYPPHVRQHLAGLTSTLRFLGFFIMLKLFPQMMEFFGPNYTFIFLGLVCLFA<br>GIYAKVVLPETKGLTLNQIQDLFRTKKECSDVETNRL	465
ApST6	MATHNNMNNDDLRRLLGTSNMENEMVINKDNYNQKKELDPNYLLGDQEHIDKTTKVSWILVITTIS<br>TSLGCSIPAGYNTGVVNAPAEILKQWCNETIIQRYYVQFSPAQLDGLWSILVSVFLIGGIIGGVAGGKLA<br>NALGRKGTLQIIYLINLVSGILFFSSKSFALVELFFIARFLSGLSAGLTMAMVPMYLLELSPANKSGLFGV<br>MFTVGLNFGVVLSQFLGLGNILGNESSWHYLFSLYGGLVLLALPTLKCIPESPKYLYTVRNEHSKALSE<br>LSNLRGMPISDISWELENLLVSSERWTLRKVINEPSSRKAIIITCIIMLGQQLSGINAVFYYSTSIFRNAGM<br>STAGAQYGNLGAGVINFIVTILSTTFIDNFGRKTLLLFSSTICVFMLTALMISMILSSIGTIPGISYLLIIFVIG<br>YVLFYGFGLGPIPFFIGSELTDVGPRPIIMSAMSVANWSGNFLVGLTFPFVNLVLKQYSFLPFIVFTVFLIIF<br>TWKVVPETKQSLGQQSTDTE	516
ApST7	MLSESSYSGEMEKRPLLSSKKIPIRRSLLASFLASVMSFASGTVVGGWTAPQHPESVGEMMFMMNSME<br>ISSWVVSIYLIGALLGALPAGQLSRSIGRKKFLLLLAIPMTLGWLLITIFVNHVSLILVGRFLCGLSLGAVT<br>VAVPLYNYDVAPDVCRGRGGVFLDFMLCVGILYSYVSSALLGLRMFAFTCALFPLVFCVLFWRMPESP<br>LYLYSRGRFVDAKAALRWLQGDDCDVSAAFDEYAKLQTEDDVLPADKESQSPGRKRAFVKAVVLSLL<br>LATVQRMSGAGAIIQYTAKLFSISGSSVAPNTASIITGVFQLIGSGITIFLIDRVGRRKLLLVSSSVVVACLA<br>MLTLYFYFLNKGMLENSLKILPIVIVCTFISFFRLGLGPIPWFITTELIGADHSNRAQSCIVSYSWILSFVV<br>MKTFVMLVDEWPVALWLGYTVISVVGYLFVLFFVPETNNKSADEIRLSLAKTYQINSS	478
ApST9	MDQGVFKQLDDSCFPPKRSPDAAAGTEPGAIGPDSAKMTSWDSYLAVATFLSSPEEFMGWFVGFVSL<br>FSTNLLRPLRLALTSSNKNATKNSMLSVNGPKGTRREGKKFRQYAAALSTTIGPFAVGTVLAWTSPVL<br>PMLQSENSRIPITADEGSWVGSLIAIGAIIGSIPAGKGADIFGRKPTIAALAVPFIISWAMIYFATTVWELY<br>VARLIAGAVIGGVTATVPMYIGEIAESSIRGELGSYIQVKVTLGILYVYAIGPFVSYEGLAILCGIIPVIMFV<br>LVLLVAPETPTYLLRAGRRREAEHSLVLLRGHEYDIAGELEELQQQLEEEQNRSSKFKDLISSRATVRASI<br>AVMGLLSFLSFSGINVLIFYAESIFKSSSSSISPQVSSIIIGVLQVKFTFASALLVDKAGRRVLLLISDSVMAV<br>CLGCLGYFFWQSEHGVDVSAFSLIPLISLGVYISTFSLGFGPIPGVMMGELFSPDVKGLALGIVCVIASLL<br>EFVVVKMYQNLLDWFDHGITFWIFAGFCVLGTVFVWFLVPETKNKTLQEIQNELSGKKKSNNRKDN<br>PKGSKKHHMMDSLTGDQTHAAIV	585
ApST11	MPISEDQVAELYGEGARIHSVHHRSKPEQSVFASTMSSNASVDTTVGSTAALIHSHAAKVKKKKYAFL<br>RQGNSGGAQQSASASGNNGYLTGRHCRDAAIHSQKELNIRLATPTVPNFIDQILKKKQATEQVSAKR<br>LSSDDDDDDDDDCDDDRDDDEDDVFLQDLKSAAKIRTPQNTYVPQILASLTVSLCSMVVGFASAYTS<br>PALPSMNRPGSPLTVTEEEGSWIGSLMPLAALIGGMAGGPLIESIGRKTTILATGIPFIISFILIAMAVNVQ<br>MVMAGRAIAGFCVGVASLGLPVYLGETVQPQVRGTLGLLPTTLGNSGILLCFIAGKYLNWQMLAILG<br>ACIPIPFLVCMFLIPETPQWYISRNKSKKAKKALQWLRGKDADVTQEFSEIEKANHMGKNEEMPGYLS<br>LFSKMYSKPLLISMGLMLFQQLSGINAVIFYTVKIFKEAGSTIDENLCTIIVGIVNFLSTFIATGLIDKLGR<br>KILLYASSATMAVTLITLGTFFNYKNSGYDVSQYGWLPLASFVFFIIGFAIGFGPIPWLMMGEILPAKIRG<br>TAASLATAFNWACTFVVTKTFADLLRVFGTDGTFWMFGGICLMGLVFIIFCVPETQGKSLEDIERNLTG<br>VGKGPVRQVRRMSSIAHLKPLPMAI	646
ApST16	MMSTLTSFDETEGPDGTHKSENVQDRIPSLKEVYSEQSPLVDKTLYQTINLPQNISFGCKNKQYRTQYL<br>ATLIVTIGGFIMGTTLGWTSPAGPMMAHGQYGFPITDDDISWIASCMPLGAMLGCPFMGGLVNKLGR<br>KSLMIMLTIPALLGWAMIIWADSVTMICIGRLFNGFASGSYSVIVPQYTAEIADKEIRGTLGTYFQLQVFS<br>GILFTYVIGSYLDVFGLSIACAIVPAVYFCLMFLVPESPIFYLTKGNIIKARWSLKYFRRPFGQVDQELITM<br>QDSLAKTEREKVPIMKAFQTTPAKRGLFLGLGVMVFMQFTGCNTVIFYTTTIFNASGSTISSNVSTVIVG<br>IMAVLSTYVSTLVVDKLGRKILLLYSVIAMGICTFLIGGFFYAKDSNYDVSSIGFIPLLSLCVFIVLFSIGFG<br>PIPWMLMGEIFPPQIKGIASSIVCMANWFFVFLATKFFSLLVSTIYLYNTFWLYTLVSVLGTFFVVFIVPET<br>KGKTMEEIQLLLGA	508
ApST17	MHHIFESKTNGISDYDDHVASSFRRPSDEDEIPKNIYSEKSPLVDKKVPKSITIAQQIAAESIETQIKTQKR	528

	NQYLAALIATIGGFIMGTTLGWTAPAGPMMENGQYGFQITVENVSWIASVMPLGAMLGCPVMASLV<br>NKLGRKHLMIMLTIPTLFGWAMIIWAKSVVWICAGRFLTGFSSGSYSVIVPLYTSEIAEKEIRGTLGTYFQ<br>LQVNAGILFTYVVGSYLNVFGLSVACAIVPVIYICLMFLIPESPIFYLMKKNVEKAQLSLKYFRKPVVHV<br>NQELNTMQSALAKTERERVPIMEAFQTTPAKRGLCLGLGVMVFQQFTGCNAVIFYATTIFNATGSSIG<br>SNTSTIIIGIMAVVSTYVSTLVVDKLGRKILLLYSVVAMGICTFLIGGFFYAKESHYDISSIGFIPLMSLCIFII<br>LFSIGFGPIPWMLMGEIFPAQIKGIASSVVCMSNWLFVFLVTKFFTLMVSAIYLYNTFWLFTLFGVLGTF<br>FVVFFVPETKGKTMEEIQELLGADHITLLTENQNDA	
ApST21	MSKKGVYRQTYVTVVATMSIFISGMWLGWPSSVVEKFVNHKTDFNVTMDELSWIVATMDLGNMISP<br>LMAGHLMDWMGRKLSIVVLGPLFIVSWALTLFVPTPWALYTARLLAGMGKGMSYTVVPVYLGEIASP<br>AIRGGLGSVFCLQLHCGLLMESIIGPLVSYRTLNVVSAVVPVLFFAAVVWVPESPYYLLKRNRRPQAAV<br>CLQWFRGGGDVVHELDQMEVNVRKEMENRSTFQELFASRKDMRALSIVVAACAAQRGGGISCILAY<br>SSLILPDNGPLLNKHQSIMLFGVTMVVVNFVAVALVDRVGRKPLLLLSEAGMAVLTLTFAVFFYCSRG<br>DGSDWASRELAWLPYLCHWSFAVMFATGVGFVPVVFLGEMFPVNIRSHCSAIASITLAFCSFVTNKMF<br>LFVSDRYGVHAMFLLFTVVNLVGTFYTYKYAIETKGKTLQEIQEQLQDTVGPRRGKTNQNDN	466
ApST22	MDRKKFRENDRLIGTTKEEEAKSGHRQLLACFLASLMSLSAGTVIAGWSPTEGNSKEEDLKMWSTEQ<br>ESWIISIYVVGALIGALPAGFLGQKYGRKTLLLWLAAPMIAGWILCLLRLESLFLECLGRFVCGISVGAT<br>TVAVPLYAREVSSDVLRGRTGVFLDFMLCVGILYAYVARTVLDGVRQFCLACAVVPVTFVVLFAYVPES<br>PVHLYSVGQYEQAASALRWLRGRWFNVKKEFDQIETSKCLDDELFDRVRKMSDLNKKFLAKVTIISF<br>GLVLVQRMSGAGGVIQYSSTLFKMSGSTIEPNTACIIVGTFQLVASGVSFLLVDKVGRRTLLLTSSAVITT<br>CLSLLVVYFSLIEKETQIESPWRISLLFILCVFISAFRLGLGPIPWFISTELSPASYGSRIQSMAACFSWSLSF<br>VIMKTFKIFVEANPVLLWFTFAAISAAGFLFVLFYVPETNNKSREQIHIELIG	471
ApST23	MHSVKMTPKMGFGHVYLTLAACMSSLFQFLRWFIGSVIFAHNRNPLLLSLTSSNKSASDGTPSNEPQG<br>KKFRQYVTALSATVGPFAVGTVLAWTSPALPMLLSADSTIKITPDQGSWVGSLIAIGAIFGSIPAGKTAD<br>LIGRKPVIAFLPLPFITSWLLIYFAKDVWYLYVARLVAGTCLGAITATVPMYIGEIAEKSIRGELCSYVQV<br>NVTLGILYVYSIGPFVNYAWLAIMCGILPVIWFILVLLVLPESPTYLWRSGKNKEAEDVLVMLRGKDYDI<br>SGELQALQKELEEKKPNGKLKDMVKSKATLRAAFTALGLFGFLSCSGINVVIFNAQTIFSSTGSIVSPKT<br>SSIVIGILQVIFTFTSSQLVDRAGRRVLLLISDSVMAVCLGSLGFYFWQLEHGVDTSVFSLVPLISLGVYIST<br>FSLGFGPIPGVMVGELFSPEFKGLAIGIVCVLASLIEFSVVKSYQTLLDNYGRGVTFGVFAGCCVMGTLF<br>VLFLVPETKNKSLQEIQDELSGKKKSEQKQGPSGS	527
ApST24	MFSVVYDKIPQIVFALSASLGAFVIGTVLGWSSPTLTMFENGTAVSFEVSAMAAATACSLFGVGAVIGA<br>VPAGAVSSVFGRRVSLIVSEAHVVFGWLMIAYPKAARMLYVGRILQGVGCGAMCTIIPMYVGEIAEPEI<br>RGFLGGLYQLFVVSGILYSYVLGNFLNYNQLNLACGVWMAVHILGVLYIPESPYFLIQENKRVGAEEA<br>MARLRDPSHDCKSELDEIQKFVEEEQKNSYTAREVLEKDVNRRALTIGIGCMFFQQMTGINAIIFYMK<br>HVFEISGSDISPEVCTTVVGTIQVAMTFASMMITDKFGRRSLMVYSMTLMGVCLLALSYYFFSKKYNPH<br>VAETLDWLPLVAIVLYISMFSIGCGPIPYIIIGEIFSSELKSMGTGMSIATNWILVWLVTCLAEPMDKFIGPS<br>GTFFVYSGFCFMGMLFVVNCVPETKNRSLAVIQSDLEKN	455
ApST25	MVLPDRHEKSKVFTLIYNLSGGAINSQIIITVIAALGAIATGTILGWSSSAQSMFDADDSLLPFAVTGKD<br>TQTFSSVFGIGAALGALPAGYVSRLFGRPASMLLFEGFLLVGWAMLVLPTSVWMLSAGRMMQGIGVG<br>ALCAIIPSYIGEIAEPRMRGRLGTIFQLFIVIGILYSYTSGAFMKYVPFCVACAFWVILHFIGALCIPESPYH<br>LMNINDPDGAAVSLQILRDSSDTTEELASIKLFVEKQQSQSYTVSEVLSDKVNRKALMISIGCMFFQQM<br>SGINVVIFYMTDIFKSTGSNMSPNTCTIVVGVVQLFMTVLSFTIIDKSGRKALLVLSGLLMANCYMGLG<br>GFFLIKTHYLELASKLNWLPLVCIAVYISAFSIGYGPVPWIMMGEIYSSEVKPIGTSLTTCTNWTLVFVVT<br>YVSTELIRWLGQAGCFLTFSAFCLMGAAFAASVVPETKNKTLAEIQLKLVGKSKAVPVAVDVVEATEP<br>VQATTIS	495
ApST26	MSKGVRNQIIFSLIATLGAMVMGTILGWTSPANTLLQNGVGFPISVDDLKSFSSIFGIGAACGALPAGK<br>LSATIGRRYSMVLFEIIIIIGWIFLTMANASWMLLAGRVLQGVGVGALCTVIPTYVAEISQPHIRGTLGTI<br>FQVYVVIGILYSYIIGSVVEYHTFNVLCGIWTIIHVLLTFFVPESPYFFMYKNKDKNANTSMMKLRDGN<br>DADIAGELTVIKTEIELQKANQDTFTKVMSNKANRKSLLIGIGCMFFQQTSGINAIIFYMAYIFNEIGSSI<br>TTNTSVIAVGIVQLVMTFVAMMIVDKAGRRVLLIVSAIVMSISFFCLGLYLEYRKSVHKDSILSWLPLILI<br>ALYISAFSLGFGPIPWVVMGEIFSNEVKPYGTSLATATNWILVFAVTFLTFVTTNSLGFLGLFWMFSLFC<br>ALGALFVWYTVPETKNKSLTEIQLKLAGNDNSPSSTTDV	460
ApST27	MHNFLGSKFKGNCRQLFAAITVNLVTFSFGLYTGWSSTVAPILQNATVTPLDHGQVLTANSISWACSW<br>GMLSAILGTFFWGMLADNCGRKTTGFLTMLPYLVSWVILLVFKTETALMVSRFLGGLGASGAAINCP<br>MYVGEVSETSMKAGLGSLFILMYNIGVLYVYVFGVMVSYDFLNVACLAISVLFMVVWCYVPESPIFLIQ<br>KNRMDEARRSLMWFRGKDNDKEVSEEIDSLMRHSDQTTKATLADYKKRGTVKALLIGLVFQAGTQF<br>SGINIILMYTVDIFQKSGSTMSPHSCTILVGVVQVIGSAIASCTVHRAGRKFFLMATYAITALALITIGSCF<br>YANKVDSTINTGMLPVLSLSVHVIAFSLGLGMVPYIIYTEVFPANVRNICMSMLMFFNNVLGFVIIKAY<br>PSMSDALHISGYFWLFGAVCLAVVPFTYLFVPETKDKAYDDIRRELLLWFPDRRHNNKAAEVAKAEK<br>AAAVDQVDGGGGNVVVVEASEMKVCMGKDQSCFTTPQQARDNVKK	523
ApST28	MFKKGVYRQIYVTIVATMSIFISGMWLGWPSSVVEKFVKHETDFNATMDELSWIVATMDLGNVISPLM<br>ASHLMDWMGRKLSIVVLGPLFIVSWALTLFVPTPWALYTARLLGGMGKGMSYTVVPVYLGEIASPAIR<br>GALGSVFCLQLHFGFLMEAVIGPLVSYRTLNVVSAVVPVLFFVAAVWLPESPYYLLKRGRRPQAAVCL	466

	QWFRGGGDVVHELDLMEVNVRKEMENRSTFQELFASRKDMRALAIVVAACATQRGGGISCILAYSSL<br>ILPDNGPLLNKHESVMLFAVTLAVVNLVAVALVDRVGRKPLLLLSEAGMAVLTLTFAVFFYCSRGDGS<br>DWASRELAWLPYLCHWSFAVMFATGVGFVPVVFLGEMFPVNIRSHCSAIASITLAFCSFVTNKMFLFV<br>SNRYGFHAMFLLFTVVNFAGTFYTYKYAIETKGKTLQEIQEQLQDTVGRRREKTNQNDN	
ApST43	MVAIKNELDTVQQVKEEIEELREYVDRLELQSHRRKLRLLEQGLTFFLSYTILASMLGMLQFGYNTGVI<br>NAPEGNIEKFIKDVFEDRYKENMDHGQAELLYSFAVSIFAIGGMLGGFSGGIIANRFGRKGGLLLNSFV<br>GIGGACLMGLTKYFNSYEVLFIGRFIIGVNCGLNTSLVPMYISEIAPLNLRGGLGTVNQLAVTTGLLISQI<br>LGIEQILGTDEGWPLLLGLAICPAILQLILLPVCPESPRYLLITKQWEEEARKALRRLRATNQIEEDIEEM<br>RAEERAQQSEATISMMELVCSPTLRQPLIISVVMQLSQQLSGINAVFYYSTSLFITAGLAENVAKFVTIGI<br>GVIMVNMTLVTMPLMDKTGRRTLHLYGLGGMFIFSIFITISLLITEFFGFVQEMIDWMSYLAVVSILGFV<br>VFFAVGPGSIPWMITAELFSQGPRPAAMSIAVLINWVANFAVGIGFQPLKTALDNYTFLPFSVLLAIFWI<br>FTYKKVPETKNKTFEEILALFRQNGRGSVLESSRLYGTSTTLSDGPGGVCCMRQHWQFPHDDVSERNS<br>PVESHAQ	566
