# Supplementary material for: Genome-Wide Identification and Expression Pattern of Sugar Transporter Genes in the Brown Planthopper, Nilaparvata lugens (Stål)
Source: Insects. 2024 Jul 7;15(7):509. doi: 10.3390/insects15070509 (PMC11277001; doi:10.3390/insects15070509)
Supplement: Supplementary file 1 [file Supplementary Figure S1.pdf › Supplementary Figure S1.pdf]

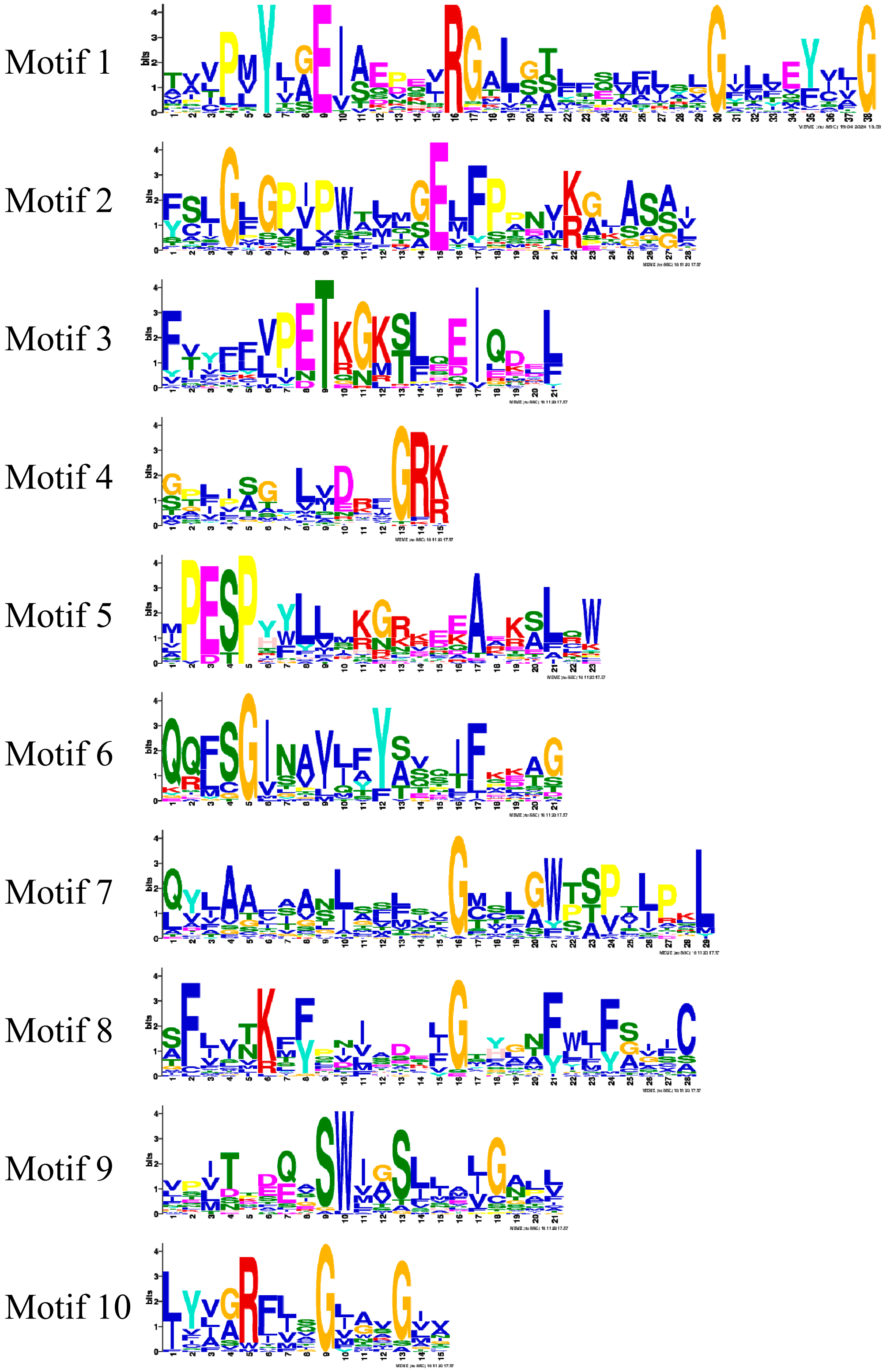

Figure S1. Sequence logos for 10 motifs of sugar transporter domains using the MEME program. MEME motifs are displayed by stacks of letters at each site. The total height of the stack is the 'information content' of that site in the motif in bits. The height of each letter in a stack is the probability of the letter at that site multiplied by the total information content of the stack. The x-axis represents the width of the motif and the y-axis represents the bits of each letter.
